# Caspase 3/GSDME-dependent pyroptosis contributes to chemotherapy drug-induced nephrotoxicity

**DOI:** 10.1038/s41419-021-03458-5

**Published:** 2021-02-15

**Authors:** Xiujin Shen, Haibing Wang, Chunhua Weng, Hong Jiang, Jianghua Chen

**Affiliations:** 1 Kidney Disease Center, The First Affiliated Hospital, College of Medicine, Zhejiang University, Hangzhou, China; 2 Key Laboratory of Kidney Disease Prevention and Control Technology, Hangzhou, Zhejiang Province China; 3 National Key Clinical Department of Kidney Diseases, Hangzhou, China; 4 Institute of Nephrology, Zhejiang University, Hangzhou, China; 5 The Third Grade Laboratory under the National State, Administration of Traditional Chinese Medicine, Hangzhou, China; 6 Central Laboratory, The First Affiliated Hospital of Zhejiang Chinese Medical University, Hangzhou, China

**Keywords:** Apoptosis, Acute kidney injury

## Abstract

Chemotherapy drug-induced nephrotoxicity limits clinical applications for treating cancers. Pyroptosis, a newly discovered programmed cell death, was recently reported to be associated with kidney diseases. However, the role of pyroptosis in chemotherapeutic drug-induced nephrotoxicity has not been fully clarified. Herein, we demonstrate that the chemotherapeutic drug cisplatin or doxorubicin, induces the cleavage of gasdermin E (GSDME) in cultured human renal tubular epithelial cells, in a time- and concentration-dependent manner. Morphologically, cisplatin- or doxorubicin-treated renal tubular epithelial cells exhibit large bubbles emerging from the cell membrane. Furthermore, activation of caspase 3, not caspase 9, is associated with GSDME cleavage in cisplatin- or doxorubicin-treated renal tubular epithelial cells. Meanwhile, silencing GSDME alleviates cisplatin- or doxorubicin-induced HK-2 cell pyroptosis by increasing cell viability and decreasing LDH release. In addition, treatment with Ac-DMLD-CMK, a polypeptide targeting mouse caspase 3-Gsdme signaling, inhibits caspase 3 and Gsdme activation, alleviates the deterioration of kidney function, attenuates renal tubular epithelial cell injury, and reduces inflammatory cytokine secretion in vivo. Specifically, GSDME cleavage depends on ERK and JNK signaling. NAC, a reactive oxygen species (ROS) inhibitor, reduces GSDME cleavage through JNK signaling in human renal tubular epithelial cells. Thus, we speculate that renal tubular epithelial cell pyroptosis induced by chemotherapy drugs is mediated by ROS-JNK-caspase 3-GSDME signaling, implying that therapies targeting GSDME may prove efficacious in overcoming chemotherapeutic drug-induced nephrotoxicity.

## Introduction

Traditional chemotherapeutic drugs, such as cisplatin and doxorubicin, are commonly used to treat various cancers, including lung, bladder, and ovarian cancer^1–4^. However, severe side effects caused by toxicity to healthy organs and tissues, particularly the kidney, limit the clinical application of these drugs^5,6^. Indeed, chemotherapeutic drug-induced nephrotoxicity reportedly occurs in one-third of cancer patients^7^, the mechanisms of which have been wide studied^8–10^. Tubular injury, inflammation, and vascular injury are typical characteristics of chemotherapy drug-induced nephrotoxicity, among which, tubular injury is the most critical. In the kidneys, chemotherapeutic drugs cause proximal tubular cell death, leading to acute kidney injury (AKI)^11^. However, the associated molecular mechanisms have not yet been fully characterized. Therefore, further studies are warranted for the early diagnosis and treatment of chemotherapeutic drug-induced AKI.

Pyroptosis is a newly discovered form of programmed cell death with morphological characteristics that differ from those of apoptosis and necrosis^12^. Pyroptosis can be induced by activation of the executors, gasdermin E (GSDME), or gasdermin D (GSDMD), which results in the cleavage of their N-terminal fragments (GSDME-N or GSDMD-N, respectively)^13–15^. GSDME-N or GSDMD-N then translocate to the cell membrane and mediate cell perforation, resulting in infiltration of extracellular material, cell swelling, and pyroptosis^16^. Moderate cell pyroptosis can remove pathogenic microorganisms and antagonize infection, however, excessive cell pyroptosis not only leads to cell death but also enhances inflammatory responses, resulting in fever, hypotension, septicemia, as well as other serious symptoms^12^.

Pyroptosis is associated with diabetes, as well as infectious, metabolic, nervous, and cardiovascular diseases^17–20^. Moreover, recent studies have indicated that GSDMD-dependent pyroptosis is also associated with kidney diseases, especially AKI^21–23^; hence, pyroptosis has become the focus of considerable kidney disease research. The results of these studies have demonstrated that renal tubular epithelial cell pyroptosis can accelerate ischemia-reperfusion and contrast-induced AKI. Specifically, Zhang et al.^21^ found that the caspase 4/5/11 signaling pathway promotes contrast-induced AKI by inducing GSDMD-dependent pyroptosis of renal tubular epithelial cells, and caspase 11 knockout mice exhibit reduced AKI damage by inhibition of GSDMD activation. In addition, Wu et al.^24^ reported that miR-155 promotes pyroptosis of renal tubular epithelial cells through caspase 1, thereby accelerating ischemia-reperfusion-induced renal damage.

The effect of GSDME, a newly defined executor of pyroptosis, has recently been reported in various cancers, with strategies targeting GSDME proposed to block pyroptosis^25–27^. Wang et al.^14^ reported that GSDME-positive tumor cells switch cisplatin-induced cell death from apoptosis to pyroptosis, resulting in extensive inflammatory damage. In addition, GSDME knockout attenuates cisplatin-induced crypt and villi disruption and attenuates the reduced spleen weight and lung injury. Further studies found that caspase 3, an executor protein of apoptosis, serves as the primary protein responsible for GSDME cleavage and activation, implying that GSDME, in addition to GSDMD, plays an essential role in pyroptosis. However, to the best of our knowledge, the role of GSDME in chemotherapeutic drug-induced nephrotoxicity has not been reported to date.

To clarify the relationship between GSDME and chemotherapeutic drug-induced nephrotoxicity. We treated human renal tubular epithelial cells with the chemotherapeutic drugs, cisplatin, or doxorubicin, to determine the role of GSDME in cell pyroptosis. This study will provide new insights into the role of GSDME-dependent pyroptosis in chemotherapy-induced nephrotoxicity.

## Results

### Cisplatin or doxorubicin induces pyroptosis of renal tubular epithelial cells

It has been reported that the typical characteristics of pyroptosis were increased LDH release, increased PI-positive cells with flow cytometry, and typical bubbles emerging from the cell membrane^14^. We treated human renal tubular epithelial cells, HK-2, with various concentrations of cisplatin (0, 5, 10, 20, and 40 μM) or doxorubicin (0, 0.5, 1, 2, and 4 μg/ml). CCK-8 and LDH analyses indicated that cisplatin and doxorubicin decreased cell viability and increased LDH release in a concentration-dependent manner (Fig. S1a–d). Flow cytometry analysis demonstrated that cisplatin dramatically increased the proportion of propidium iodide (PI)^+^-positive cells in a concentration-dependent manner (Fig. S1e, f) Morphologically, both the cisplatin- and doxorubicin-treated HK-2 cells showed typical bubbles emerging from the cell membrane (Fig. S1g). Therefore, these data indicate that cisplatin and doxorubicin induce pyroptosis in human renal tubular epithelial cells.

### Cisplatin or doxorubicin promotes GSDME cleavage in the kidney in vitro and in vivo

**Fig. 1:**
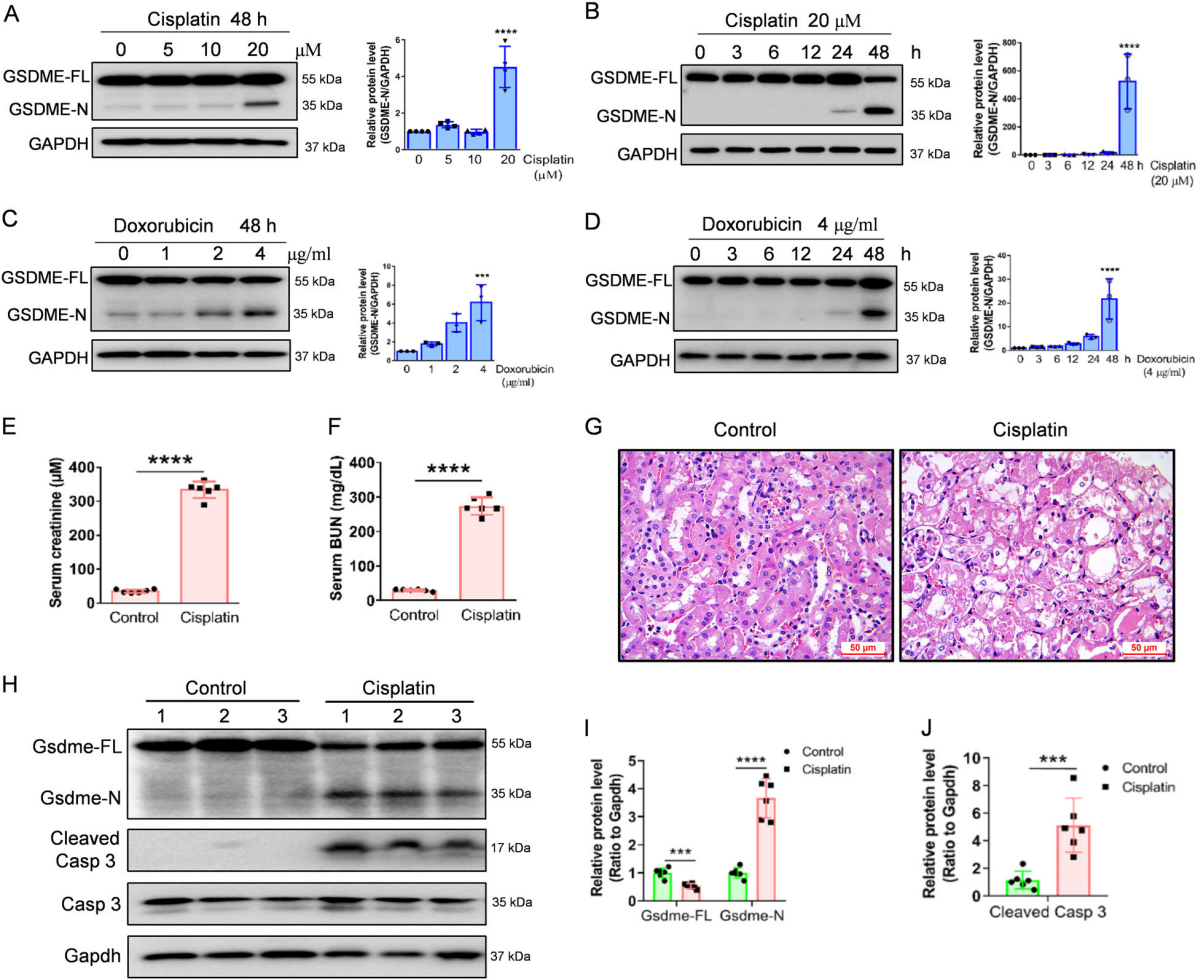
Cisplatin or doxorubicin induces cleavage of GSDME in renal tubular epithelial cells in vitro and in vivo. **A**, **B** Western blot analysis of GSDME in HK-2 cells treated with various concentrations of cisplatin (0, 5, 10, and 20 μM) for 48 h, and with 20 μM cisplatin for different times (0, 3, 6, 12, 24, and 48 h). **C**, **D** Western blot analysis of GSDME in HK-2 cells incubated with various concentrations of doxorubicin (0, 1, 2, and 4 μg/ml) for 48 h and 4 μg/ml doxorubicin for different times (0, 3, 6, 12, 24, and 48 h). **E**, **F** Serum creatinine and BUN detection of normal and cisplatin-treated mice after 72 h. **G** Hematoxylin–Eosin (HE) staining of control and cisplatin-treated mice. Scale bar, 50 μm. **H**–**J** Western blot analysis of Gsdme in mice and caspase 3 in control and cisplatin-treated mice. All data are presented as means ± SD from at least three independent experiments (*n* = 3 for in vitro experiment; *n* = 6 for in vivo experiment). ***p < 0.001 versus control group, ****p < 0.0001 using two-tailed Student’s *t* tests.

Cell pyroptosis can be triggered by the cleavage of the Gasdermin family proteins^13,14^. Our immunohistochemical results demonstrated that GSDME is positive in renal tubular epithelial cells of normal human kidney (Fig. S1h), which was consistent with the expression in the Human Protein Atlas. In addition, both cisplatin and doxorubicin-induced the cleavage of GSDME in a concentration- and time-dependent manner (Fig. 1A–D). We, therefore, postulate that GSDME is involved in cisplatin- and doxorubicin-induced pyroptosis of human proximal tubular epithelial cells.

We then examined GSDME cleavage in a cisplatin-induced mouse model of nephrotoxicity and found that cisplatin increased serum creatinine and BUN (Fig. 1E, F). HE staining exhibited severe renal tubular epithelial cell death in cisplatin-treated mice compared to the control mice (Fig. 1G). Western blot detection indicated that cisplatin increased the cleavage of GSDME and caspase 3 activation (Fig. 1H–J).

### Caspase 3 activation is associated with GSDME cleavage in cisplatin- or doxorubicin-treated renal tubular epithelial cells

Recent studies have indicated that GSDME is an executor protein of pyroptosis owing to its activation of intrinsic and extrinsic apoptotic pathways^14,28^. Our results show that the levels of activated caspase 3/7/8/9, PARP, and Bax were elevated, while that of Bcl-XL was reduced in a concentration- and the time-dependent manner in response to cisplatin or doxorubicin induction. No activation of caspase 6 was observed after cisplatin or doxorubicin treatment (Fig. S2a–d).

**Fig. 2:**
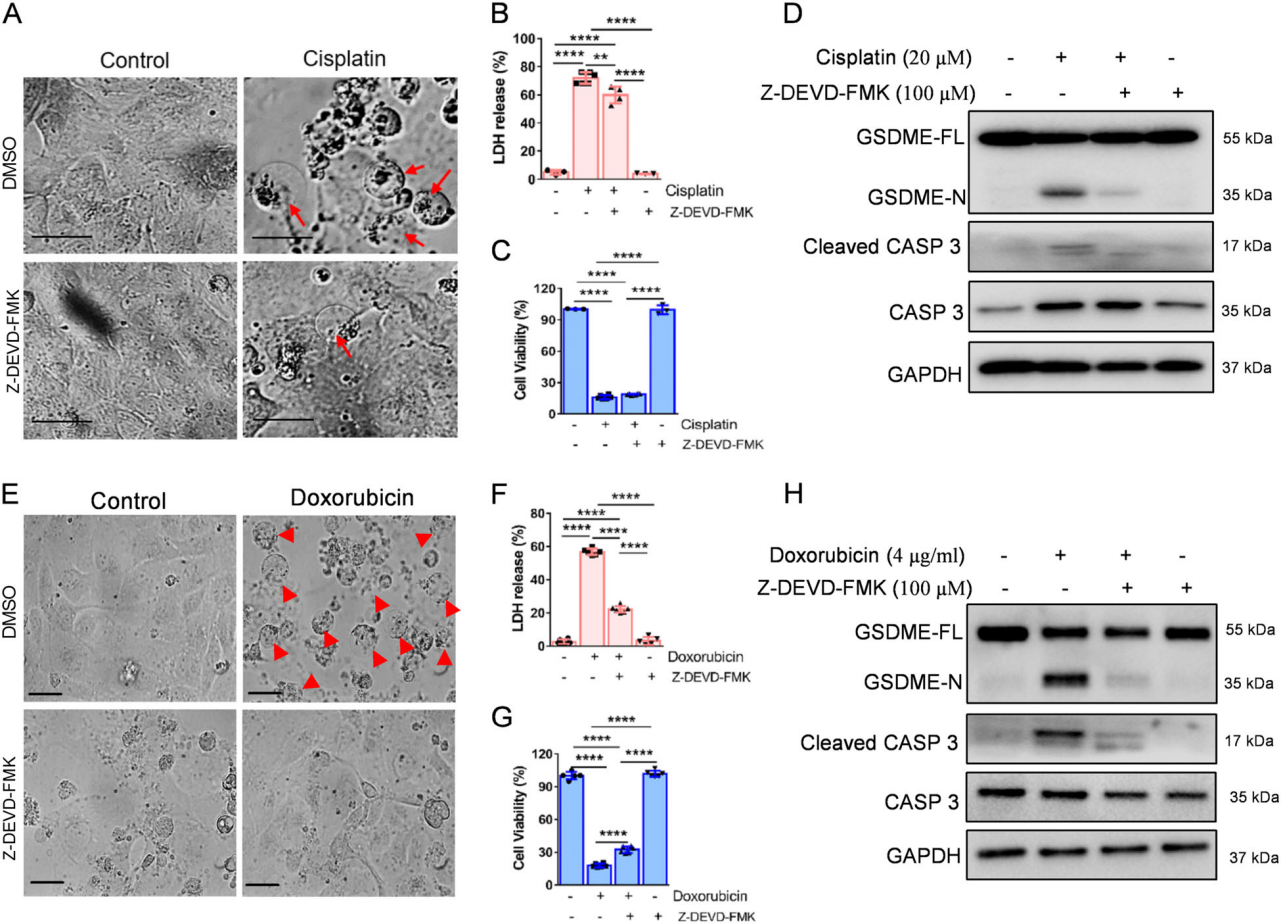
Z-DEVD-FMK decreases cisplatin- or doxorubicin-induced pyroptosis in HK-2 cells. **A**, **E** Representative light microscopy images of HK-2 cells treated with cisplatin (20 μM) or doxorubicin (doxorubicin, 4 μg/ml) before or after Z-DEVD-FMK (100 μM) intervention. The red arrow shows bubbles emerging from the plasma membrane. Scale bar, 50 μm. Cytotoxicity and cell viability were detected using the LDH assay (**B**, **F**) and CCK-8 detection (**C**, **G**) in HK-2 cells induced by cisplatin (20 μM) or doxorubicin (4 μg/ml) in the presence or absence of Z-DEVD-FMK (100 μM). Western blot analysis of GSDME and caspase 3 (CASP 3) cleavage in cisplatin-treated (20 μM) (**D**) and doxorubicin-treated (4 μg/ml) (**H**) HK-2 cells in the presence or absence of Z-DEVD-FMK (100 μM). All data are presented as mean ± SD from three independent experiments (*n* = 3). *p < 0.05, **p < 0.01, ***p < 0.001, ****p < 0.0001 using one-way ANOVA followed by the Tukey’s method.

**Fig. 3:**
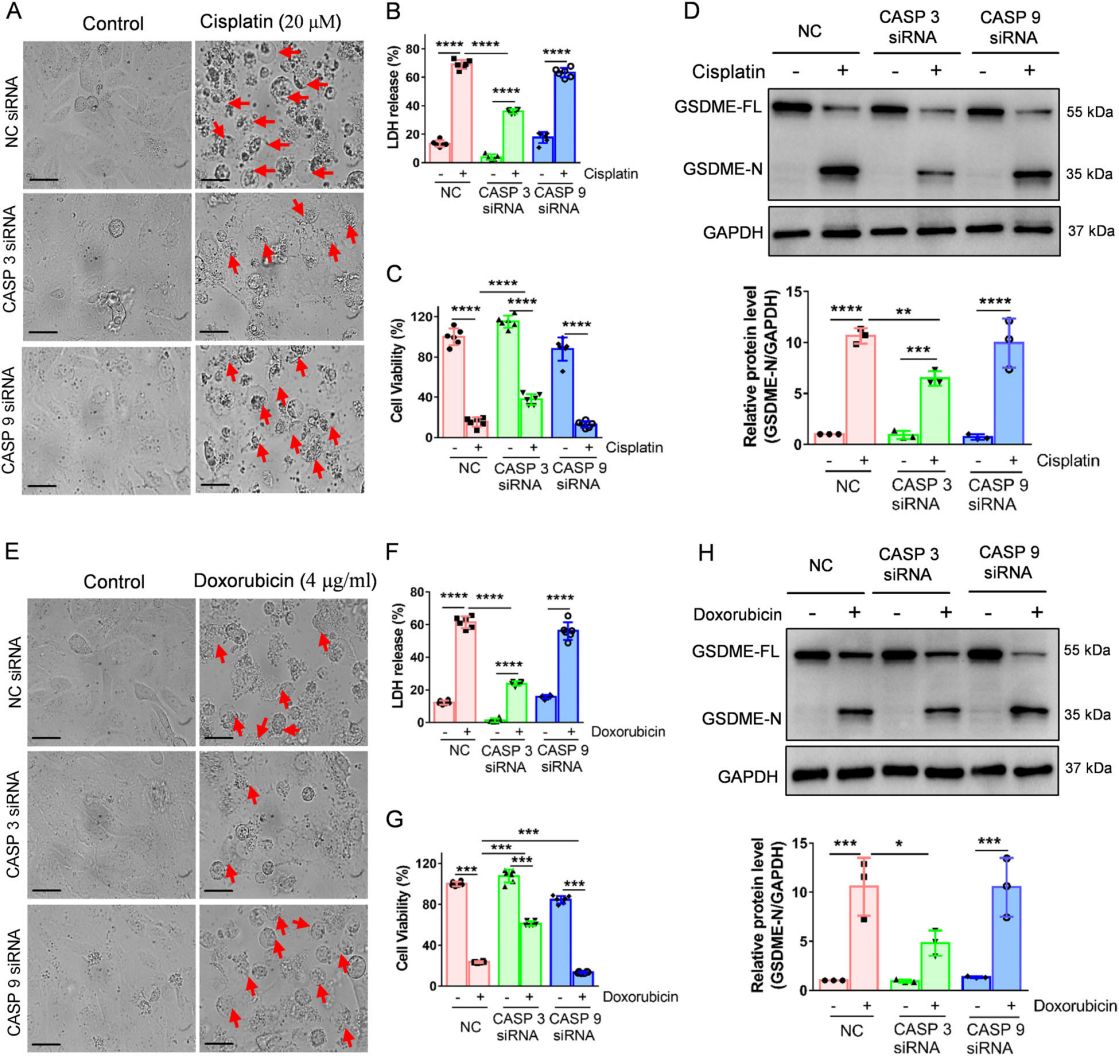
Caspase 3, rather than caspase 9, contributes to cisplatin- or doxorubicin-induced pyroptosis in HK-2 cells. **A**, **E** Representative light microscopy images of HK-2 cells treated with cisplatin (20 μM) or doxorubicin (4 μg/ml) in the presence or absence of CASP 3 or CASP 9 siRNA. A red arrow indicates bubbles emerging from the plasma membrane. Scale bar, 50 μm. Cytotoxicity and cell viability were determined using LDH assay (**B**, **F**) and CCK-8 detection (**C**, **G**) for HK-2 cells treated with cisplatin (20 μM) or doxorubicin (4 μg/ml) in the presence or absence of CASP 3 or CASP 9 siRNA. **D**, **H** Western blot analysis of cleavage of GSDME in cisplatin- (20 μM) or doxorubicin- (4 μg/ml) treated HK-2 cells in the presence or absence of CASP 3 or CASP 9 siRNA. All data are presented as mean ± SD from three independent experiments (*n* = 3). *p < 0.05, **p < 0.01, ***p < 0.001, ****p < 0.0001 using one-way ANOVA followed by the Tukey’s method.

To further verify the connection between the caspase cascade and GSDME cleavage, we firstly pretreated HK-2 cells with the caspase 3-specific inhibitor, Z-DEVD-FMK. The results indicate that GSDME cleavage and LDH release were significantly inhibited, while cell viability was partially ameliorated following treatment (Fig. 2A–H). Moreover, pretreatment of cells with the caspase inhibitor, Z-VAD-FMK, showed similar results (Fig. S3a–h). We then knocked down the expression of caspase 3/7/9 in HK-2 cells (Fig. S4a–c). Morphologically, the pyroptotic features in the cisplatin- or doxorubicin-induced HK-2 cells were abrogated following caspase 3 siRNA intervention (Fig. 3A, E). Cell viability was increased and LDH release was suppressed after caspase 3 siRNA treatment (Fig. 3B, C, F, G). The western blot results indicated that caspase 3 siRNA inhibited GSDME cleavage induced by cisplatin or doxorubicin (Fig. 3D, H). Interestingly, we found that caspase 9 siRNA did not affect the cisplatin- or doxorubicin-induced pyroptosis (Fig. 3A–H). Caspase 7 knockdown augmented the cleavage of GSDME and caspase 3 induced by cisplatin and doxorubicin (Fig. S4d–k), suggesting that caspase 7 knockdown induces other caspase-related proteins, which may increase caspase 3 cleavages, leading to augmentation of GSDME cleavage.

Necroptosis also reportedly plays an essential role in cisplatin-induced death of HK-2 cells^29^. To distinguish necroptosis from pyroptosis, we used GSK’872 (a necroptosis inhibitor) to block necroptosis. The results demonstrated that GSK’872 did not affect the cleavage of GSDME nor prevent the typical morphology of pyroptosis (Fig. S5a–d), implying that GSDME activation is not associated with necroptosis.

### GSDME inhibition attenuates cisplatin- or doxorubicin-induced pyroptosis in the kidney in vitro and in vivo

**Fig. 4:**
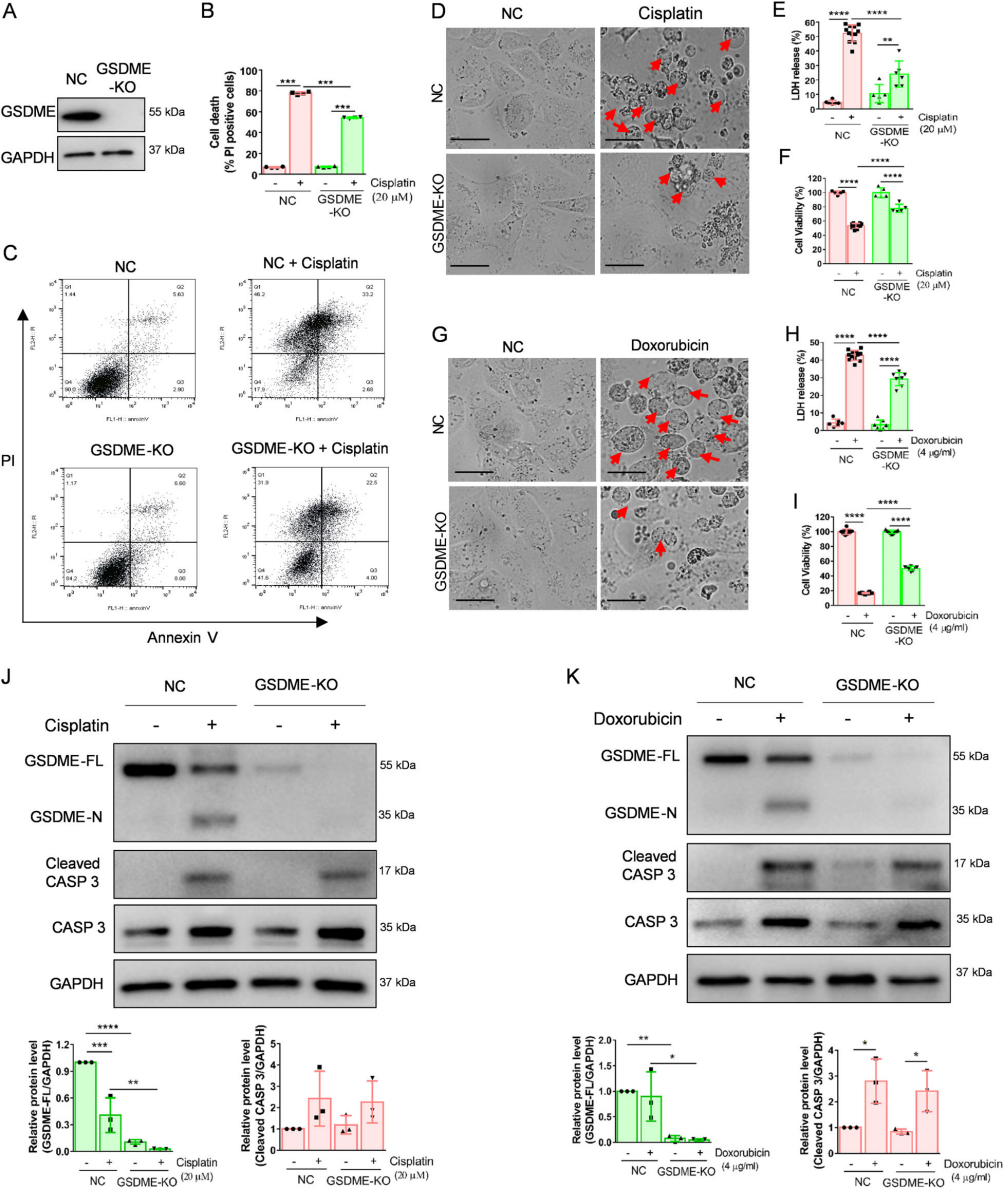
GSDME knockout alleviates cisplatin- and doxorubicin-induced pyroptosis in HK-2 cells. **A** Western blot analysis of GSDME expression in HK-2 cells treated with px459-GSDME-KO plasmid. **B**, **C** Percentage of PI^+^ HK-2 cells were detected by flow cytometry in the normal group (NC) and GSDME knockout group (GSDME-KO) treated with cisplatin (20 μM) or doxorubicin (4 μg/ml). **D**, **G** Representative light microscopy images of HK-2 cells treated with cisplatin (20 μM) or doxorubicin (4 μg/ml) in NC and GSDME-KO group. The red arrow indicates bubbles emerging from the plasma membrane. Scale bar, 50 μm. Cytotoxicity and cell viability were determined using the LDH assay (**E**, **H**) and CCK-8 detection (**F**, **I**) in NC and GSDME-KO HK-2 cells treated with cisplatin (20 μM) or doxorubicin (4 μg/ml). **J**, **K** Western blot analysis of cleavage of GSDME and caspase 3 in cisplatin- (20 μM) or doxorubicin- (4 μg/ml) treated HK-2 cells in NC and GSDME-KO group. All data are presented as mean ± SD from three independent experiments (*n* = 3). *p < 0.05, **p < 0.01, ***p < 0.001, ****p < 0.0001 using one-way ANOVA followed by the Tukey’s method.

To clarify the effect of GSDME cleavage on cisplatin- or doxorubicin-induced pyroptosis in renal tubular epithelial cells, we generated GSDME knockout (GSDME-KO) HK-2 cells. The efficiency of the GSDME knockout was verified by western blot (Fig. 4A). Flow cytometry analysis indicated that GSDME-KO dramatically decreased PI^+^-positive HK-2 cells following cisplatin treatment (Fig. 4B, C). Morphologically, GSDME-KO decreased the pyroptotic features of cisplatin- or doxorubicin-treated HK-2 cells (Fig. 4D, G). Furthermore, CCK-8 and LDH analyses indicated that GSDME-KO increased cell viability and decreased LDH release induced by cisplatin or doxorubicin in HK-2 cells (Fig. 4E, F, H, I). However, caspase 3 cleavage was not affected in the GSDME-KO group compared to that in the empty vector (NC) group (Fig. 4J, K). Taken together, these data imply that GSDME is vital in cisplatin- or doxorubicin-induced pyroptosis in HK-2 cells.

**Fig. 5:**
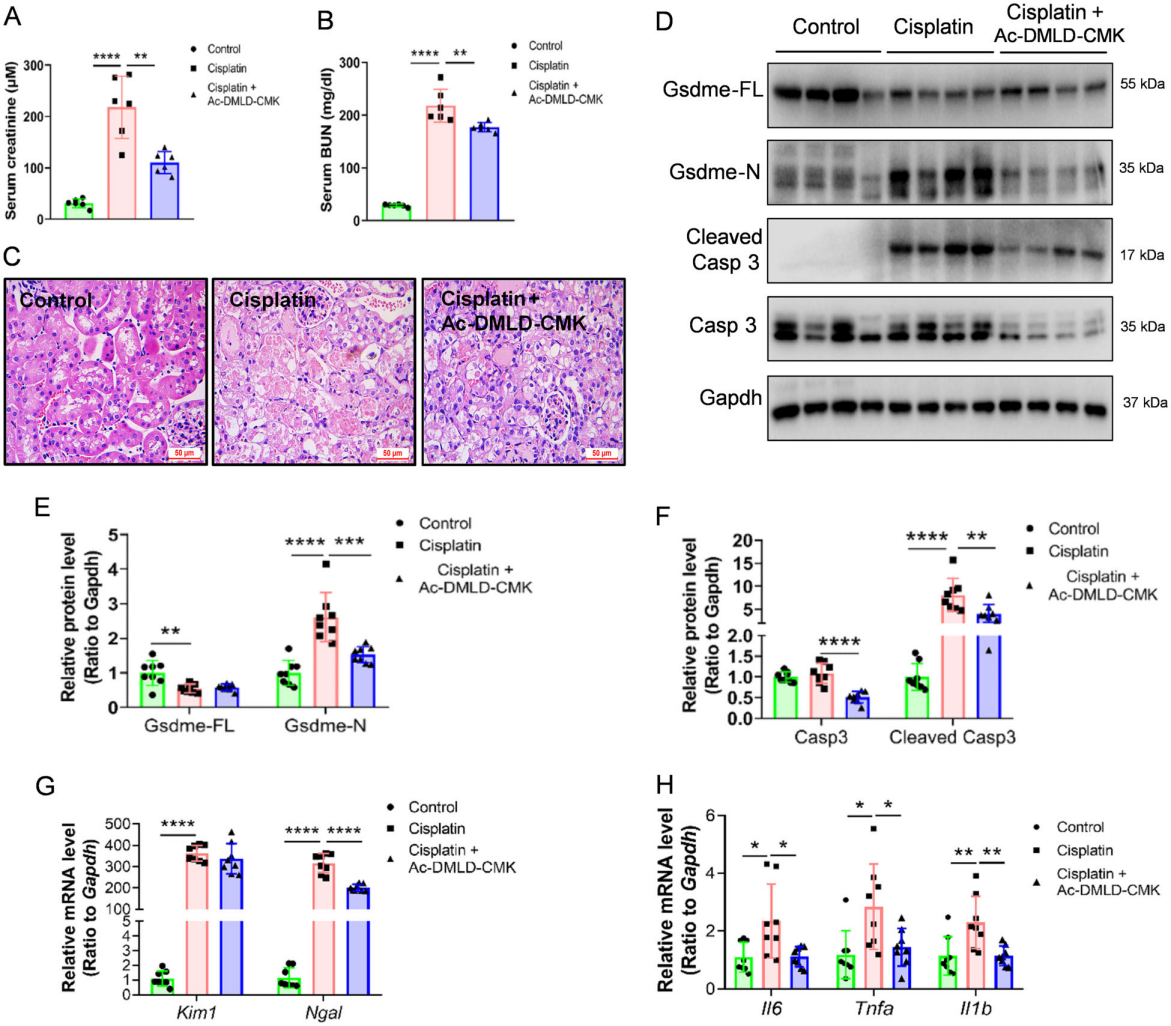
Ac-DMLD-CMK decreases cisplatin-induced renal pyroptosis in vivo. **A**, **B** Serum creatinine and BUN detected in cisplatin-treated mice before or after Ac-DMLD-CMK pre-treatment. **C** HE staining of cisplatin-treated mice before or after Ac-DMLD-CMK pre-treatment. Scale bar, 50 μm. **D**–**F** Western blot analysis of GSDME and caspase 3 in cisplatin-treated mice before, or after, Ac-DMLD-CMK pretreatment. **G**, **H** mRNA expression of kidney injury-related genes and inflammatory-related genes in cisplatin-treated mice before, or after, Ac-DMLD-CMK pretreatment. All data are shown as means ± SD from six independent experiments (*n* = 6). *p < 0.05, **p < 0.01, ***p < 0.001, ****p < 0.0001 using one-way ANOVA followed by the Tukey’s method.

Considering that the caspase 3 cleavage site in mouse GSDME is _267_DMLD_270_^14^, we next synthesized a polypeptide Ac-DMLD-CMK to inhibit caspase 3-Gsdme signaling in vivo. The Gsdme in mice-derived inhibitor Ac-DMLD-CMK decreased serum creatinine and BUN compared to cisplatin-induced mice (Fig. 5A, B). Morphologically, HE staining revealed that Ac-DMLD-CMK alleviated renal tubular epithelial cell death induced by cisplatin incubation (Fig. 5C). Moreover, western blot analysis showed that Ac-DMLD-CMK pre-treatment reduced the abundance of renal Gsdme in mice-N, cleaved caspase 3, and caspase 3, without apparent effects on Gsdme in mice-FL compared to cisplatin-treated mice (Fig. 5D–F). Furthermore, Ac-DMLD-CMK suppressed expression of the kidney injury-related gene *Ngal*, and inflammatory-related genes *Il6, Tnfa*, and *Il1b*, however, did not affect *Kim1* (Fig. 5G, H). These results imply that Ac-DMLD-CMK may protect the kidney by targeting caspase 3-Gsdme signaling in mice.

### Caspase 3-GSDME signaling is involved in doxorubicin-induced pyroptosis in human podocytes

Although the major targets of chemotherapy drug-induced nephrotoxicity are renal tubular epithelial cells, podocytes also serve as target cells of doxorubicin-induced nephrotoxicity. Hence, we also sought to detect the activation state and role of the caspase 3/GSDME/pyroptosis axis in podocytes under the doxorubicin challenge. To this end, we first compared the expression of GSDME in renal tubular epithelial cells and podocytes derived from humans or mice. Results show that GSDME had much higher expression in humans than in mice (Fig. S6a). MP and mRTEC also showed GSDME activation following cisplatin or doxorubicin induction, although with low baseline expression (Fig. S6b, c). We then stimulated human podocytes with doxorubicin and observed decreased expression of synaptopodin, suggesting that doxorubicin can induce podocyte injury (Fig. S6d). Furthermore, doxorubicin-induced GSDME and caspase 3 cleavage in a concentration-dependent manner in human podocytes (Fig. S6e, f). In addition, we observed that caspase 3-directed siRNA decreased doxorubicin-induced activation of GSDME, LDH release, and the number of pyroptotic human podocytes (Fig. S6g–m). We also knocked down GSDME using GSDME siRNA (Fig. S7a), and found increased cell viability, as well as decreased doxorubicin-induced LDH release and a reduced number of pyroptotic human podocytes, without impacting caspase 3 activation (Fig. S7b–h). Taken together, these results indicate that caspase 3-GSDME signaling also plays a vital role in doxorubicin-induced pyroptosis in human podocytes.

### ERK and JNK signaling mediate GSDME cleavage in the kidney in vitro and in vivo

**Fig. 6:**
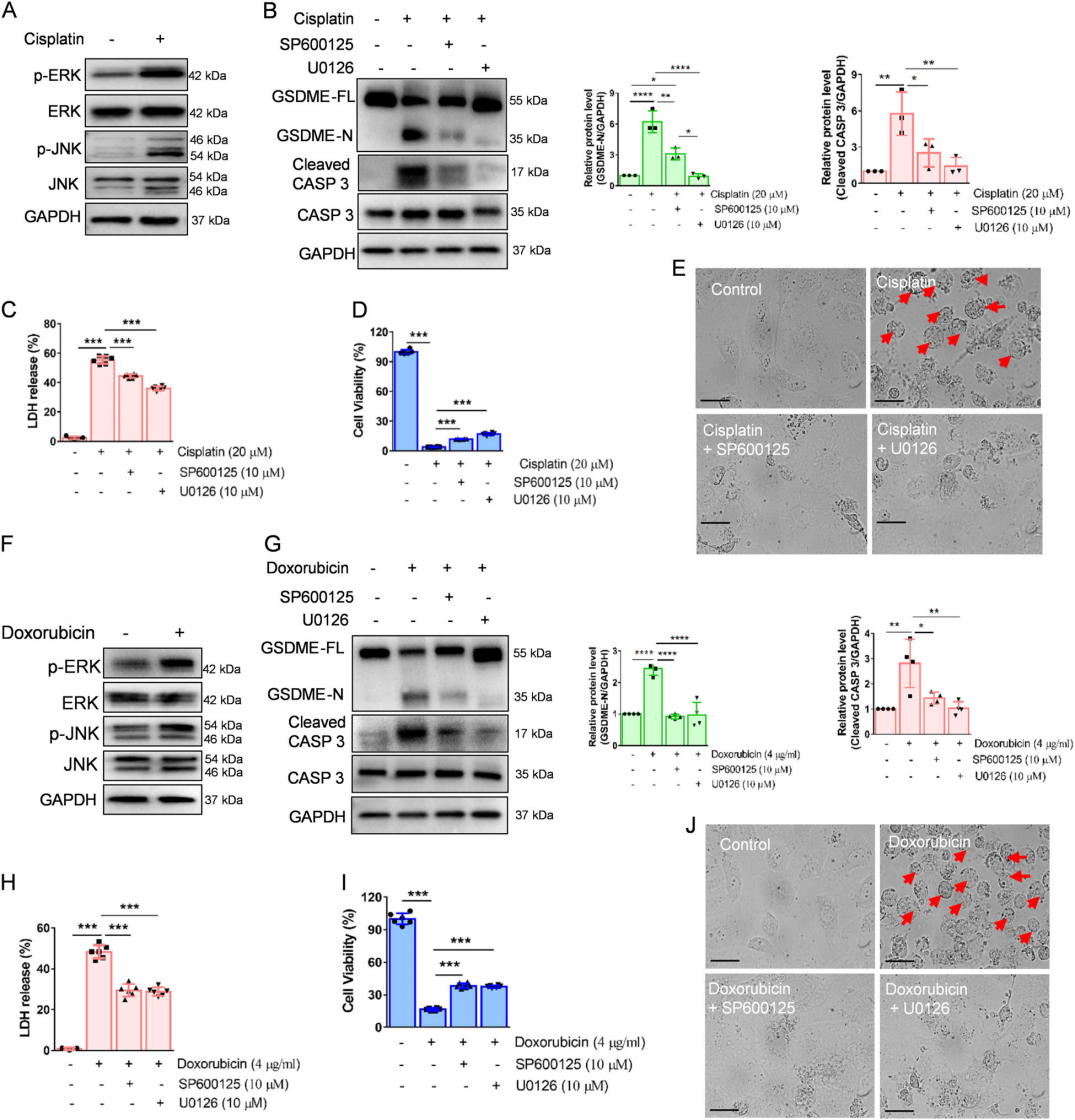
GSDME cleavage is induced by ERK and JNK signaling in HK-2 cells. **A**, **F** Western blot analysis of ERK and JNK phosphorylation in HK-2 cells incubated with cisplatin (20 μM) or doxorubicin (4 μg/ml) for 3 h. **B**, **G** Western blot analysis of the cleavage of GSDME and caspase 3 in cisplatin- (20 μM) or doxorubicin- (4 μg/ml) treated HK-2 cells for 48 h with or without pretreatment of U0126 (10 μM) and SP600125 (10 μM). Cytotoxicity and cell viability were determined using the LDH assay (**C**, **H**) and CCK-8 detection (**D**, **I**) in HK-2 cells treated with cisplatin (20 μM) or doxorubicin (4 μg/ml) for 48 h with or without pre-treatment of U0126 (10 μM) and SP600125 (10 μM). **E**, **J** Representative light microscopy images of HK-2 cells treated with cisplatin (20 μM) or doxorubicin (4 μg/ml) for 48 h with or without pre-treatment of U0126 (10 μM) and SP600125 (10 μM). The red arrow indicates bubbles emerging from the plasma membrane. Scale bar, 50 μm. All data are presented as mean ± SD from three independent experiments (*n* = 3). *p < 0.05, **p < 0.01, ***p < 0.001, ****p < 0.0001 using one-way ANOVA followed by the Tukey’s method.

**Fig. 7:**
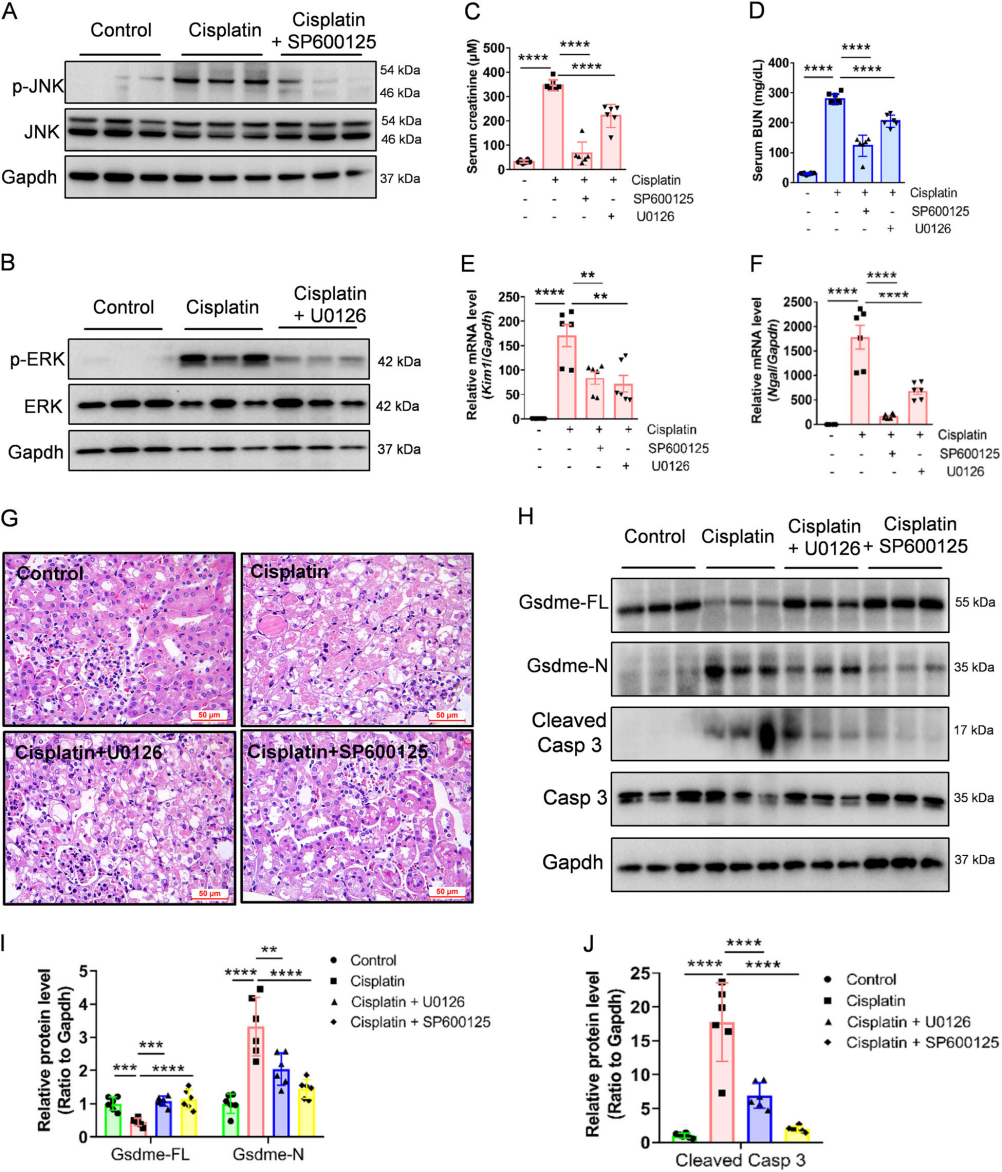
U0126 and SP600125 alleviate cisplatin-induced renal pyroptosis in vivo. **A**, **B** Western blot analysis of ERK and JNK in kidney tissues from different groups. **C**, **D** Serum creatinine and BUN detected in cisplatin-treated mice before or after U0126 and SP600125 pretreatment. **E**, **F** mRNA expression of renal injury-related genes in kidney tissues from different groups. **G** Representative images of HE staining in kidney tissues from different groups. Scale bar, 50 μm. **H**–**J** Western blot analysis of GSDME and caspase 3 in cisplatin-treated mice before, or after, Ac-DMLD-CMK pretreatment. All data are presented as mean ± SD from six independent experiments (*n* = 6). **p < 0.01, ***p < 0.001, ****p < 0.0001 using one-way ANOVA followed by the Tukey’s method.

Next, we aimed to explore the molecular mechanisms of cisplatin- or doxorubicin-induced pyroptosis in cultured HK-2 cells. ERK and JNK signaling reportedly play pivotal roles in caspase 3 activation^30,31^, which contributes to GSDME-dependent pyroptosis. Western blot analysis indicated that both ERK and JNK became phosphorylated in cisplatin- or doxorubicin-treated HK-2 cells (Fig. 6A, F). We then incubated HK-2 cells with the ERK inhibitor, U0126, or JNK inhibitor, SP600125 (Fig. S8a–d), to detect GSDME activation. Western blot results indicated that both U0126 and SP600125 inhibited GSDME cleavage and caspase 3 activation (Fig. 6B, G). In addition, U0126 and SP600125 increased HK-2 cell viability and decreased LDH release following cisplatin or doxorubicin induction (Fig. 6C, D, H, I). Furthermore, U0126- or SP600125-pretreated HK-2 cells exhibited decreased plasma membrane bubbling compared to cisplatin- or doxorubicin-treated HK-2 cells (Fig. 6E, J). Moreover, in vivo, both SP600125 (Fig. 7A) and U0126 (Fig. 7B) suppressed the increased serum creatinine and BUN induced by cisplatin (Fig. 7C, D). Meanwhile, the expression of kidney injury-related genes *Ngal* and *Kim1* decreased following SP600125 and U0126 pretreatment (Fig. 7E, F). HE staining further indicated that SP600125 and U0126 alleviated renal tubular epithelial cell death compared to cisplatin-treated mice (Fig. 7G). In addition, western blot results indicated that SP600125 and U0126 decreased cisplatin-induced cleavage of renal Gsdme in mice, and caspase 3 (Fig. 7H–J), implying that ERK and JNK signaling may act as upstream regulators of GSDME-dependent pyroptosis in renal tubular epithelial cells, both in vitro and in vivo.

### ROS induces GSDME cleavage through JNK signaling in renal tubular epithelial cells

**Fig. 8:**
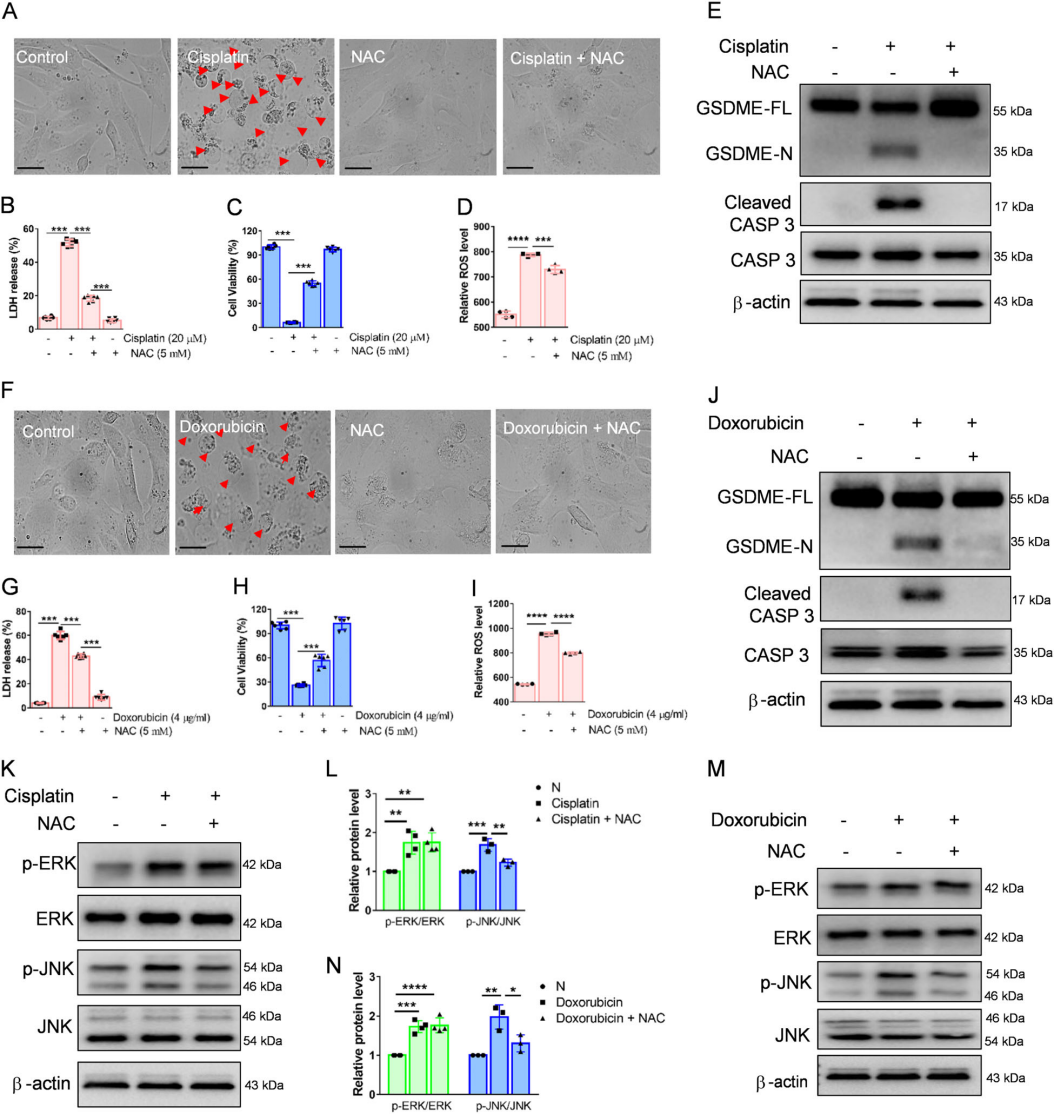
ROS induce cleavage of GSDME through JNK signaling in HK-2 cells. **A**, **F** Representative light microscopy images of HK-2 cells treated with cisplatin (20 μM) or doxorubicin (4 μg/ml) with or without pretreatment of N-acetylcysteine (NAC, 5 mM) for 48 h. The red arrow indicates bubbles emerging from the plasma membrane. Scale bar, 50 μm. Cell viability and cytotoxicity were detected using the LDH (**B**, **G**) and CCK-8 detection (**C**, **H**) in HK-2 cells. **D**, **I** The ROS level of cisplatin (20 μM) or doxorubicin (4 μg/ml) treated HK-2 cells for 48 h in the presence or absence of NAC (5 mM). **E**, **J** Western blot analysis of cleavage of GSDME and caspase 3 in cisplatin- (20 μM) or doxorubicin- (4 μg/ml) treated HK-2 cells for 48 h with or without pretreatment of NAC (5 mM). **K**–**N** Western blot analysis of ERK and JNK phosphorylation in HK-2 cells treated with cisplatin (20 μM) or doxorubicin (4 μg/ml) for 3 h in the presence or absence of NAC (5 mM). All data are presented as mean ± SD from three independent experiments (*n* = 3). *p < 0.05, **p < 0.01, ***p < 0.001, ****p < 0.0001 using one-way ANOVA followed by the Tukey’s method.

The mitochondrial apoptotic pathway, which participates in the cleavage of GSDME, can be affected by ROS^28^. Thus, we speculated that ROS are also involved in cisplatin- or doxorubicin-induced pyroptosis of HK-2 cells. Morphologically, cisplatin- or doxorubicin-induced cell bubbles were mostly inhibited by the ROS inhibitor NAC incubation (Fig. 8A, F). Furthermore, NAC increased HK-2 cell viability and decreased LDH release after cisplatin- or doxorubicin induction (Fig. 8B, C, G, H). We also found that ROS levels, augmented by cisplatin or doxorubicin, were markedly attenuated by NAC (Fig. 8D, I). In addition, NAC dramatically inhibited GSDME cleavage and caspase 3 activation, as shown by western blot (Fig. 8E, J). ROS also affects mitogen-activated protein kinase (MAPK) signaling pathways^32,33^. We found that the phosphorylation of JNK induced by cisplatin or doxorubicin was abolished following NAC treatment, while ERK phosphorylation was not affected (Fig. 8K–N). Taken together, these data indicate that ROS induces the caspase 3-GSDME via JNK signaling in HK-2 cells.

## Discussion

In the present study, we demonstrated that cisplatin- or doxorubicin-induced renal pyroptosis is dependent on the cleavage of GSDME, which becomes induced by caspase 3 activation. In addition, we revealed that caspase 3-GSDME activation is regulated by ROS-JNK signaling. Hence, this study furthers the understanding of the mechanism responsible for chemotherapeutic drug-induced nephrotoxicity.

Earlier studies demonstrated that apoptosis and necrosis are the primary types of cell death associated with chemotherapeutic drug-induced AKI^2,34,35^. However, we observed characteristic large bubbles emerging from the plasma membrane after long exposure to cisplatin or doxorubicin in human HK-2 cells, implying the emergence of pyroptosis. Moreover, GSDME became activated in a concentration- and time-dependent manner following treatment with chemotherapy drugs, implying that chemotherapeutic drug-induced pyroptosis of HK-2 cells is GSDME-dependent.

Pyroptosis can be induced by caspase 4 and 5 (in humans) or caspase 11 (in mice) activation via GSDMD cleavage, leading to cell bubbling and the release of IL-1β^21,36–38^. Recently discovered caspase 3/GSDME signaling was also reported to be one of the signaling mechanisms for cell pyroptosis^14,28,39–41^. Moreover, GSDME-positive cancer cells reportedly undergo pyroptosis, while GSDME-negative cancer cells undergo apoptosis upon stimulation with chemotherapy drugs^14,27^. Thus, GSDME expression might determine the type of cell death that results from chemotherapeutic drug exposure. In this study, we showed that similar to other GSDME-positive cells, caspase 3 inhibition with siRNA or the specific inhibitor Z-DEVD-FMK prevented GSDME activation and subsequent pyroptosis in HK-2 cells, implying that caspase 3 is involved in HK-2 cell pyroptosis via GSDME cleavage. However, caspase 9 siRNA showed no effect on the activation of GSDME in HK-2 cells, which differed from the results of Zhou et al.^28^ in colon cancer cells and Tsuchiya et al.^42^ in macrophages. This discrepancy might be caused by cell-type differences. Moreover, caspase 3 inhibition has been reported to reflexively activate caspase 7^14^. Herein, we found that caspase 7 knockdown activated caspase 3, resulting in increased cleavage of GSDME. Taken together, these results indicate that GSDME is recognized by caspase 3 in HK-2 cells.

Previous reports have indicated that GSDMD is the main executor of pyroptosis in chemotherapy drug-induced AKI^22,43^, in which they observed that cisplatin cleaved renal GSDMD by upregulating the expression of caspase 11, which subsequently initiates cell pyroptosis. Furthermore, Miao et al.^43^ showed that GSDMD deficiency alleviates cisplatin-induced renal morphological changes, and renal function deterioration, as well as urinary IL-18 release. Our results demonstrated that GSDME also functions as a critical target of chemotherapy drug-induced AKI. Similar to that of other reports^40,44,45^, our in vitro results indicated that GSDME knockout alleviated renal tubular epithelial cell pyroptosis. In vivo, the Gsdme-derived inhibitor, Ac-DMLD-CMK, alleviated deterioration of kidney function, attenuated renal tubular epithelial cell injury, reduced inflammatory cytokine secretion, and inhibited caspase 3-GSDME signaling induced by cisplatin. Similarly, a previous study reported that GSDMEb knockout- or GSDMEb-derived inhibitor Ac-FEID-CMK-treated zebrafish exhibited reduced proximal renal tubule structure injury compared to the control, indicating that GSDMEb plays an essential role in proximal tubular cell pyroptosis-mediated AKI in zebrafish^46^. In fact, in addition to the kidney, GSDME also plays an important role in other organs and diseases, however, most of the current studies on GSDME focus on tumors^14^. For instance, *GSDME* has been reported to function as a tumor suppressor gene by directly inducing tumor cell pyroptosis through caspase 3, as well as indirectly by acting on T lymphocytes through Granzyme B^14,47^. Furthermore, one study showed that GSDME amplified the apoptotic pathway by creating holes in the mitochondria membrane, leading to the release of cytochrome c^48^. Taken together, these results demonstrate the complexity of the mechanism associated with GSDME cleavage. Hence, further investigation is required to clarify the precise mechanism responsible for GSDME regulation.

The MAPK signaling pathway plays an essential role in renal tubular epithelial cell proliferation, survival, and differentiation. Jo et al.^31^ indicated that the ERK inhibitor U1026 alleviates cisplatin-induced kidney injury and attenuated necrosis of tubular cells by reducing cisplatin-induced caspase 3 cleavage. Similarly, the JNK inhibitor SP600125 also reportedly alleviates cisplatin-induced renal injury^49^. In addition, Yu et al.^30^ reported that JNK is involved in lobaplatin-induced colon cancer cell pyroptosis by activating the caspase 3/GSDME signaling pathway. In our in vitro study, we found that both ERK and JNK were activated following cisplatin or doxorubicin treatment, while inhibitors targeting ERK and JNK alleviated cisplatin- or doxorubicin-induced HK-2 cell pyroptosis via inhibition of caspase 3 and GSDME activation. Meanwhile, our in vivo study demonstrated a protective effect for U0126 and SP600125 on decreased kidney function as well as GSDME, and caspase 3 activation in the kidney. Note, SP600125, elicited a stronger protective effect, indicating that both JNK and ERK, particularly JNK, are involved in renal tubular epithelial cell pyroptosis.

The p38 signaling was also reported to participate in cisplatin-induced nephrotoxicity^50^. Thus, we assessed the effect of p38 on cell pyroptosis using the p38 inhibitors, SB203580 and SB202190, however, no protective effect was observed in HK-2 cells (data not shown), suggesting that p38 is not involved in HK-2 cell pyroptosis. However, Ramesh et al.^50^ found that p38 MAP kinase inhibition alleviated cisplatin-induced nephrotoxicity in mice. The discrepancy between these results requires further investigation.

As common chemotherapy drugs, cisplatin and doxorubicin have been reported to induce ROS production^11^. Indeed, ROS generation is believed to be one of the major mechanisms of chemotherapeutic drug-induced nephrotoxicity. Excessive ROS causes cell death by activating the MAPK signaling pathway. Zhou et al.^28^ found that ROS elevation stimulates caspase 3/GSDME-dependent pyroptosis in iron-treated cancer cells. Similarly, we demonstrated that NAC, a ROS inhibitor, significantly alleviates cisplatin- or doxorubicin-induced ROS and cell pyroptosis in HK-2 cells. Furthermore, NAC inhibited JNK phosphorylation without a noticeable effect on ERK activation, suggesting that ROS is an upstream regulator of JNK in HK-2 cells. Another study also demonstrated that NAC attenuates lobaplatin-induced colon cancer cell pyroptosis by regulating JNK phosphorylation^30^. It was reported that phosphorylated JNK can recruit Bax to mitochondria, prompting cytochrome c release into the cytosol, and subsequently activating caspase 3 and pyroptosis^30^. Considering these data, we conclude that chemotherapy drug-induced pyroptosis in renal tubular epithelial cells is regulated via the ROS/JNK/caspase 3/GSDME signaling pathway.

Pyroptosis was reported to be important for the antitumor activity of chemotherapy drugs. More recently, many studies have indicated that reagents or drugs targeting GSDME showed an antitumor effect. For instance, Miltirone, derived from a traditional herb *Salvia miltiorrhiza*, was shown to possess antitumor activity by inducing GSDME activation in hepatocellular carcinoma^51^. Furthermore, a PLK1 kinase inhibitor was reported to improve the effect of cisplatin in the treatment of esophageal squamous cell carcinoma by inducing pyroptosis^52^. Diverse small-molecule inhibitors have also been shown to augment the anti-cancer effect by inducing GSDME cleavage. However, our results showed that the chemotherapy drugs cisplatin or doxorubicin induce GSDME activation, leading to the pyroptosis of normal renal tubular epithelial cells, implying that GSDME-targeted anticancer therapy would worsen renal pathology. Most of the healthy organs were GSDME-positive^14^. Similarly, Xu et al.^53^ demonstrated that GSDME cleavage is involved in acute hepatic failure. Thus, to protect healthy organs from chemotherapeutic drug toxicity by inhibiting GSDME activation could adversely affect the antitumor effect of chemotherapy drugs. Hence, the toxicity and associated adverse side effects of chemotherapy drugs on normal organs should be carefully considered when designing antitumor therapies targeting GSDME.

In conclusion, we have found that the chemotherapy drugs cisplatin or doxorubicin induce pyroptosis of human renal tubular epithelial cells via ROS/JNK/caspase 3/GSDME signaling. Therapies targeting GSDME could be effective in attenuating chemotherapy drug-induced nephrotoxicity. This study may advance the understanding of this process.

## Materials and methods

### Cell culture and treatments

The human proximal tubular epithelial cell line, HK-2, was bought from the American Type Culture Collection (ATCC, Manassas, VA, USA). Briefly, HK-2 cells were cultured in DMEM/F12 medium (Cat# D8437, Sigma, MO, USA) supplemented with 10% fetal bovine serum (FBS) (Cat# 10091-148, Gibco, CA, USA), streptomycin (100 mg/ml), and penicillin (100 U/ml) at 37 °C with 5% CO_2_. Human podocytes (HP) were donated by professor Youying Mao at Shanghai Children’s Medical Center. HP was cultured in RPMI 1640 with 10% FBS and ITS (1:100, Cat# 41400045, Gibco, CA, USA) with 5% CO_2_ at 33 °C, after which HP was transferred to 37 °C for differentiation. To induce cell pyroptosis, the HK-2 cells and HP were incubated in a serum-free medium overnight and treated with cisplatin (Cat# S1166, Selleck, MA, USA) or doxorubicin (Cat# D1515, Sigma). For the intervention experiment, cells were preincubated with 50 μM of Z-VAD-FMK (Cat# ALX-260-020, ENZO, NY, USA), 100 μM of Z-DEVD-FMK (Cat# FMK004, R&D, MN, USA), 10 μM of GSK872 (Cat# S8465, Selleck), 10 μM of U0126 (Cat# 9903, Cell Signaling Technology, MA, USA), 10 μM of SP600125 (Cat# S1460, Selleck), or 5 mM of N-acetylcysteine (NAC, Cat# S0077, Beyotime, Shanghai, China) for 1 h before cisplatin or doxorubicin treatment.

### Small interfering RNA (siRNA) and shRNA (short-hairpin RNA) knockdown

The siRNAs for caspase 3, caspase 9, and GSDME were purchased from the GenePharma Company and transfected into HK-2 cells. Briefly, HK-2 cells and HP were seeded in 6-well plates and transfected with scrambled, caspase 3, caspase 9, or GSDME siRNA using Lipofectamine RNAiMAX (Life Technologies, CA, USA) transfection reagent according to the manufacturer’s protocols. The HK-2 cells were incubated for 48 h at 37 °C with 5% CO_2_. The efficacy of the siRNA knockdown was determined using western blot analysis. The sequences of the siRNAs used in the experiments were shown in Table S1.

For shRNA knockdown, the HK-2 cells were seeded in 24-well plates and transfected with control constructs or caspase 7 shRNA (Genechem, Shanghai, China) using Lipofectamine 3000 (Life Technologies, CA, USA) according to the manufacturer’s instructions. After 48 h, the transfected HK-2 cells were selected by their puromycin resistance. The efficacy of the caspase 7 shRNA was determined by western blot analysis.

### CRISPR–Cas 9 knockouts of GSDME

HK-2 cells were seeded in a 24-well plate at 1 × 10^4^ cells/well and transfected with the px459 empty plasmid and px459-GSDME-KO plasmid (YouBio, Changsha, Hunan, China) using Lipofectamine 3000 (Life Technologies, CA, USA) for 48 h. Then, the transfected HK-2 cells were selected by puromycin treatment. The specificity of GSDME-KO was determined by western blot analysis.

### Ethics statement

Normal human renal biopsy specimens were collected from healthy donors for kidney transplantation, which was approved by the Ethics Committee of the First Affiliated Hospital, Zhejiang University, School of Medicine (2020-607). The relevant experiments were conducted following approved guidelines of the First Affiliated Hospital, Zhejiang University, School of Medicine.

### Animal experiment

Adult male C57BL/6 mice (Shanghai Laboratory Animal Center, Shanghai, China) weighing 20–25 g at about 6–8 weeks were used in this experiment. The procedures were approved by the Guidelines for Animal Care and Use of Laboratory Animals from the First Affiliated Hospital, College of Medicine, Zhejiang University (2020-1541). The mice were randomly divided into five groups: for the AKI injury model, the mice were administered a single intraperitoneal injection of cisplatin (25 mg/kg body weight). For the intervention groups, U0126 (10 mg/kg, in 5% DMSO + 30% PEG300 + 5% Tween 80; Cat#S1102, Selleck), SP600125 (10 mg/kg, in 5% DMSO + 30% PEG300 + 5% Tween 80; Cat# S1460, Selleck) or Ac-DMLD-CMK (5 mg/kg/day, in 0.9% saline; Chinese Peptide Company, Hangzhou, China) were administered via intraperitoneal injection 1 h before cisplatin administration. The control mice were administered the same volume of 5% DMSO + 30% PEG300 + 5%Tween 80 or 0.9% saline. After 72 h, the mice were anesthetized with pentobarbital sodium. Serum creatinine and blood urea nitrogen (BUN) were detected using the FUJIDRI-CHEM 7000i biochemistry analyzer (FUJIFILM, Tokyo, Japan). Mouse kidneys were collected for western blot detection and HE staining.

### Cell cytotoxicity and viability assays

Cells were seeded into a 96-well plate (10,000 cells/well in 200 μl medium) and treated with related reagents for 48 h. Next, cell cytotoxicity and viability were assessed using a kit (Cat# CK17, Dojindo, Tokyo, Japan) according to the manufacturer’s instructions. The absorbance was measured using a Microplate Reader (Infinite^®^ M1000, TECAN, Switzerland).

### Flow cytometric analysis

For flow cytometry detection, each group of HK-2 cells was treated and collected after trypsin digestion. The HK-2 cell suspension was washed with cold phosphate-based buffer, resuspended, and labeled with Annexin V-FITC and PI according to the manufacturer’s protocol (Cat# 556547, Becton Dickinson, NJ, USA). Apoptosis was analyzed by flow cytometry (BD). PI-positive cells were considered pyroptotic cells.

### Reactive oxygen species (ROS) measurement

The ROS levels in HK-2 cells were detected with the DCFH-DA Detection Kit (S0033, Beyotime, Shanghai, China). Briefly, the HK-2 cells were seeded in a 6-well plate and incubated with cisplatin or doxorubicin in the presence or absence of NAC for 48 h. After washing, the cells were stained with 10 μM of DCFH-DA at 37 °C for 30 min according to the manufacturer’s instructions.

### Immunohistochemistry

Fixed, paraffin-embedded human renal biopsy specimens (1.5-μm thick) were deparaffinized, rehydrated, and blocked with 1.5% H_2_O_2_–methanol. After washing with phosphate-buffered saline (PBS), the slides were subjected to antigen retrieval in citrate buffer. Non-specific binding was blocked with 10% donkey serum for 30 min. After that, the slides were incubated with a rabbit anti-GSDME antibody (1:100, Cat# ab215191, Abcam, MA, USA) overnight at 4 °C. Then, the donkey anti-rabbit/mouse antibody was incubated for 30 min and washed with PBS. After staining with 3,3′-diaminobenzidine (DAB), the slides were counterstained with hematoxylin and examined under a microscope (Leica DMLB, Wetzlar, Germany).

### qRT-PCR

Total RNA extraction of the mouse kidney cortex was performed with Trizol reagent (Cat# 15596018, Invitrogen, CA, USA). The Prime-Script RT reagent kit (Cat# RR047B, Takara Biotechnology, Dalian, China) was then used to reverse transcribe the RNA to cDNA. RT-PCR was performed with the SYBR Green Mix (Cat# Q711-02/03, Vazyme, Nanjing, China) on the ViiA7 Real-Time PCR system (Applied Biosystems, CA, USA). Primer sequences used were listed in Table S2.

### Western blot analysis

Cells were lysed in the denaturing buffer of the Total Protein Extraction Kit (Cat# SD-001, Invent Biotechnologies, Beijing, China) to obtain protein extracts. Then, 20 μg of total protein from each group were subjected to SDS-PAGE gels and transferred to PVDF membranes (Millipore, Billerica, MA, USA). The membranes were incubated with primary antibodies targeting GSDME (Cat# ab215191, Abcam), caspase 3 (Cat# 14220, CST), caspase 6 (Cat# 9762, CST), caspase 7 (Cat# 12827, CST), caspase 8 (Cat# 9746, CST), caspase 9 (Cat# 9502, CST), PARP (Cat# 9532, CST), Bax (Cat# 5023, CST), Bcl-XL (Cat# 2764, CST), JNK (Cat# 9258, CST), p-JNK (Cat# 4668, CST), ERK (Cat# 4695, CST), p-ERK (Cat# 4370, CST), β-actin (Cat# sc-47778, Santa Cruz) and GAPDH (Cat# BK7021, Bioke, Hangzhou, China). The membranes were then washed with TBST and incubated with horseradish peroxidase-conjugated secondary antibodies. The bands were then visualized by the ECL Chemiluminescence Kit (Cat# abs920, Absin Biotechnologies, Shanghai, China) and captured by the enhanced chemiluminescence detection system ChemiDoc MP (Bio-RAD, CA, USA).

### Statistical analysis

Each experiment is repeated at least three times independently. Data are presented as means ± standard deviations. Quantitative results were analyzed using GraphPad Prism 7 (GraphPad Software Inc., San Diego, CA, USA). Comparisons between two groups were made using two-tailed Student’s *t* tests. Data from multiple groups were compared using one-way ANOVA followed by the Tukey’s post hoc method. *p* < 0.05 was considered to indicate statistical significance.

## Supplementary information

Supplement figure

Supplement table

Supplement material-original western blot pictures

## Data Availability

The datasets used during the current study are available from the corresponding author on reasonable request.

## References

[1] Pabla N, Dong Z (2008). Cisplatin nephrotoxicity: mechanisms and renoprotective strategies. Kidney Int..

[2] Ozkok A, Edelstein CL (2014). Pathophysiology of cisplatin-induced acute kidney injury. Biomed. Res. Int..

[3] Gabizon AA, Patil Y, La-Beck NM (2016). New insights and evolving role of pegylated liposomal doxorubicin in cancer therapy. Drug Resist. Update.

[4] Broxterman HJ, Gotink KJ, Verheul HM (2009). Understanding the causes of multidrug resistance in cancer: a comparison of doxorubicin and sunitinib. Drug Resist. Update.

[5] Izzedine H (2018). Drug nephrotoxicity. Nephrol. Ther..

[6] Volarevic V (2019). Molecular mechanisms of cisplatin-induced nephrotoxicity: a balance on the knife edge between renoprotection and tumor toxicity. J. Biomed. Sci..

[7] Shiraishi F (2000). Heme oxygenase-1 gene ablation or expression modulates cisplatin-induced renal tubular apoptosis. Am. J. Physiol. Ren. Physiol..

[8] Chen S (2019). Tenascin-C protects against acute kidney injury by recruiting Wnt ligands. Kidney Int..

[9] Spath MR (2019). The proteome microenvironment determines the protective effect of preconditioning in cisplatin-induced acute kidney injury. Kidney Int.

[10] Canaud G (2019). Cyclin G1 and TASCC regulate kidney epithelial cell G2-M arrest and fibrotic maladaptive repair. Sci. Transl. Med..

[11] Zuk A, Bonventre JV (2016). Acute kidney injury. Annu. Rev. Med..

[12] Zhang Y, Chen X, Gueydan C, Han J (2018). Plasma membrane changes during programmed cell deaths. Cell Res..

[13] Shi J (2015). Cleavage of GSDMD by inflammatory caspases determines pyroptotic cell death. Nature.

[14] Wang Y (2017). Chemotherapy drugs induce pyroptosis through caspase-3 cleavage of a gasdermin. Nature.

[15] Feng S, Fox D, Man SM (2018). Mechanisms of gasdermin family members in inflammasome signaling and cell death. J. Mol. Biol..

[16] Liu Y (2019). Visualization of perforin/gasdermin/complement-formed pores in real cell membranes using atomic force microscopy. Cell Mol. Immunol..

[17] Fricker M, Tolkovsky AM, Borutaite V, Coleman M, Brown GC (2018). Neuronal cell death. Physiol. Rev..

[18] Muendlein HI (2020). cFLIPL protects macrophages from LPS-induced pyroptosis via inhibition of complex II formation. Science.

[19] Johnson DC (2018). DPP8/DPP9 inhibitor-induced pyroptosis for treatment of acute myeloid leukemia. Nat. Med..

[20] Orning P (2018). Pathogen blockade of TAK1 triggers caspase-8-dependent cleavage of gasdermin D and cell death. Science.

[21] Zhang Z (2018). Caspase-11-mediated tubular epithelial pyroptosis underlies contrast-induced acute kidney injury. Cell Death Dis..

[22] Li Y (2020). Activation of GSDMD contributes to acute kidney injury induced by cisplatin. Am. J. Physiol. Ren. Physiol..

[23] Wang Y (2019). TLR4/NF-kappaB signaling induces GSDMD-related pyroptosis in tubular cells in diabetic kidney disease. Front. Endocrinol..

[24] Wu H (2016). MiR-155 is involved in renal ischemia-reperfusion injury via direct targeting of FoxO3a and regulating renal tubular cell pyroptosis. Cell Physiol. Biochem..

[25] Erkes DA (2020). Mutant BRAF and MEK inhibitors regulate the tumor immune microenvironment via pyroptosis. Cancer Discov..

[26] Xia X (2019). The role of pyroptosis in cancer: pro-cancer or pro-“host”?. Cell Death Dis..

[27] Lu H (2018). Molecular targeted therapies elicit concurrent apoptotic and GSDME-dependent pyroptotic tumor cell death. Clin. Cancer Res..

[28] Zhou B (2018). Tom20 senses iron-activated ROS signaling to promote melanoma cell pyroptosis. Cell Res..

[29] Wallach D, Kang TB, Dillon CP, Green DR (2016). Programmed necrosis in inflammation: toward identification of the effector molecules. Science.

[30] Yu J (2019). Cleavage of GSDME by caspase-3 determines lobaplatin-induced pyroptosis in colon cancer cells. Cell Death Dis..

[31] Jo SK, Cho WY, Sung SA, Kim HK, Won NH (2005). MEK inhibitor, U0126, attenuates cisplatin-induced renal injury by decreasing inflammation and apoptosis. Kidney Int..

[32] Deng Z (2019). M1 macrophage mediated increased reactive oxygen species (ROS) influence wound healing via the MAPK signaling in vitro and in vivo. Toxicol. Appl. Pharm..

[33] Pereira L, Igea A, Canovas B, Dolado I, Nebreda AR (2013). Inhibition of p38 MAPK sensitizes tumour cells to cisplatin-induced apoptosis mediated by reactive oxygen species and JNK. EMBO Mol. Med..

[34] Sanchez-Gonzalez PD, Lopez-Hernandez FJ, Lopez-Novoa JM, Morales AI (2011). An integrative view of the pathophysiological events leading to cisplatin nephrotoxicity. Crit. Rev. Toxicol..

[35] Linkermann A (2014). Regulated cell death in AKI. J. Am. Soc. Nephrol..

[36] Wang K (2020). Structural mechanism for GSDMD targeting by autoprocessed caspases in pyroptosis. Cell.

[37] Zaslona Z (2020). Caspase-11 promotes allergic airway inflammation. Nat. Commun..

[38] Kesavardhana S, Malireddi RKS, Kanneganti TD (2020). Caspases in cell death, inflammation, and pyroptosis. Annu. Rev. Immunol..

[39] Rogers C (2017). Cleavage of DFNA5 by caspase-3 during apoptosis mediates progression to secondary necrotic/pyroptotic cell death. Nat. Commun..

[40] Zeng CY (2019). ATP induces caspase-3/gasdermin E-mediated pyroptosis in NLRP3 pathway-blocked murine macrophages. Apoptosis.

[41] Mai FY (2019). Caspase-3-mediated GSDME activation contributes to cisplatin- and doxorubicin-induced secondary necrosis in mouse macrophages. Cell Prolif..

[42] Tsuchiya K (2019). Caspase-1 initiates apoptosis in the absence of gasdermin D. Nat. Commun..

[43] Miao N (2019). The cleavage of gasdermin D by caspase-11 promotes tubular epithelial cell pyroptosis and urinary IL-18 excretion in acute kidney injury. Kidney Int..

[44] Zhang CC (2019). Chemotherapeutic paclitaxel and cisplatin differentially induce pyroptosis in A549 lung cancer cells via caspase-3/GSDME activation. Apoptosis.

[45] Wang Y (2018). GSDME mediates caspase-3-dependent pyroptosis in gastric cancer. Biochem. Biophys. Res. Commun..

[46] Wang Z (2020). Zebrafish GSDMEb cleavage-gated pyroptosis drives septic acute kidney injury in vivo. J. Immunol..

[47] Zhang Z (2020). Gasdermin E suppresses tumour growth by activating anti-tumour immunity. Nature.

[48] Rogers C (2019). Gasdermin pores permeabilize mitochondria to augment caspase-3 activation during apoptosis and inflammasome activation. Nat. Commun..

[49] Francescato HD, Costa RS, Junior FB, Coimbra TM (2007). Effect of JNK inhibition on cisplatin-induced renal damage. Nephrol. Dial. Transpl..

[50] Ramesh G, Reeves WB (2005). p38 MAP kinase inhibition ameliorates cisplatin nephrotoxicity in mice. Am. J. Physiol. Ren. Physiol..

[51] Zhang X (2020). Miltirone induces cell death in hepatocellular carcinoma cell through GSDME-dependent pyroptosis. Acta. Pharm. Sin. B..

[52] Wu M (2019). A PLK1 kinase inhibitor enhances the chemosensitivity of cisplatin by inducing pyroptosis in oesophageal squamous cell carcinoma. EBioMedicine.

[53] Xu W (2021). Gasdermin E-derived caspase-3 inhibitors effectively protect mice from acute hepatic failure. Acta Pharmacol. Sin.

