# Supplementary material for: Caspase 3/GSDME-dependent pyroptosis contributes to chemotherapy drug-induced nephrotoxicity
Source: Cell Death Dis. 2021 Feb 15;12(2):186. doi: 10.1038/s41419-021-03458-5 (PMC7884686; doi:10.1038/s41419-021-03458-5)
Supplement: Supplementary file 1 — Supplement figure [file 41419_2021_3458_MOESM1_ESM.docx]

**Caspase 3/GSDME-dependent pyroptosis contributes to chemotherapy drug-induced nephrotoxicity**

**Running title:** Pyroptosis in chemotherapeutic nephrotoxicity

Xiujin Shen^1*^. Haibing Wang^2*^. Chunhua Weng^1^. Hong Jiang^1^. Jianghua Chen^1*^

^1^Kidney Disease Center, the First Affiliated Hospital, College of Medicine, Zhejiang University; Key Laboratory of Kidney Disease Prevention and Control Technology, Zhejiang Province; National Key Clinical Department of Kidney Diseases; Institute of Nephrology, Zhejiang University; the Third Grade Laboratory under the National State, Administration of Traditional Chinese Medicine, Hangzhou, China

^2^ Central Laboratory, the First Affiliated Hospital of Zhejiang Chinese Medical University, Hangzhou, China.

*** Correspondence**

Xiujin Shen

E-mail: xiujinshen@zju.edu.cn.

Haibing Wang

E-mail: [whb2016@zcmu.edu.cn](mailto:whb2016@zcmu.edu.cn).

Jianghua Chen

E-mail: [chenjianghua@zju.edu.cn](mailto:chenjianghua@zju.edu.cn).


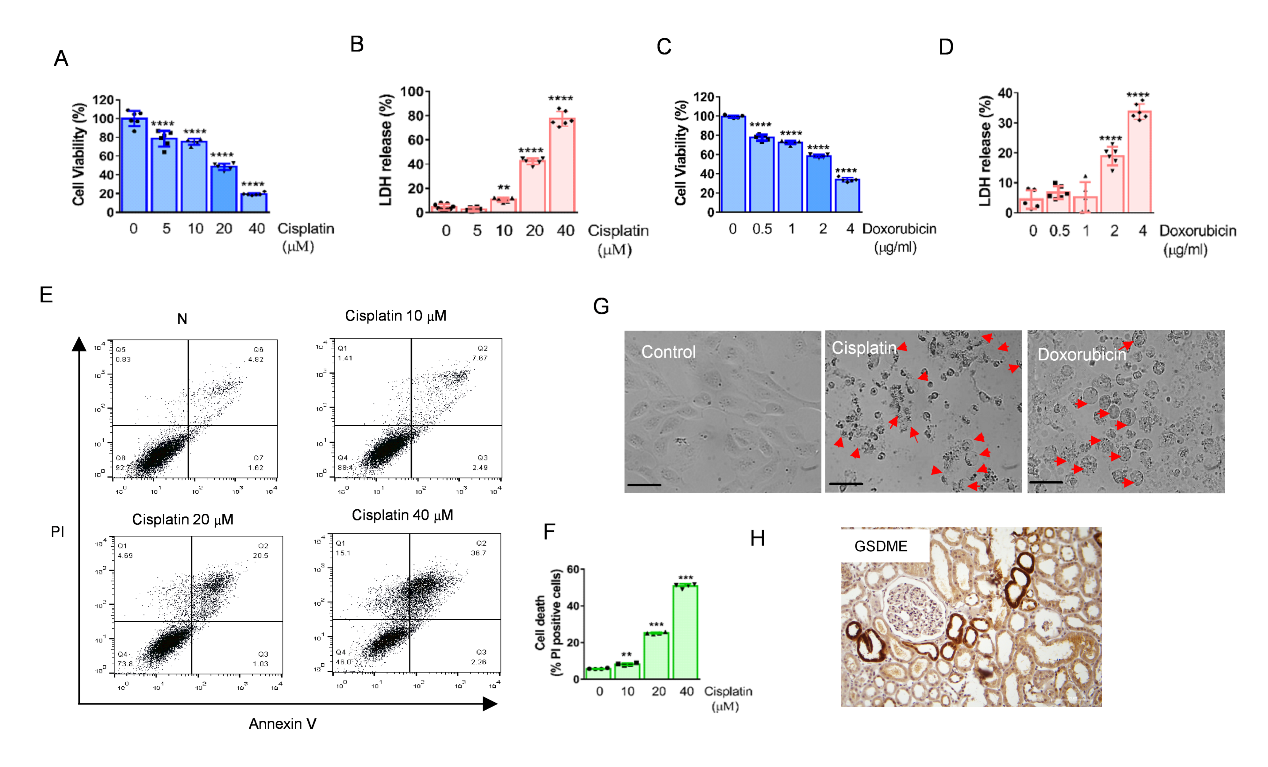


**Figure S1. Cisplatin and doxorubicin inhibit cell viability and induce pyroptosis in HK-2 cells**. HK-2 cells were treated with elevated concentrations of cisplatin (0, 5, 10, 20, and 40 μM) or doxorubicin (doxorubicin, 0, 0.5, 1, 2, and 4 μg/ml) for 48 h. Cell viability and cytotoxicity were determined using the CCK-8 assay (**a, c**) and LDH detection (**b, d**). **e, f** Flow cytometry analysis and the quantitative results indicated the percentage of propidium iodide positive (PI^+^) HK-2 cells before and after cisplatin or doxorubicin treatment. **g** Representative light microscopy images of HK-2 cells treated with cisplatin and doxorubicin. A red arrow indicates bubbles emerging from the plasma membrane. Scale bar, 100 μm. **h** IHC staining of GSDME in normal human renal biopsy specimens. Scale bar, 100 μm. All data are shown as means ± SD from three independent experiments (*n* = 3). ** *p* < 0.01 versus control group, *** *p* < 0.001 versus control group, **** *p* < 0.0001 versus control group using one-way ANOVA followed by Tukey's method.


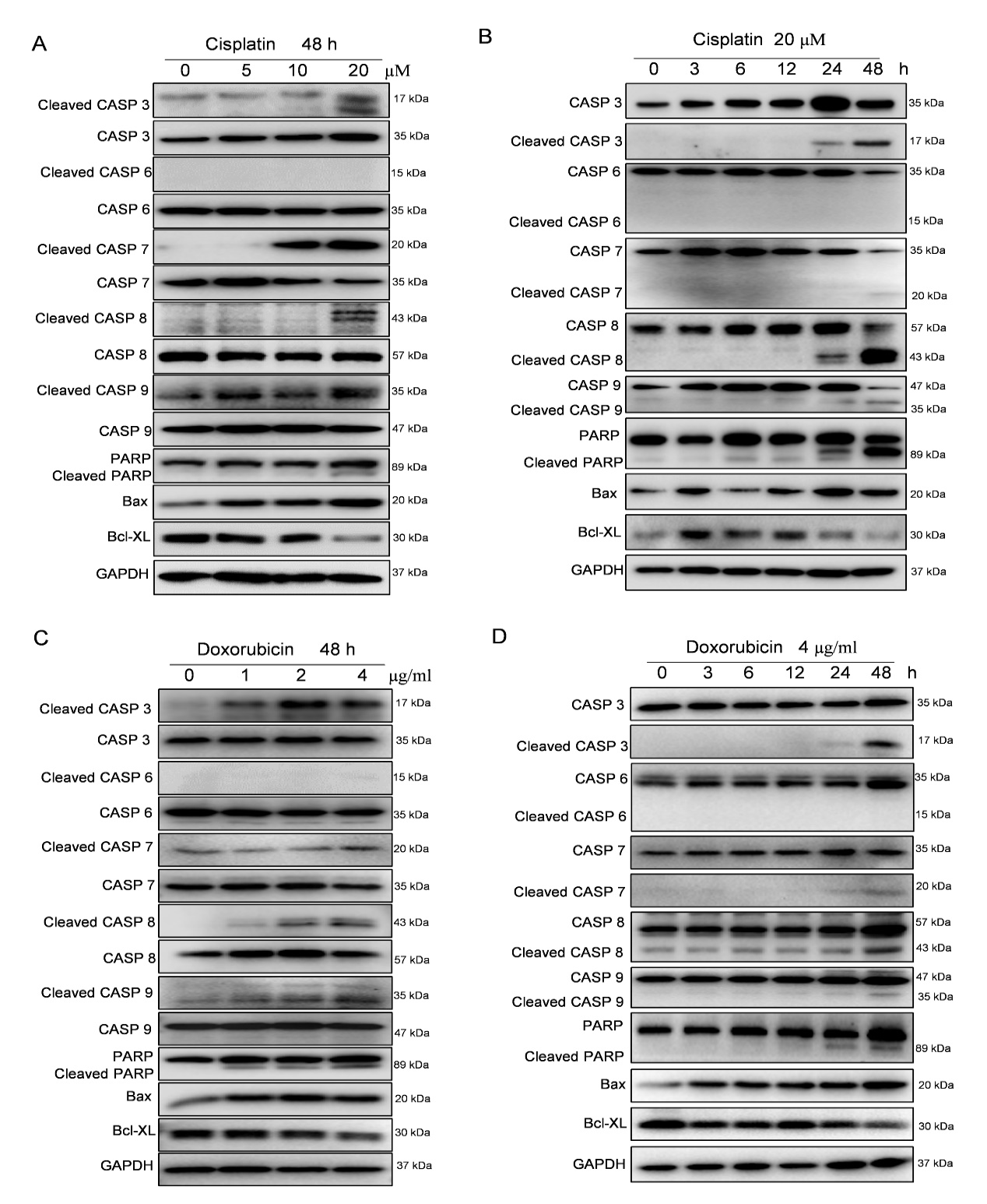


**Figure S2. The expression of apoptosis-related proteins in cisplatin and doxorubicin-induced HK-2 cells. a** Western blot analysis of the expression of apoptosis-related proteins in HK-2 cells treated with different concentrations of cisplatin (0, 5, 10, 20, and 40 μM) for 48 hours. **b** Western blot analysis of the expression of apoptosis-related proteins in HK-2 cells treated at a different time of cisplatin (0, 3, 6, 12, 24, and 48 hours) at a concentration of 20 μM. **c** Western blot analysis of the expression of apoptosis-related proteins in HK-2 cells treated with different concentrations of doxorubicin (0, 0.5, 1, 2, and 4 μg/ml)for 48 hours. **d** Western blot analysis of the expression of apoptosis-related proteins in HK-2 cells treated at a different time of doxorubicin (0, 3, 6, 12, 24, and 48 hours) at a concentration of 4 μg/ml.


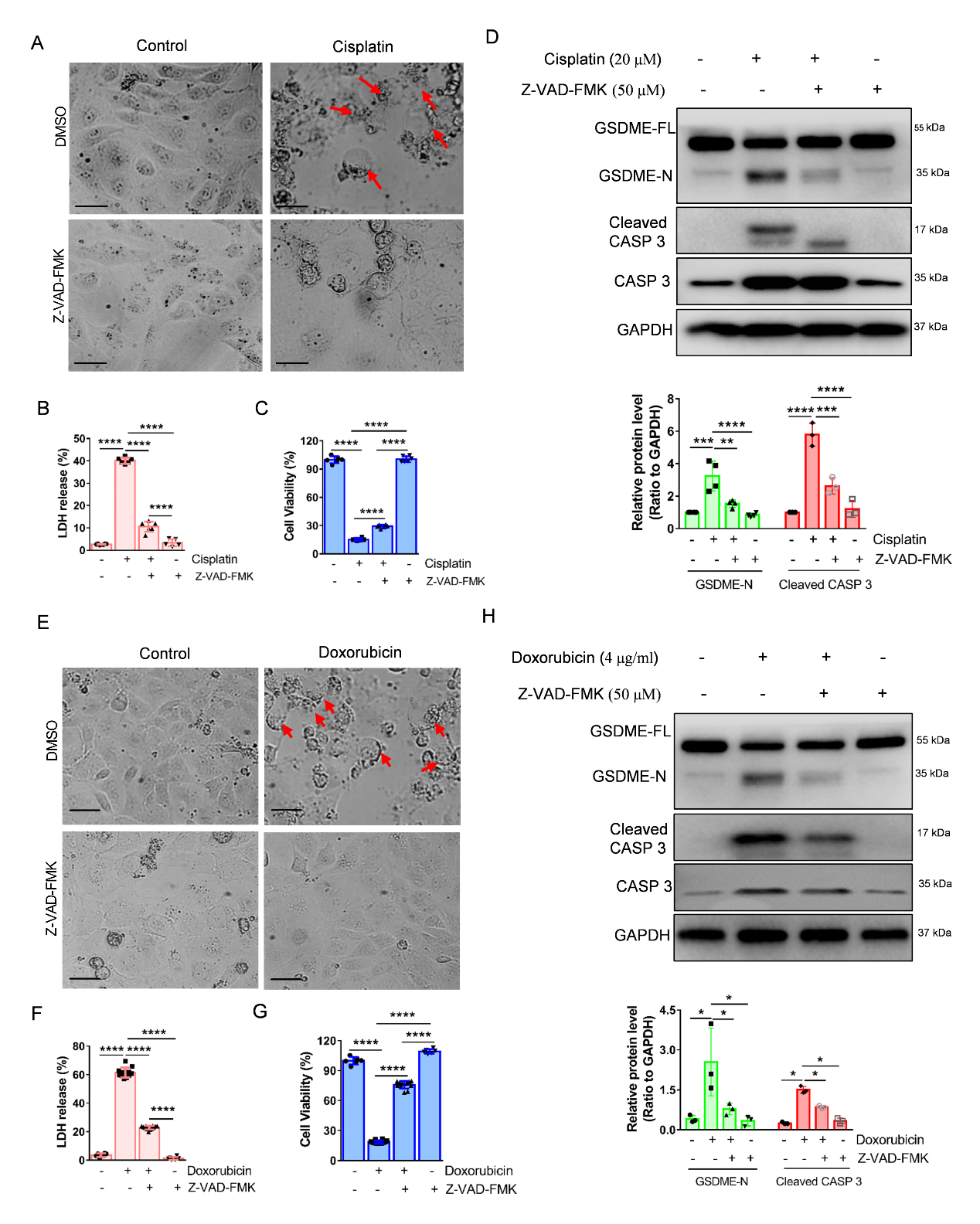


**Figure S3.** **Z-VAD-FMK inhibits** **cisplatin- and doxorubicin-induced pyroptosis in HK-2 cells. a, e** Representative light microscopy images of HK-2 cells treated with cisplatin (20 μM) or doxorubicin (4 μg/ml) before or after Z-VAD-FMK (50 μM) intervention. Red arrow demonstrated bubbles emerging from the plasma membrane. Scale bar, 50 μm. Cytotoxicity and cell viability were determined using the LDH assay (**b, f**) and CCK-8 detection (**c, g**) for HK-2 cells treated with cisplatin (20 μM) or doxorubicin (4 μg/ml) in the presence or absence of Z-VAD-FMK (50 μM). Western blot analysis of GSDME and CASP 3 cleavage in cisplatin (20 μM) (**d**) or doxorubicin (4 μg/ml) (**h**) treated HK-2 cells in the presence or absence of Z-VAD-FMK (50 μM). All data were shown as mean ± SD from three independent experiments (*n* = 3). * *p* < 0.05, ** *p* < 0.01, *** *p* < 0.001, **** *p* < 0.0001 using one-way ANOVA followed by Turkey's method.


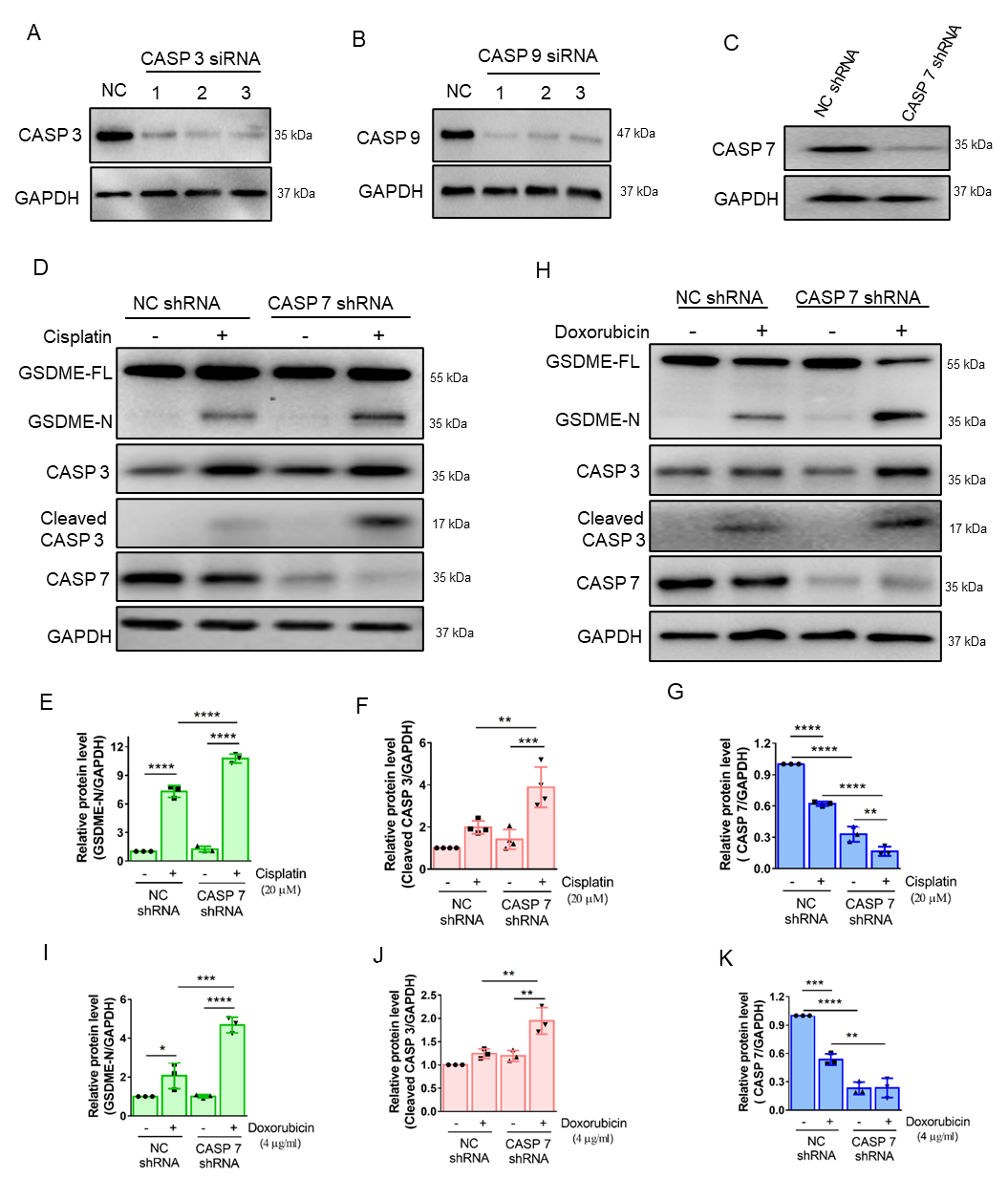


**Figure S4.** **CASP 7 siRNA augments cisplatin or doxorubicin-induced GSDME and CASP 3 cleavage in HK-2 cells.** Western blot analysis of the expression of CASP 3 **(a)**, CASP 9 **(b)**, and CASP 7 **(c)** in control and CASP 3/9/7 siRNA transfected HK-2 cells. **d-g** Western blot analysis of the cleavage of GSDME and caspase 3 in cisplatin (20 μM) treated HK-2 cells in the presence or absence of CASP 7 siRNA. **h-k** Western blot analysis of the cleavage of GSDME and CASP 3 in doxorubicin (4 μg/ml) treated HK-2 cells in the presence or absence of CASP 7 siRNA. All data were shown as mean ± SD from three independent experiments (*n* = 3). * *p* < 0.05, ** *p* < 0.01, *** *p* < 0.001, **** *p* < 0.0001 using one-way ANOVA followed by Turkey's method.


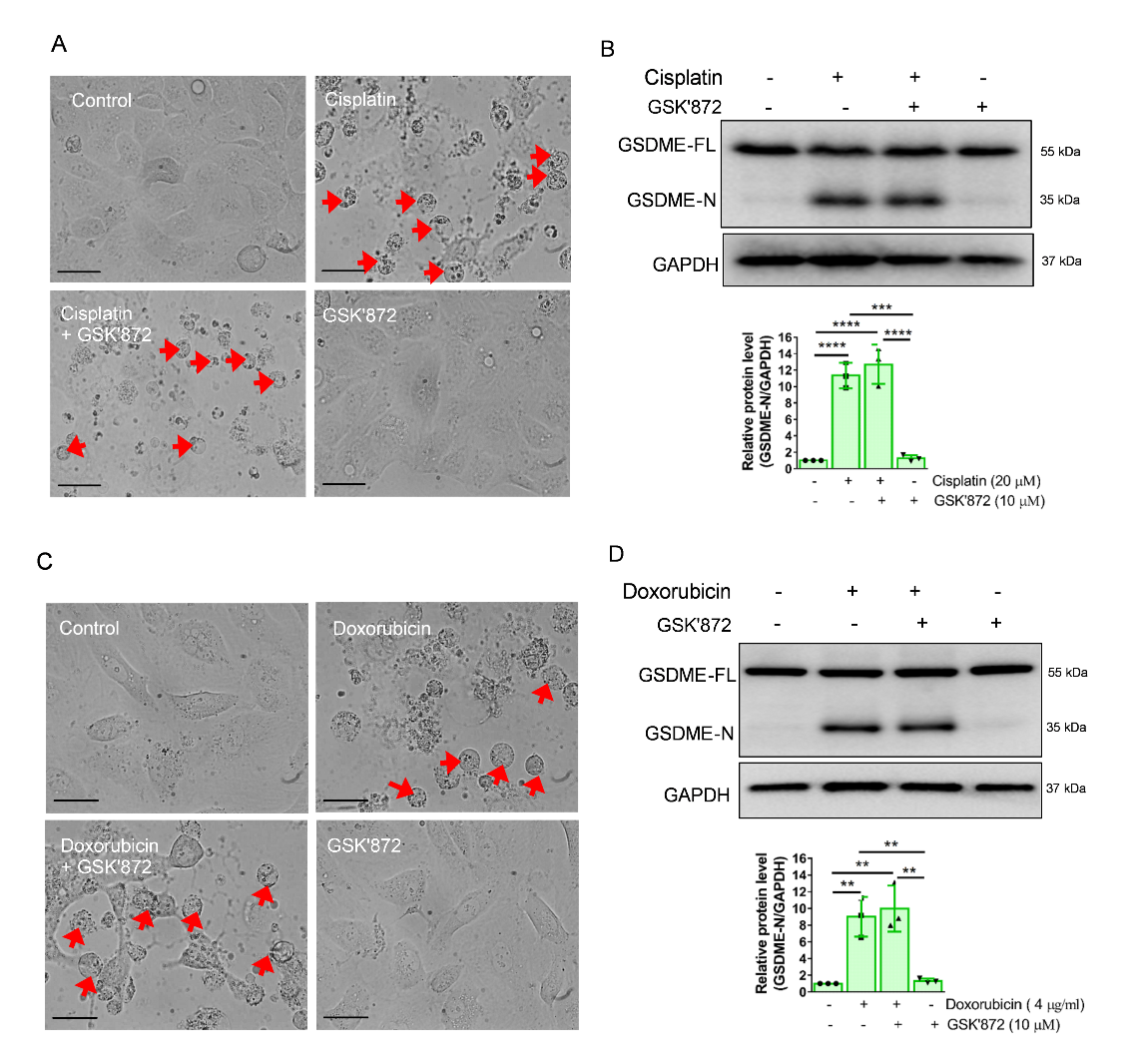


**Figure S5. Necroptosis inhibitor GSK’872 did not decrease pyroptosis of HK-2 cells. a, c** Representative microscopy images of GSK’872 pre-treated HK-2 cells incubated with cisplatin (20 μM) or doxorubicin (4 μg/ml) for 48 hours. Scale bar, 50 μm. **b, d** Western blot analysis of the cleavage of GSDME in cisplatin (20 μM) or doxorubicin (4 μg/ml) treated HK-2 cells in the presence or absence of GSK’872 (10 μM). All data were shown as mean ± SD from three independent experiments (*n* = 3). ** *p* < 0.01, *** *p* < 0.001, **** *p* < 0.0001 using one-way ANOVA followed by Turkey's method.


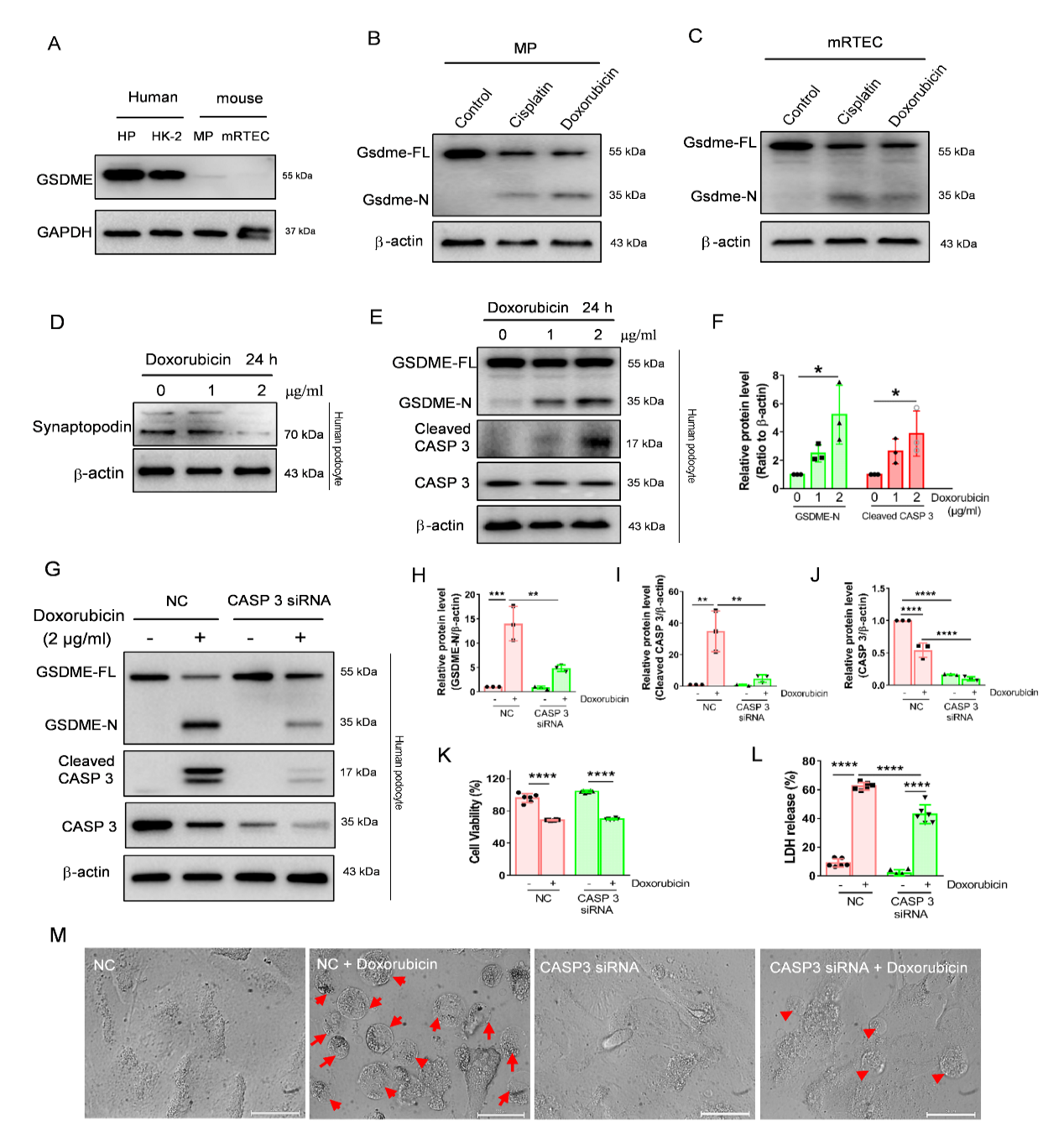


**Figure S6.** **CASP 3 siRNA alleviates doxorubicin-induced pyroptosis in human podocytes. a** Western blot analysis of the expression of GSDME in human tubular cells (HK-2), human podocyte (HP), mouse renal tubular epithelial cells (mRTEC), and moues podocytes (MP). **b, c** Expression of Gsdme in cisplatin and doxorubicin treated MP and mRTEC. **d-f** Western blot expression of synaptopodin, GSDME, and CASP 3 in doxorubicin-treated human podocytes for 24h. **g-j** Western blot analysis of GSDME cleavage and CASP 3 in doxorubicin-treated human podocytes in the presence or absence of CASP 3 siRNA. **k, l** CCK-8 and LDH detection for human podocytes treated with doxorubicin (2 μg/ml) in the presence or absence of CASP 3 siRNA. **m** Representative light microscopy images of human podocytes treated with doxorubicin (2 μg/ml) in the presence or absence of CASP 3 siRNA. A red arrow indicates bubbles emerging from the plasma membrane. Scale bar, 50 μm. All data are shown as means ± SD from three independent experiments (*n* = 3). * *p* < 0.05, ** *p* < 0.01, *** *p* < 0.001, **** *p* < 0.0001 using one-way ANOVA followed by Tukey's method. HP: human podocyte; MP: mouse podocyte; mRTEC: mouse renal tubular epithelial cell.


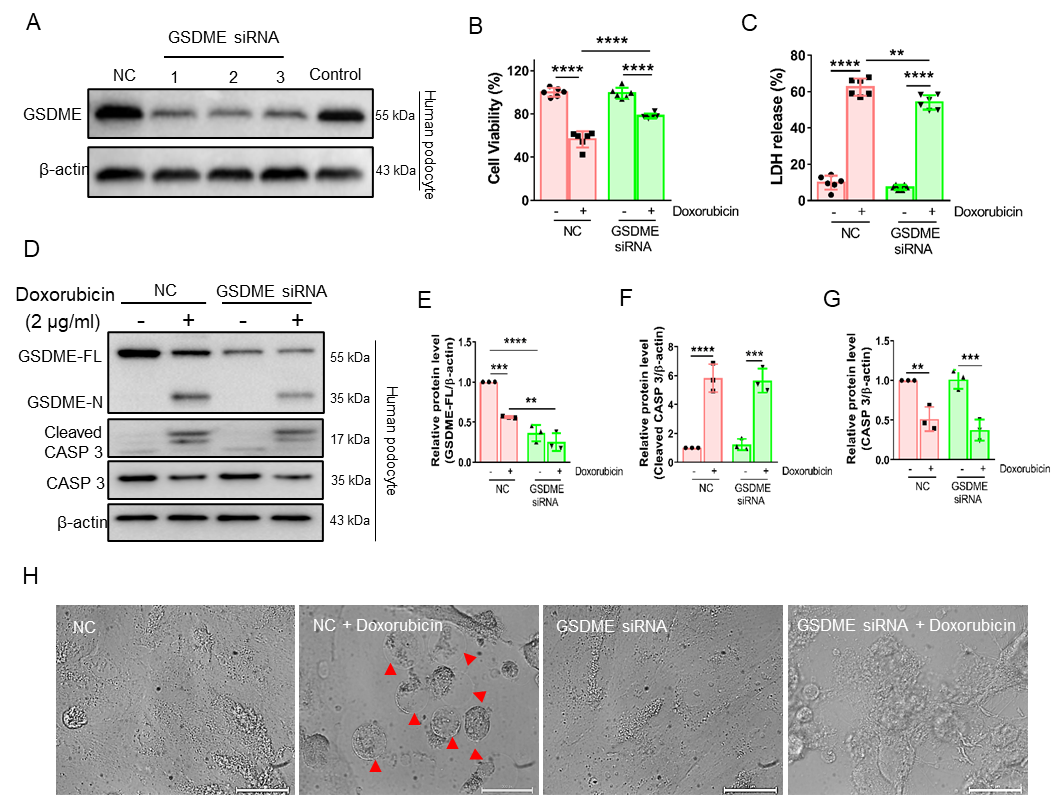


**Figure S7.** **GSDME siRNA alleviates doxorubicin-induced pyroptosis in human podocytes. a** Western blot expression of GSDME in GSDME siRNA-treated human podocytes. **b, c** CCK-8 and LDH detection for human podocytes treated with GSDME siRNA. **d-g** Western blot analysis of GSDME cleavage and CASP 3 in doxorubicin-treated human podocytes in the presence or absence of GSDME siRNA. **h** Representative light microscopy images of human podocytes treated with doxorubicin (2 μg/ml) in the presence or absence of GSDME siRNA. A red arrow indicates bubbles emerging from the plasma membrane. Scale bar, 50 μm. All data are shown as means ± SD from three independent experiment (*n* = 3)s. ** *p* < 0.01, *** *p* < 0.001, **** *p* < 0.0001 using one-way ANOVA followed by Tukey's method.


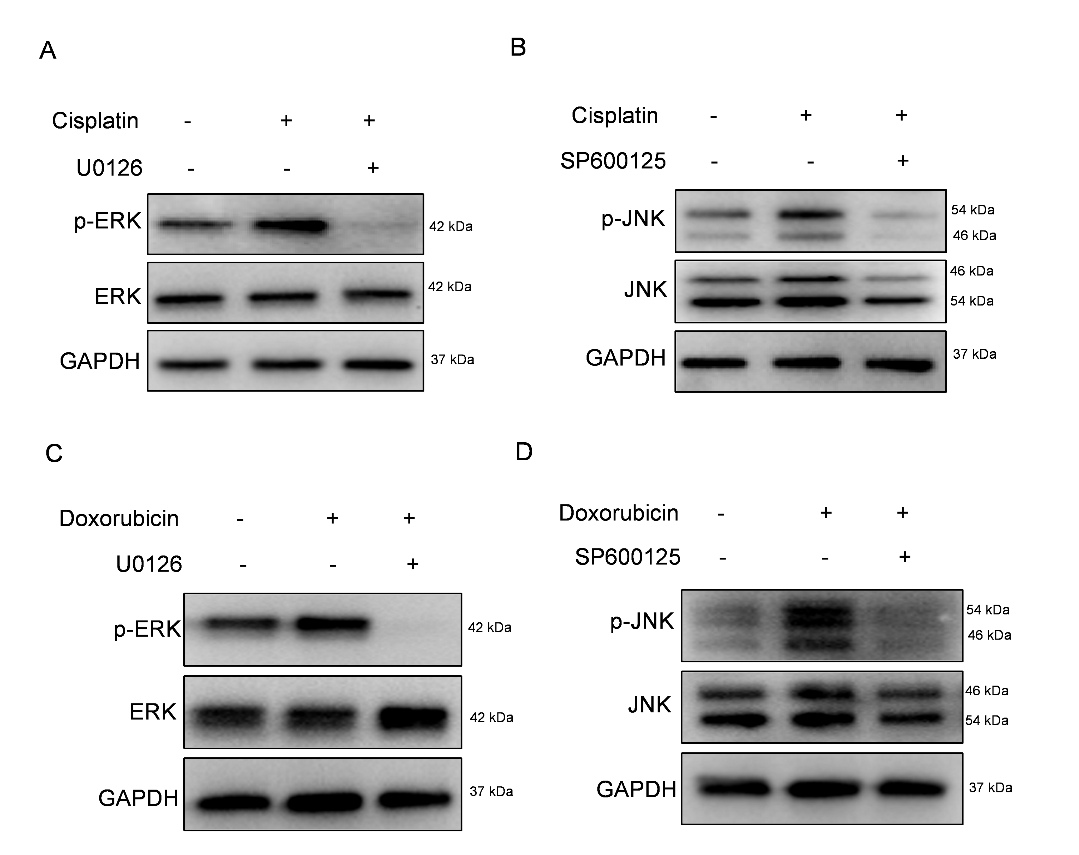


**Figure S8. The inhibitory effect of ERK and JNK inhibitor in HK-2 cells. a, b** Western blot detection of the phosphorylation of ERK and JNK in cisplatin (20 μM) induced HK-2 cells for 3 hours before or after U0126 (10 μM) and SP600125 (10 μM) intervention. **c, d** Western blot detection of the phosphorylation of ERK and JNK in doxorubicin (4 μg/ml) induced HK-2 cells for 3 hours before or after U0126 (10 μM) and SP600125 (10 μM) intervention.
