# Supplementary material for: Caspase 3/GSDME-dependent pyroptosis contributes to chemotherapy drug-induced nephrotoxicity
Source: Cell Death Dis. 2021 Feb 15;12(2):186. doi: 10.1038/s41419-021-03458-5 (PMC7884686; doi:10.1038/s41419-021-03458-5)
Supplement: Supplementary file 2 — Supplement table [file 41419_2021_3458_MOESM2_ESM.docx]

**Caspase 3/GSDME-dependent pyroptosis contributes to chemotherapy drug-induced nephrotoxicity**

**Running title:** Pyroptosis in chemotherapeutic nephrotoxicity

Xiujin Shen^1*^. Haibing Wang^2*^. Chunhua Weng^1^. Hong Jiang^1^. Jianghua Chen^1*^

^1^Kidney Disease Center, the First Affiliated Hospital, College of Medicine, Zhejiang University; Key Laboratory of Kidney Disease Prevention and Control Technology, Zhejiang Province; National Key Clinical Department of Kidney Diseases; Institute of Nephrology, Zhejiang University; the Third Grade Laboratory under the National State, Administration of Traditional Chinese Medicine, Hangzhou, China

^2^ Central Laboratory, the First Affiliated Hospital of Zhejiang Chinese Medical University, Hangzhou, China.

*** Correspondence**

Xiujin Shen

E-mail: xiujinshen@zju.edu.cn.

Haibing Wang

E-mail: [whb2016@zcmu.edu.cn](mailto:whb2016@zcmu.edu.cn).

Jianghua Chen

E-mail: [chenjianghua@zju.edu.cn](mailto:chenjianghua@zju.edu.cn).

**Table S1** Related siRNA and shRNA sequences used in the experiment

| **Gene name** | **Forward** | **Reverse** |
| --- | --- | --- |
| **Caspase 3 siRNA** | | |
| 1 | 5′-GGAACCAAAGAUCAUACAUTT-3′ | 5′-AUGUAUGAUCUUUGGUUCCTT-3′ |
| 2 | 5′-GCCGACUUCUUGUAUGCAUTT-3′ | 5′-AUGCAUACAAGAAGUCGGCTT-3′ |
| 3 | 5′-CCGACAAGCUUGAAUUUAUTT-3′ | 5′-AUAAAUUCAAGCUUGUCGGTT-3′ |
| **Caspase 9 siRNA** | | |
| 1 | 5′-GCAAAGUUGUCGAAGCCAATT-3′ | 5′-UUGGCUUCGACAACUUUGCTT-3′ |
| 2 | 5′-GCAGAAAGACCAUGGGUUUTT-3′ | 5′-AAACCCAUGGUCUUUCUGCTT-3′ |
| 3 | 5′-GCCUCAUUAUCAACAAUGUTT-3′ | 5′-ACAUUGUUGAUAAUGAGGCTT -3′ |
| **GSDME siRNA** | | |
| 1 | 5′-CCAUUGCCUACGGUGUCAUTT-3′ | 5′-AUGACACCGUAGGCAAUGGTT-3′ |
| 2 | 5′-GCAGCAAGCAGCUGUUUAUTT-3′ | 5′-AUAAACAGCUGCUUGCUGCTT-3′ |
| 3 | 5′-GCUUUAGGCAGAGAACAUUTT-3′ | 5′-AAUGUUCUCUGCCUAAAGCTT-3′ |
| **CASP 7 shRNA** | | |
|  | CCGGTACTTCAGTCAATAGCCATATCTCGAGATATGGCTATTGACTGAAGTATTTTTG | |

**Table S2** Related primers used in the experiment

| **Gene name** | **Forward** | **Reverse** |
| --- | --- | --- |
| ***Kim1*** | 5′-GTGGAAGTAAAGGGGGTGGT-3′ | 5′-TGCCCCTTTAAGTTGTACCG-3′ |
| ***Ngal*** | 5′-ACTACAACCAGTTCGCCATG-3′ | 5′-TGATGTTGTCGTCCTTGAGG-3′ |
| ***Il6*** | 5′-ACACATGTTCTCTGGGAAATCGT-3′ | 5′-AAGTGCATCATCGTTGTTCATACA-3′ |
| ***Tnfa*** | 5′-GACGTGGAACTGGCAGAAGAG-3′ | 5′-TTGGTGGTTTGTGAGTGTGAG-3′ |
| ***Il1b*** | 5′-GCAACTGTTCCTGAACTCAACT-3′ | 5′-ATCTTTTGGGGTCCGTCAACT-3′ |
| ***Gapdh*** | 5′-AGGTCGGTGTGAACGGATTTG-3′ | 5′-TGTAGACCATGTAGTTGAGGTCA-3′ |
