# Supplementary material for: Caspase 3/GSDME-dependent pyroptosis contributes to chemotherapy drug-induced nephrotoxicity
Source: Cell Death Dis. 2021 Feb 15;12(2):186. doi: 10.1038/s41419-021-03458-5 (PMC7884686; doi:10.1038/s41419-021-03458-5)
Supplement: Supplementary file 3 — Supplement material-original western blot pictures [file 41419_2021_3458_MOESM3_ESM.pptx]

## Slide 1
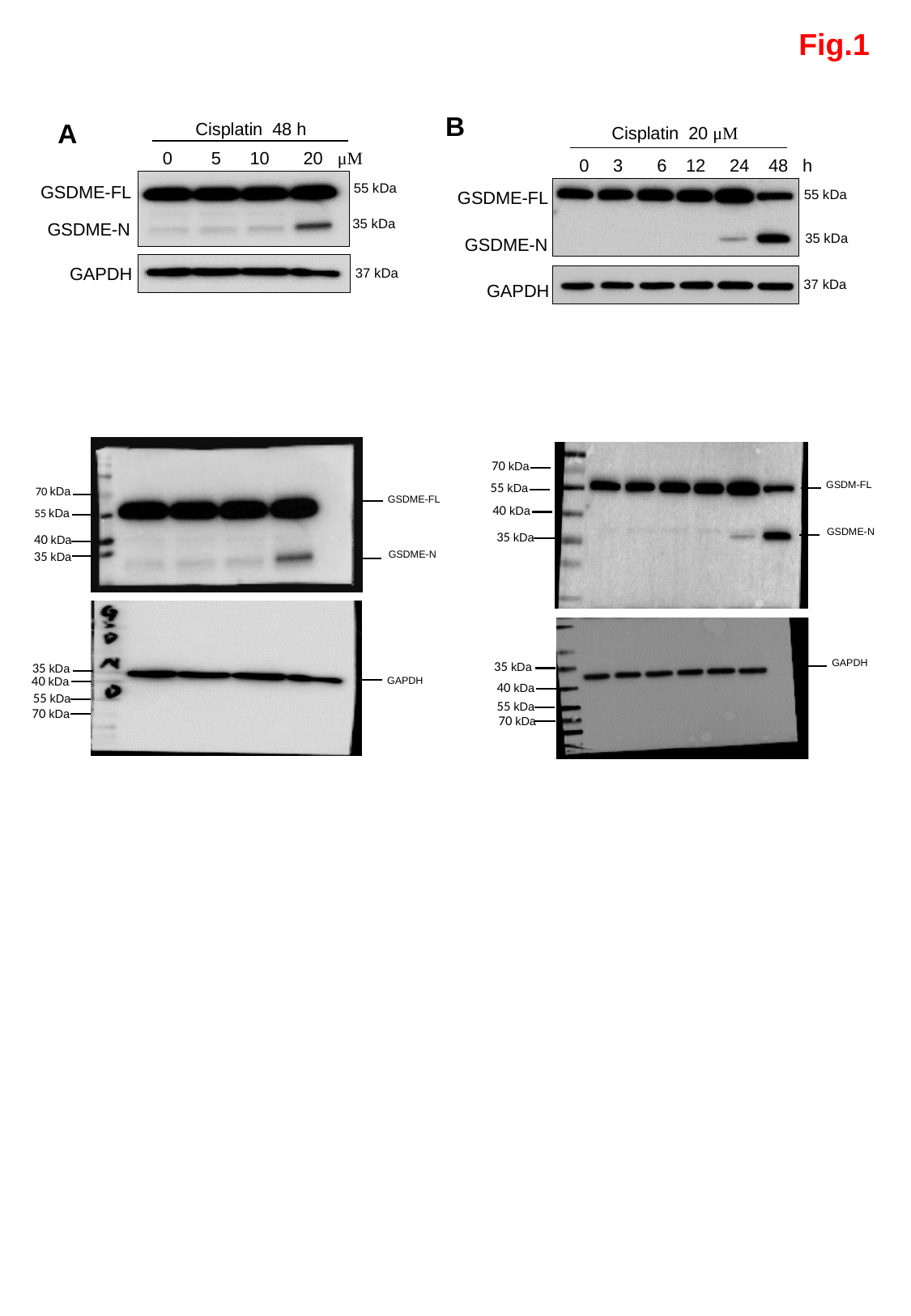

Fig.1
B
A
Cisplatin 48 h
Cisplatin 20 μM
0 5 10 20 μM
0 3 6 12 24 48 h
55 kDa
GSDME-FL
55 kDa
GSDME-FL
35 kDa
GSDME-N
35 kDa
GSDME-N
GAPDH
37 kDa
37 kDa
GAPDH
70 kDa
55 kDa
40 kDa
35 kDa
GSDM-FL
70 kDa
GSDME-FL
55 kDa
GSDME-N
40 kDa
35 kDa
GSDME-N
GAPDH
35 kDa
35 kDa
40 kDa
GAPDH
40 kDa
55 kDa
55 kDa
70 kDa
70 kDa

## Slide 2
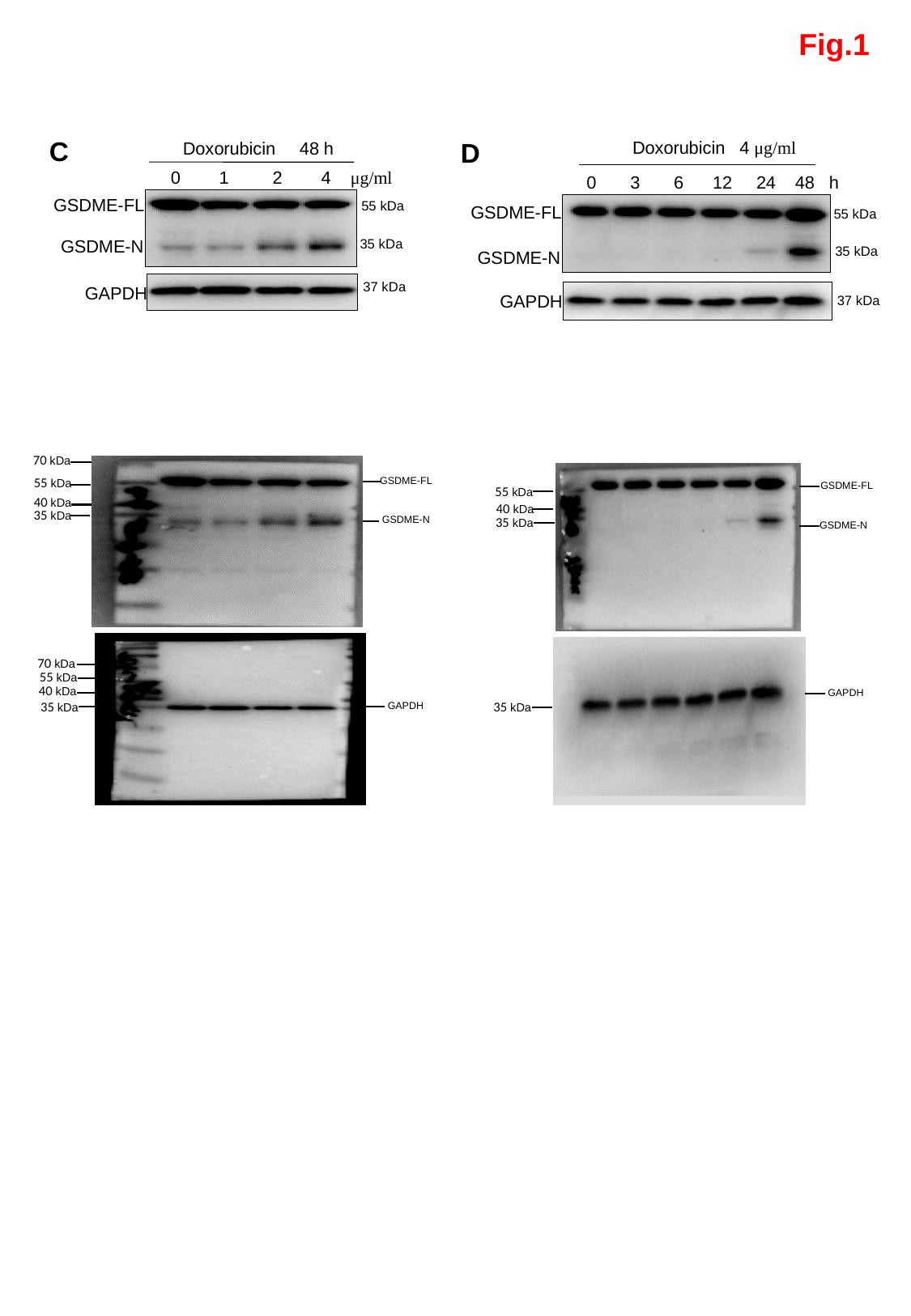

Fig.1
C
D
Doxorubicin 4 μg/ml
Doxorubicin 48 h
0 1 2 4 μg/ml
0 3 6 12 24 48 h
GSDME-FL
55 kDa
GSDME-FL
55 kDa
35 kDa
GSDME-N
35 kDa
GSDME-N
37 kDa
GAPDH
GAPDH
37 kDa
70 kDa
55 kDa
GSDME-FL
GSDME-FL
55 kDa
40 kDa
40 kDa
35 kDa
GSDME-N
35 kDa
GSDME-N
GAPDH
35 kDa
70 kDa
55 kDa
40 kDa
35 kDa
GAPDH

## Slide 3
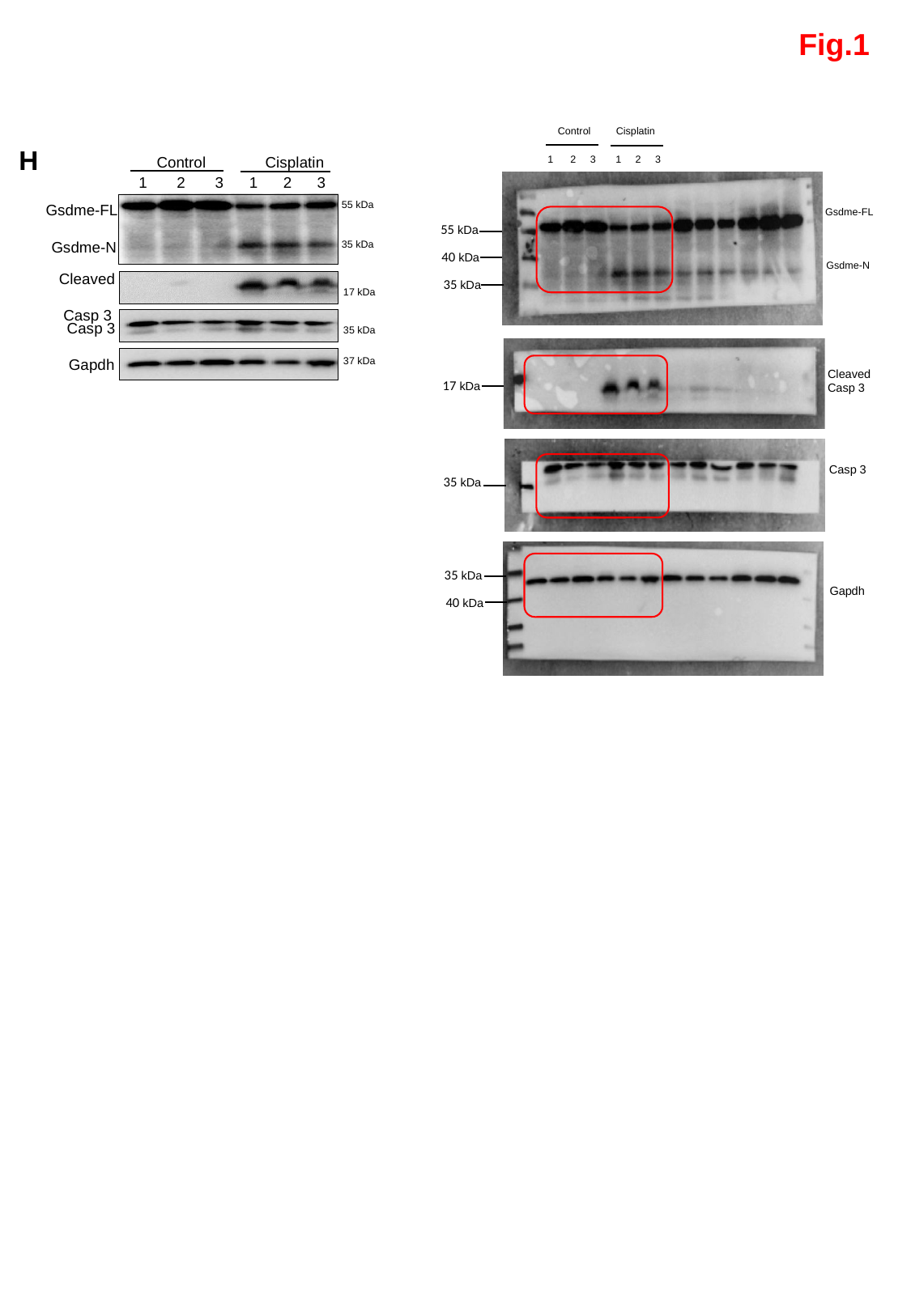

Fig.1
Control Cisplatin
1 2 3 1 2 3
Gsdme-FL
55 kDa
40 kDa
Gsdme-N
35 kDa
Cleaved
Casp 3
17 kDa
 Casp 3
35 kDa
35 kDa
 Gapdh
40 kDa
H
Control Cisplatin
1 2 3 1 2 3
55 kDa
Gsdme-FL
Gsdme-N
35 kDa
Cleaved
 Casp 3
17 kDa
Casp 3
35 kDa
37 kDa
Gapdh

## Slide 4
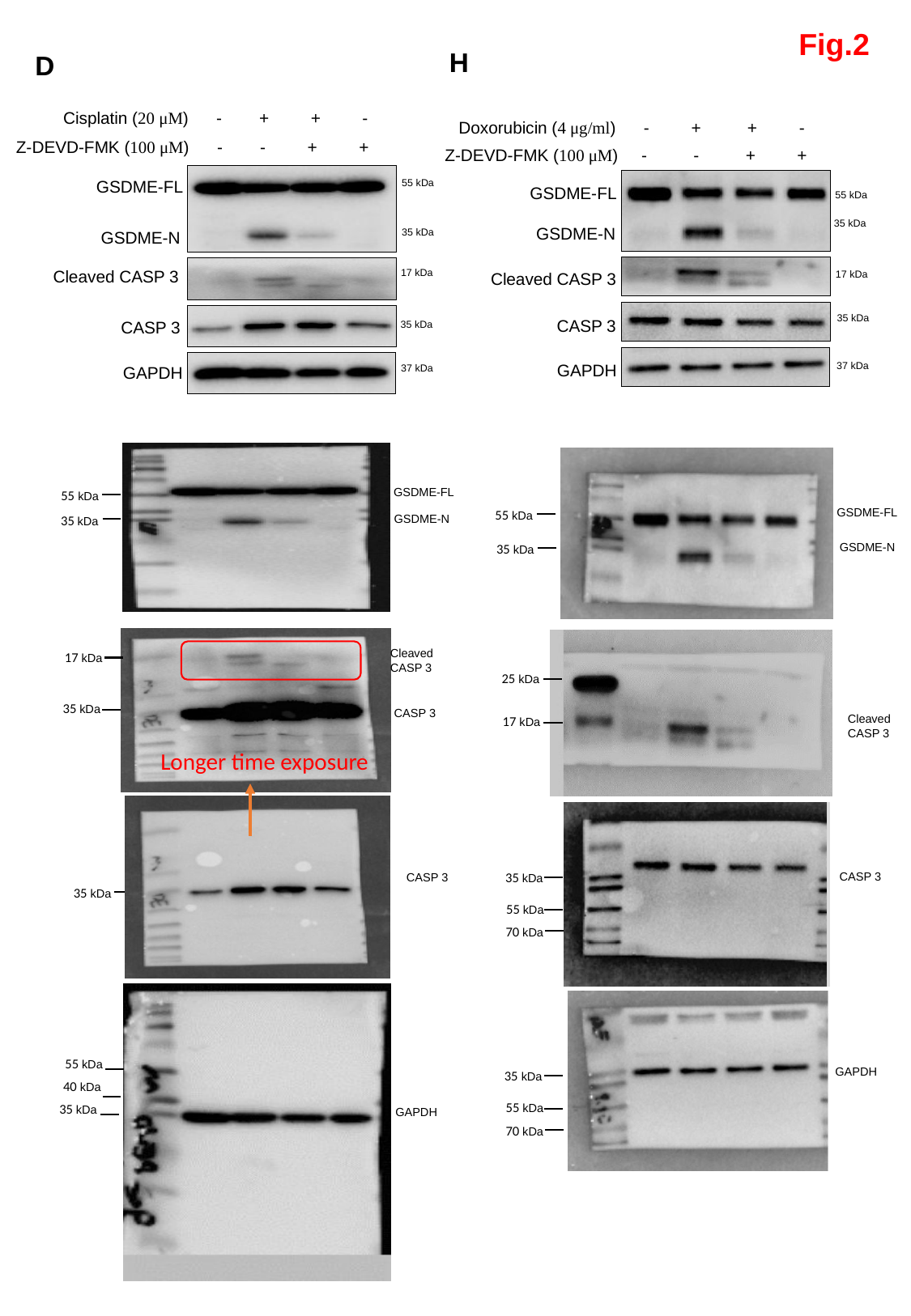

Fig.2
H
D
Cisplatin (20 μM) - + + -
Z-DEVD-FMK (100 μM) - - + +
GSDME-FL
55 kDa
35 kDa
GSDME-N
Cleaved CASP 3
17 kDa
CASP 3
35 kDa
GAPDH
37 kDa
Doxorubicin (4 μg/ml) - + + -
Z-DEVD-FMK (100 μM) - - + +
GSDME-FL
55 kDa
35 kDa
GSDME-N
17 kDa
Cleaved CASP 3
35 kDa
CASP 3
37 kDa
GAPDH
GSDME-FL
55 kDa
GSDME-N
35 kDa
25 kDa
Cleaved
CASP 3
17 kDa
35 kDa
 CASP 3
55 kDa
70 kDa
GAPDH
35 kDa
55 kDa
70 kDa
GSDME-FL
55 kDa
GSDME-N
35 kDa
Cleaved
CASP 3
17 kDa
35 kDa
 CASP 3
Longer time exposure
 CASP 3
35 kDa
55 kDa
40 kDa
35 kDa
GAPDH

## Slide 5
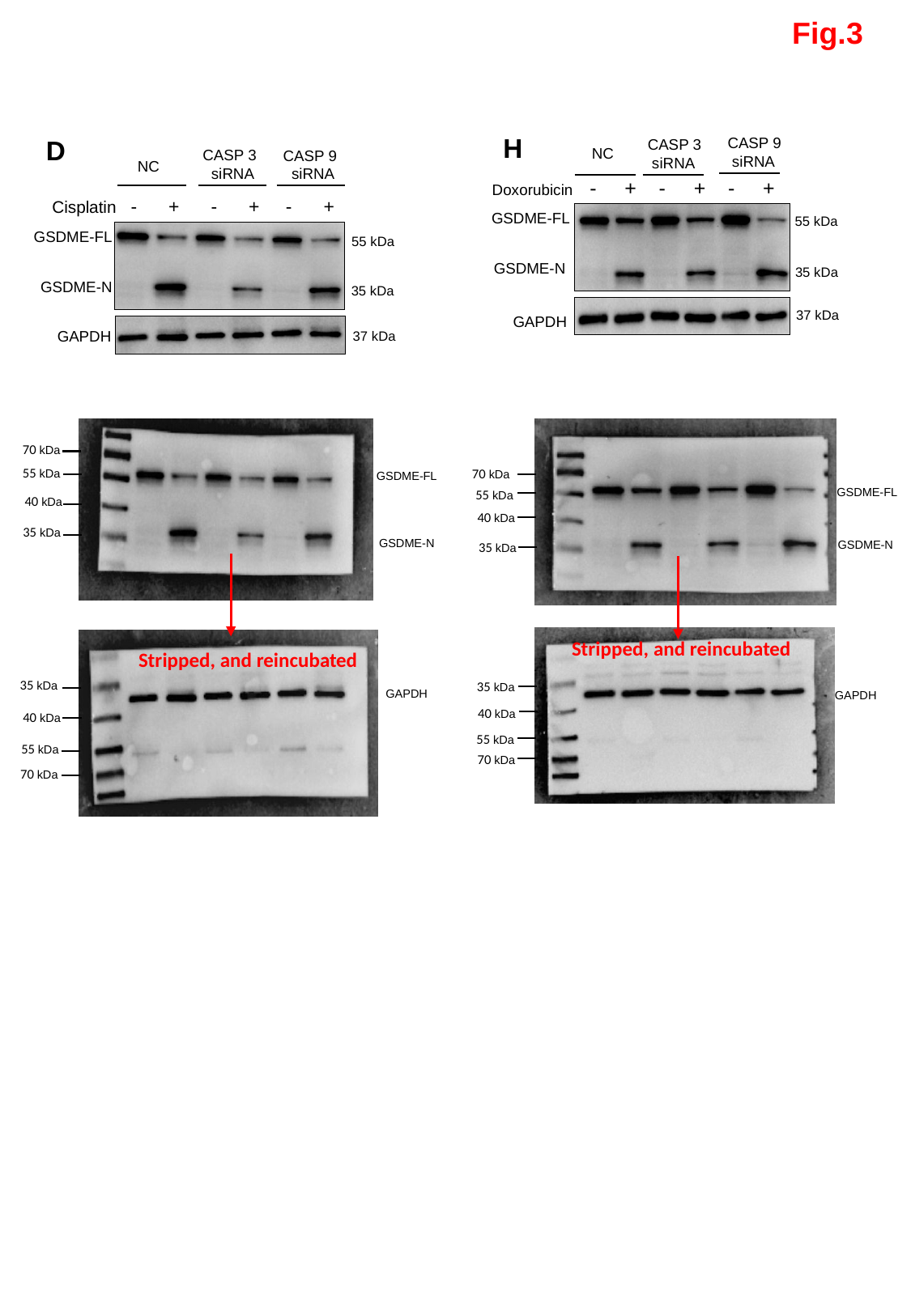

Fig.3
H
D
CASP 9
 siRNA
CASP 3
 siRNA
NC
Doxorubicin - + - + - +
GSDME-FL
GSDME-N
GAPDH
55 kDa
35 kDa
37 kDa
CASP 3
 siRNA
CASP 9
 siRNA
NC
Cisplatin - + - + - +
GSDME-FL
GSDME-N
GAPDH
55 kDa
35 kDa
37 kDa
70 kDa
55 kDa
GSDME-FL
40 kDa
35 kDa
GSDME-N
Stripped, and reincubated
35 kDa
GAPDH
40 kDa
55 kDa
70 kDa
70 kDa
GSDME-FL
55 kDa
40 kDa
GSDME-N
35 kDa
Stripped, and reincubated
35 kDa
GAPDH
40 kDa
55 kDa
70 kDa

## Slide 6
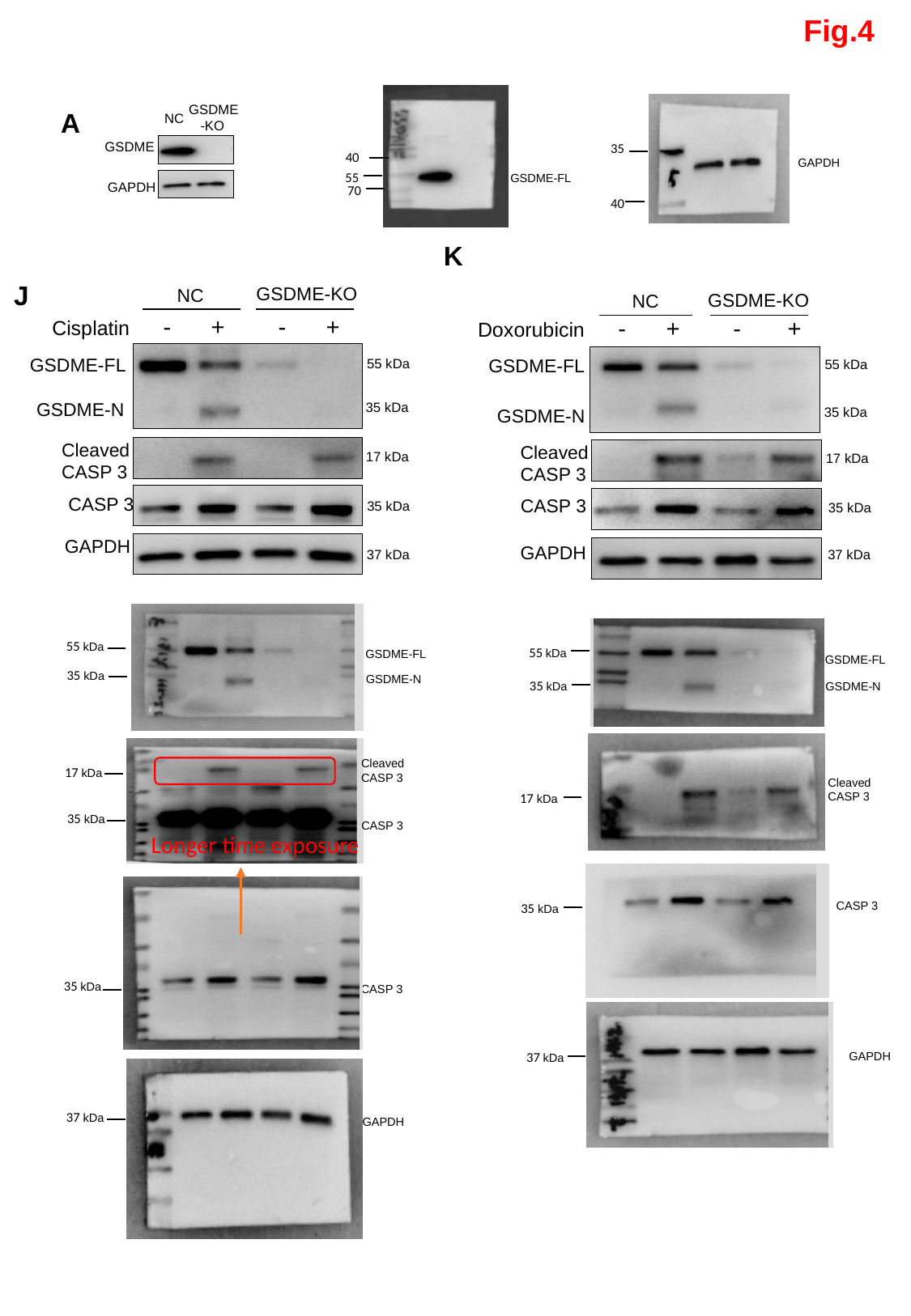

Fig.4
40
55
GSDME-FL
70
35
GAPDH
40
GSDME
 -KO
NC
GSDME
GAPDH
A
K
J
GSDME-KO
NC
Cisplatin - + - +
GSDME-FL
GSDME-N
CASP 3
GAPDH
Cleaved
CASP 3
GSDME-KO
NC
Doxorubicin - + - +
GSDME-FL
55 kDa
35 kDa
GSDME-N
Cleaved
CASP 3
17 kDa
CASP 3
35 kDa
GAPDH
37 kDa
55 kDa
35 kDa
17 kDa
35 kDa
37 kDa
55 kDa
GSDME-FL
35 kDa
GSDME-N
55 kDa
GSDME-FL
35 kDa
GSDME-N
Cleaved
CASP 3
17 kDa
Cleaved
CASP 3
17 kDa
35 kDa
 CASP 3
Longer time exposure
35 kDa
 CASP 3
37 kDa
GAPDH
 CASP 3
35 kDa
GAPDH
37 kDa

## Slide 7
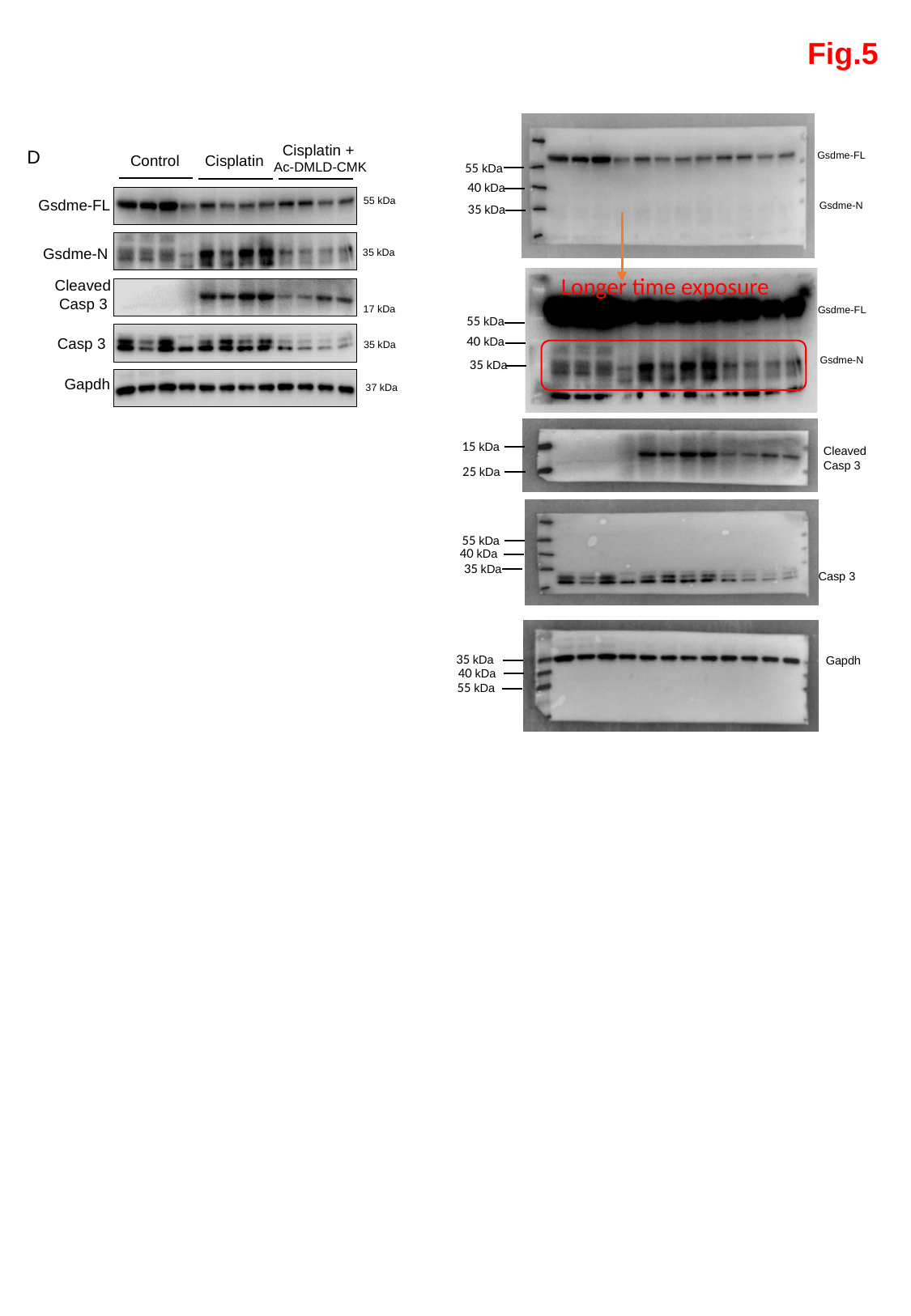

Fig.5
Gsdme-FL
55 kDa
40 kDa
Gsdme-N
35 kDa
 Cisplatin +
Ac-DMLD-CMK
D
Control Cisplatin
55 kDa
Gsdme-FL
Gsdme-N
35 kDa
Cleaved
 Casp 3
17 kDa
Casp 3
35 kDa
Gapdh
37 kDa
Longer time exposure
Gsdme-FL
55 kDa
40 kDa
Gsdme-N
35 kDa
15 kDa
Cleaved
Casp 3
25 kDa
55 kDa
40 kDa
35 kDa
 Casp 3
35 kDa
 Gapdh
40 kDa
55 kDa

## Slide 8
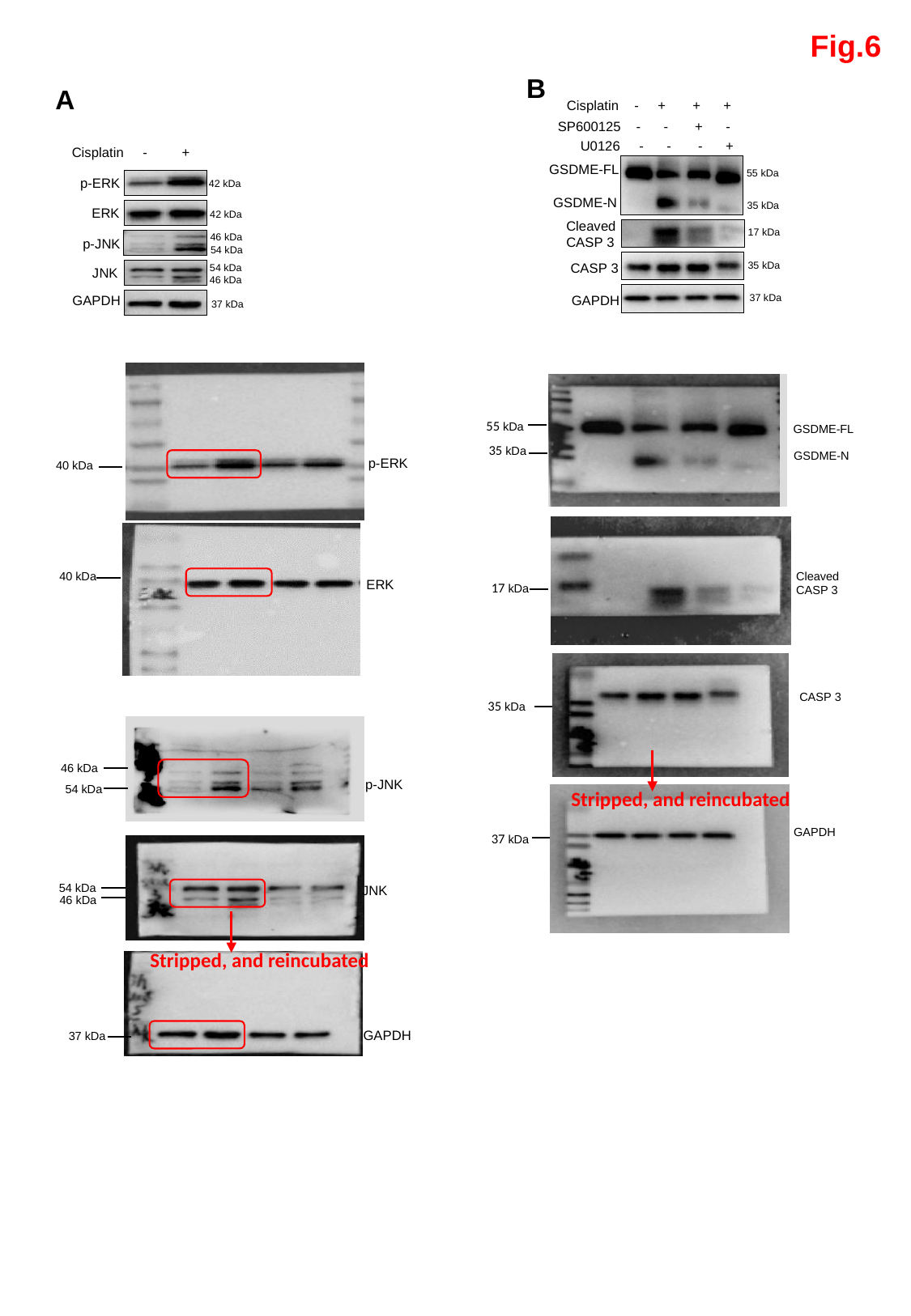

Fig.6
B
Cisplatin - + + +
SP600125 - - + -
U0126 - - - +
GSDME-FL
GSDME-N
Cleaved
CASP 3
CASP 3
GAPDH
55 kDa
35 kDa
17 kDa
35 kDa
37 kDa
A
Cisplatin - +
p-ERK
42 kDa
ERK
42 kDa
46 kDa
p-JNK
54 kDa
54 kDa
JNK
46 kDa
GAPDH
37 kDa
55 kDa
GSDME-FL
35 kDa
GSDME-N
Cleaved
CASP 3
17 kDa
 CASP 3
35 kDa
GAPDH
37 kDa
p-ERK
40 kDa
40 kDa
ERK
46 kDa
p-JNK
54 kDa
Stripped, and reincubated
54 kDa
JNK
46 kDa
Stripped, and reincubated
GAPDH
37 kDa

## Slide 9
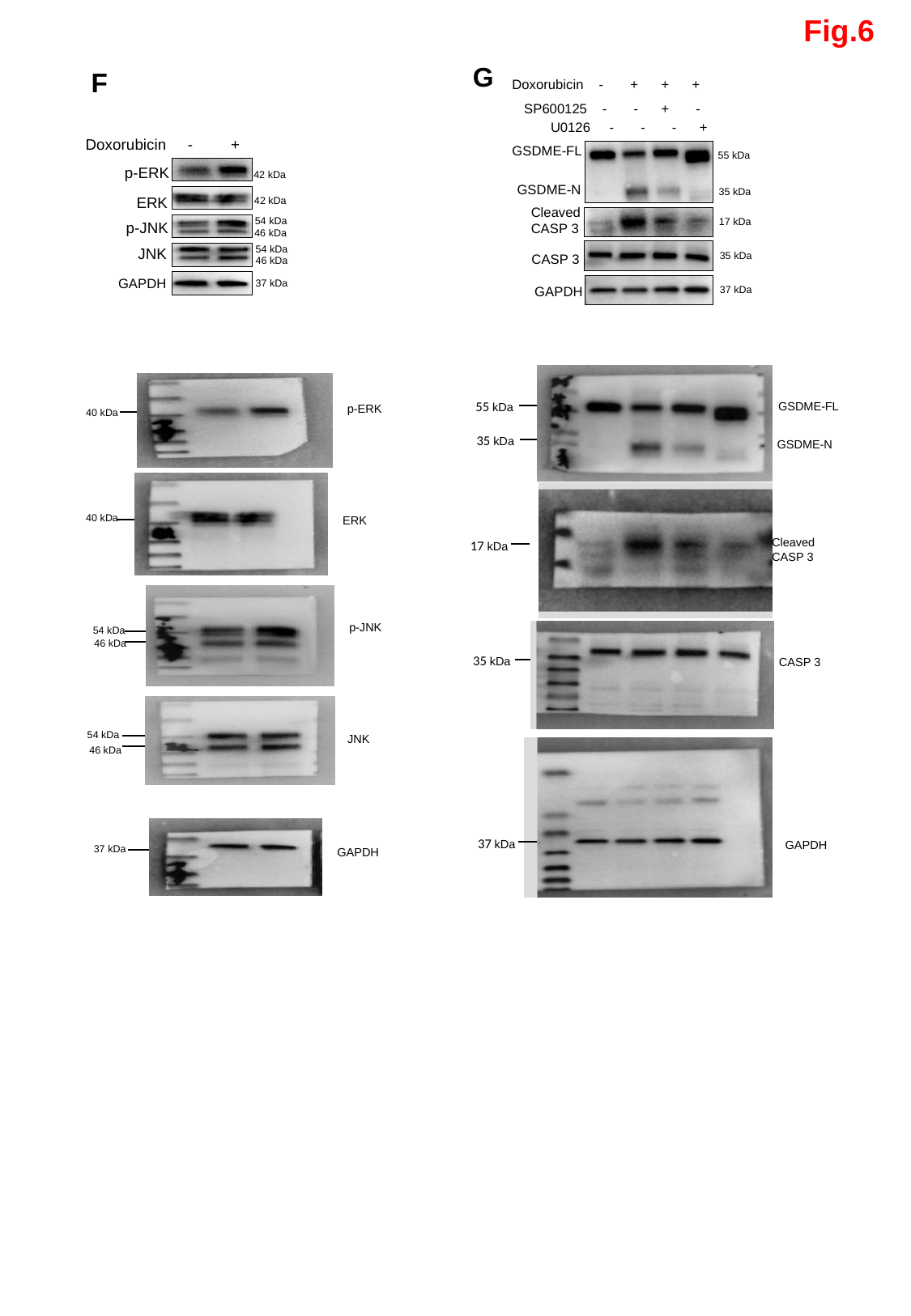

Fig.6
G
F
Doxorubicin - + + +
SP600125 - - + -
U0126 - - - +
GSDME-FL
55 kDa
GSDME-N
35 kDa
Cleaved
CASP 3
17 kDa
35 kDa
CASP 3
GAPDH
37 kDa
Doxorubicin - +
p-ERK
42 kDa
ERK
42 kDa
54 kDa
p-JNK
46 kDa
54 kDa
JNK
46 kDa
GAPDH
37 kDa
55 kDa
GSDME-FL
35 kDa
GSDME-N
Cleaved
CASP 3
17 kDa
35 kDa
 CASP 3
37 kDa
GAPDH
p-ERK
40 kDa
40 kDa
ERK
p-JNK
54 kDa
46 kDa
54 kDa
JNK
46 kDa
37 kDa
GAPDH

## Slide 10
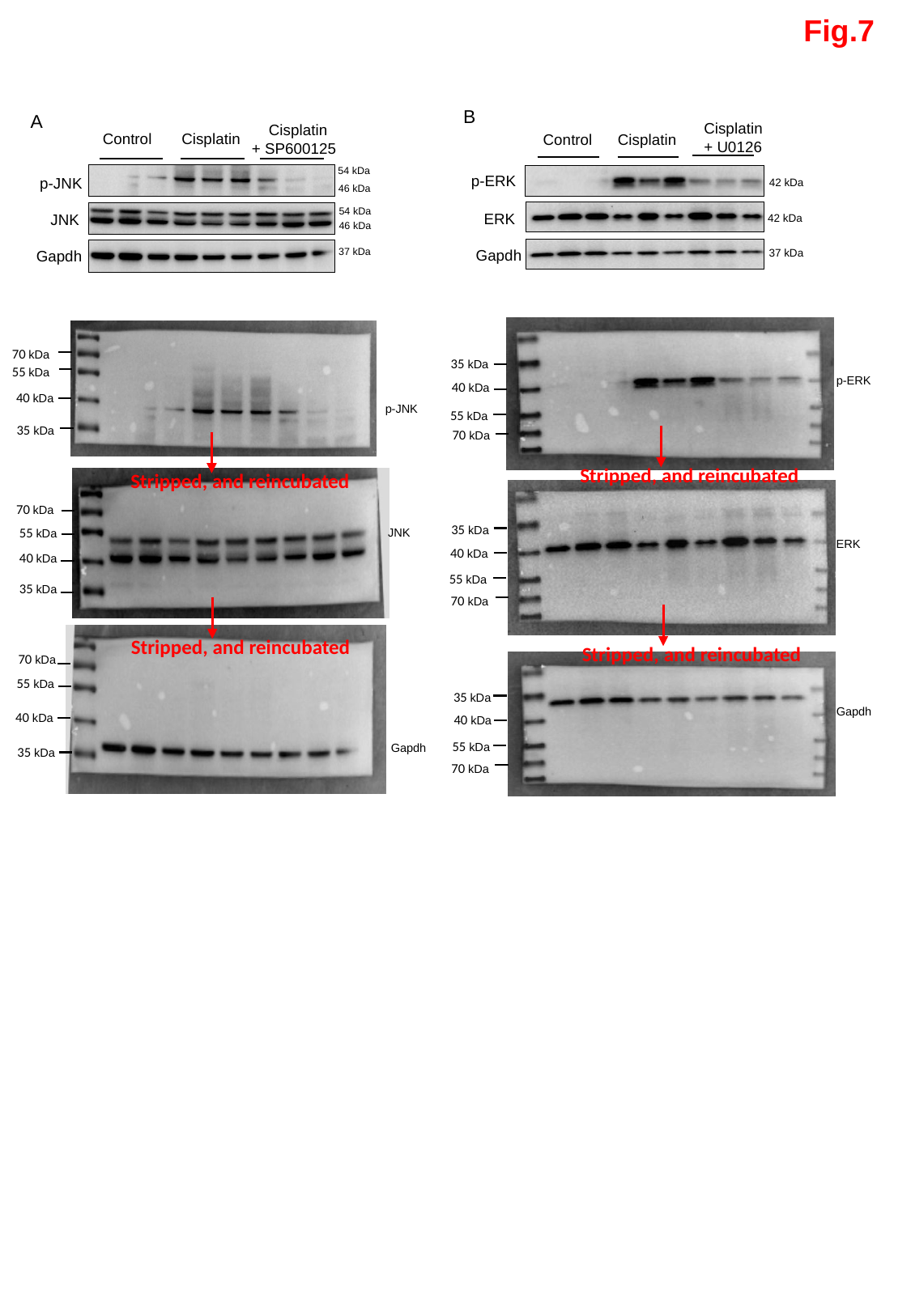

Fig.7
B
A
Cisplatin
+ U0126
Control Cisplatin
p-ERK
ERK
Gapdh
42 kDa
42 kDa
37 kDa
 Cisplatin
 + SP600125
Control Cisplatin
p-JNK
JNK
Gapdh
54 kDa
46 kDa
54 kDa
46 kDa
37 kDa
35 kDa
40 kDa
55 kDa
70 kDa
p-ERK
35 kDa
ERK
40 kDa
55 kDa
70 kDa
35 kDa
Gapdh
40 kDa
55 kDa
70 kDa
70 kDa
55 kDa
40 kDa
p-JNK
35 kDa
70 kDa
55 kDa
JNK
40 kDa
35 kDa
70 kDa
55 kDa
40 kDa
Gapdh
35 kDa
Stripped, and reincubated
Stripped, and reincubated
Stripped, and reincubated
Stripped, and reincubated

## Slide 11
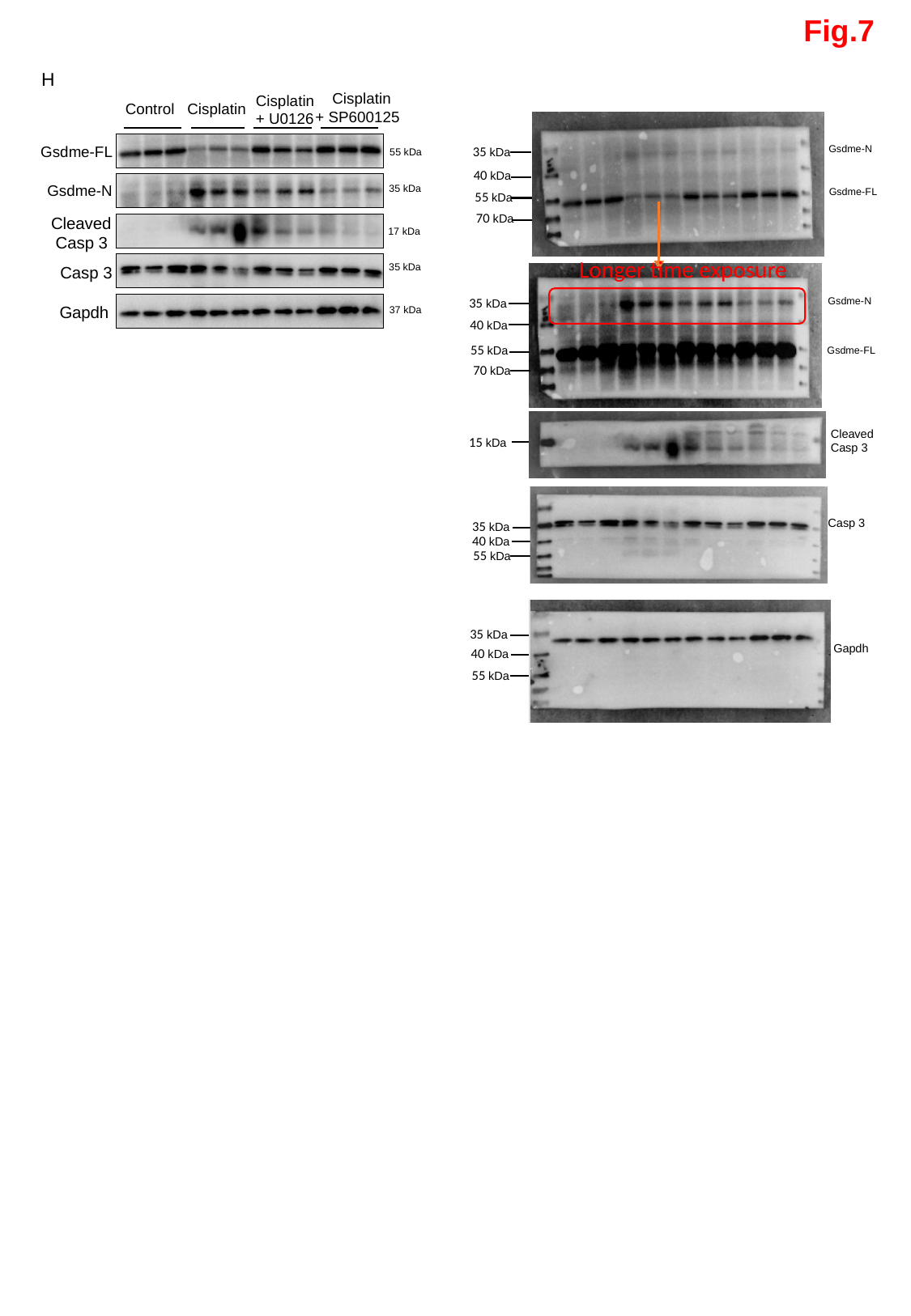

Fig.7
H
 Cisplatin
 + SP600125
Cisplatin
+ U0126
Control Cisplatin
Gsdme-FL
Gsdme-N
Cleaved
 Casp 3
Casp 3
Gapdh
55 kDa
35 kDa
17 kDa
35 kDa
37 kDa
Gsdme-N
35 kDa
40 kDa
Gsdme-FL
55 kDa
70 kDa
Longer time exposure
Gsdme-N
35 kDa
40 kDa
55 kDa
Gsdme-FL
70 kDa
Cleaved
Casp 3
15 kDa
 Casp 3
35 kDa
40 kDa
55 kDa
35 kDa
 Gapdh
40 kDa
55 kDa

## Slide 12
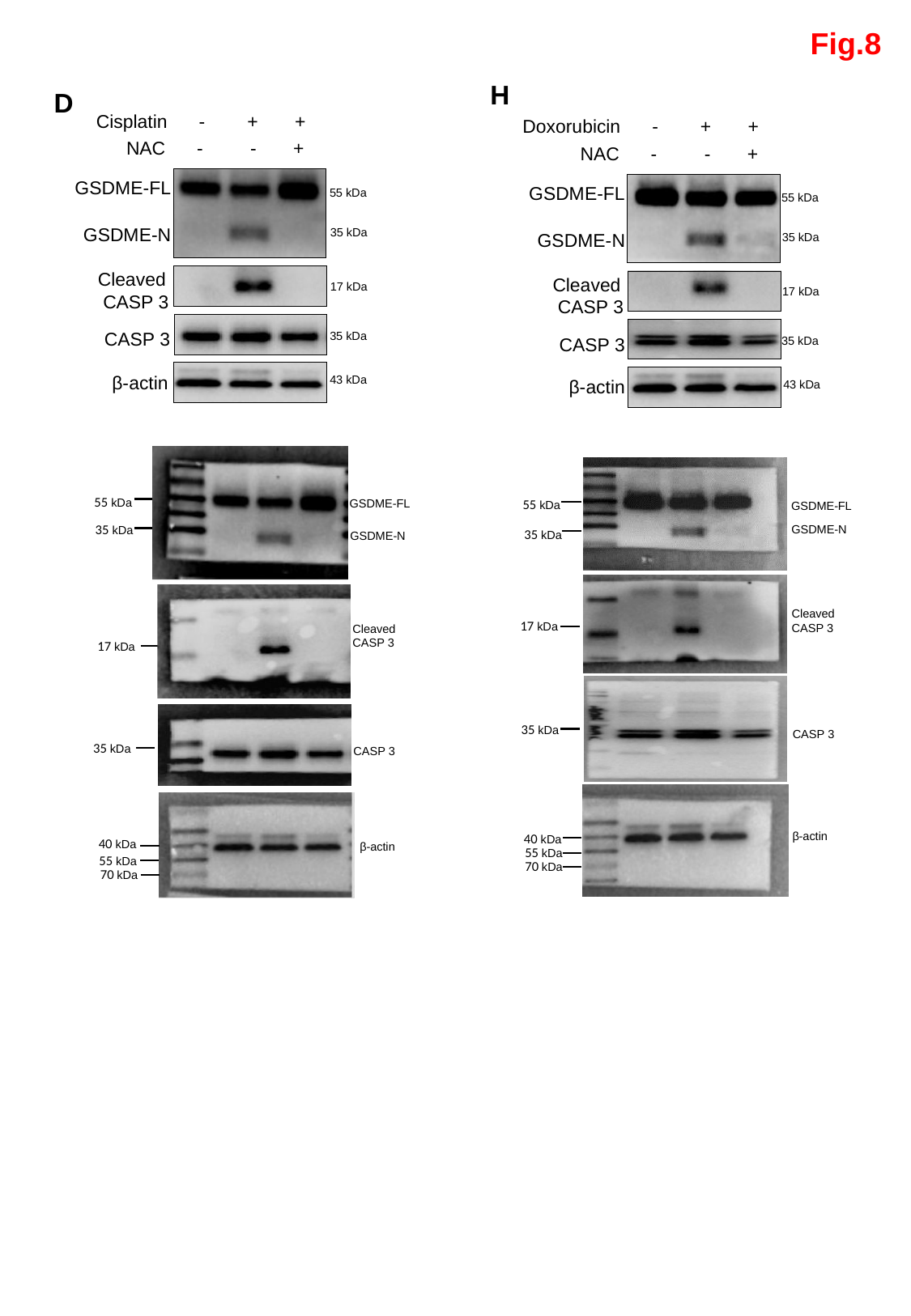

Fig.8
H
D
Cisplatin - + +
NAC - - +
GSDME-FL
GSDME-N
Cleaved
 CASP 3
CASP 3
β-actin
55 kDa
35 kDa
17 kDa
35 kDa
43 kDa
Doxorubicin - + +
NAC - - +
GSDME-FL
55 kDa
GSDME-N
35 kDa
Cleaved
 CASP 3
17 kDa
CASP 3
35 kDa
β-actin
43 kDa
55 kDa
GSDME-FL
55 kDa
GSDME-FL
35 kDa
GSDME-N
35 kDa
GSDME-N
Cleaved
CASP 3
17 kDa
Cleaved
CASP 3
17 kDa
35 kDa
 CASP 3
35 kDa
 CASP 3
β-actin
40 kDa
40 kDa
β-actin
55 kDa
55 kDa
70 kDa
70 kDa

## Slide 13
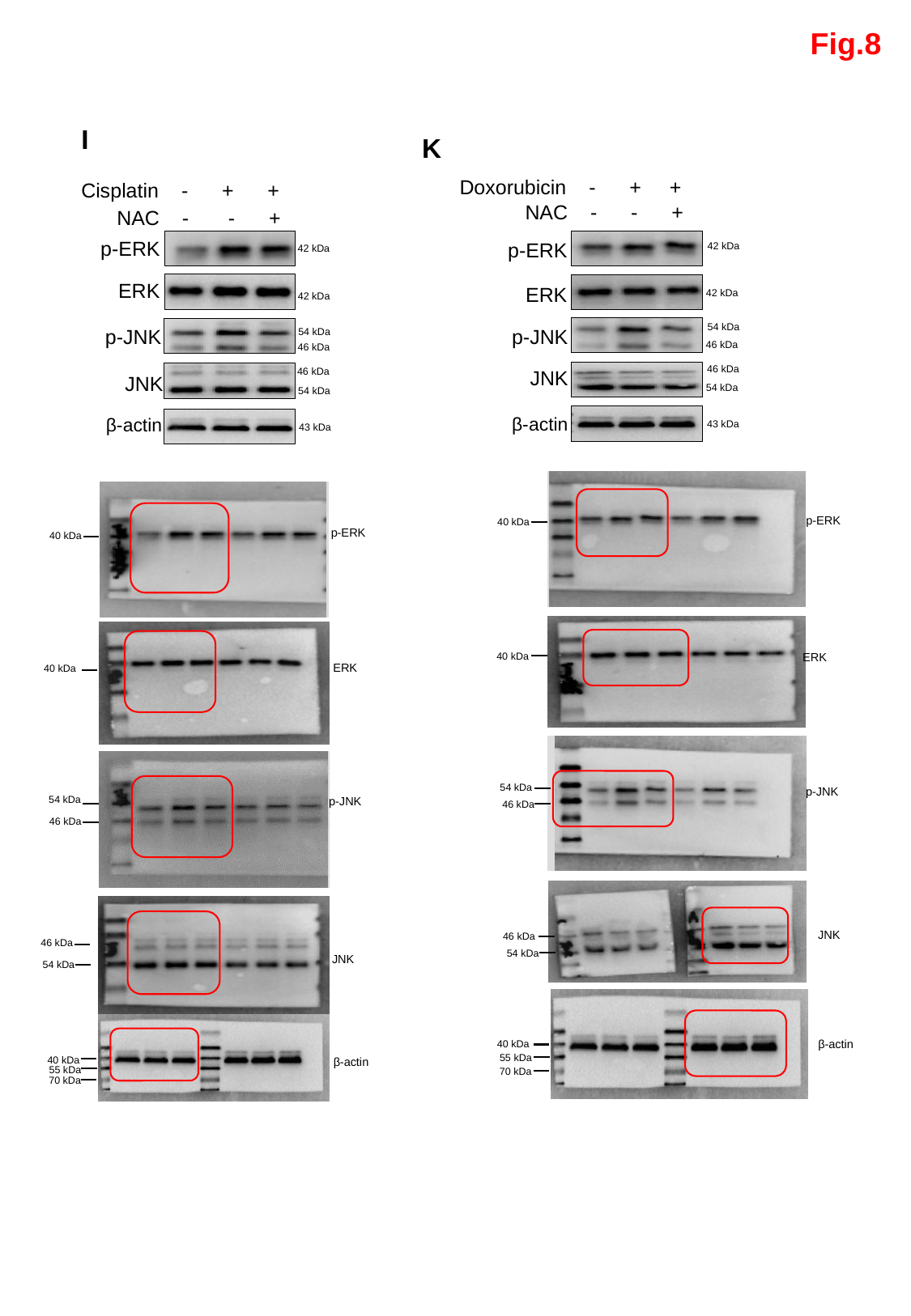

Fig.8
I
K
Doxorubicin - + +
NAC - - +
p-ERK
42 kDa
ERK
42 kDa
54 kDa
p-JNK
46 kDa
46 kDa
JNK
54 kDa
β-actin
43 kDa
Cisplatin - + +
NAC - - +
p-ERK
42 kDa
ERK
42 kDa
p-JNK
54 kDa
46 kDa
46 kDa
JNK
54 kDa
β-actin
43 kDa
p-ERK
40 kDa
p-ERK
40 kDa
40 kDa
ERK
ERK
40 kDa
54 kDa
p-JNK
46 kDa
54 kDa
p-JNK
46 kDa
46 kDa
JNK
54 kDa
JNK
46 kDa
54 kDa
40 kDa
β-actin
55 kDa
70 kDa
β-actin
40 kDa
55 kDa
70 kDa

## Slide 14
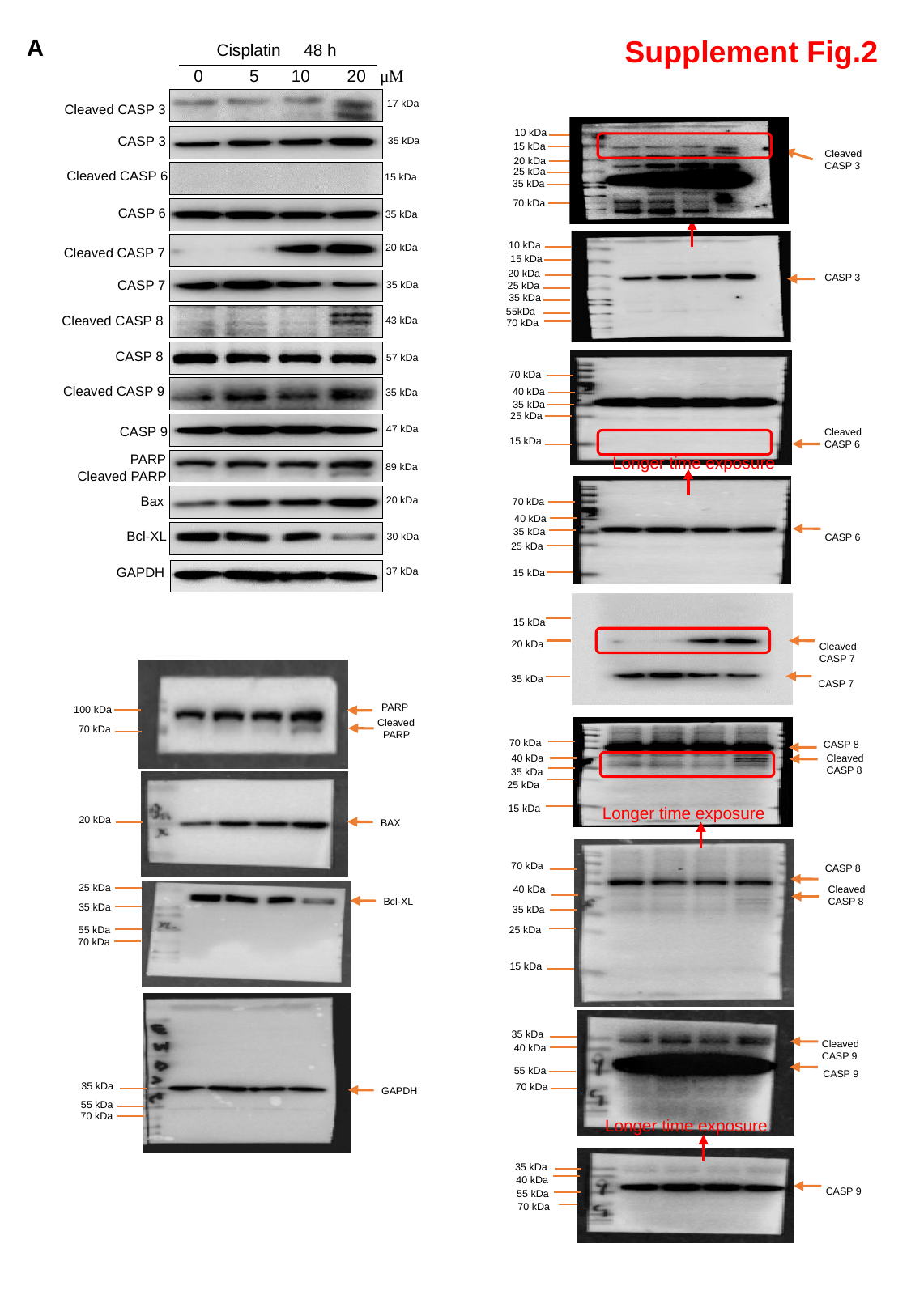

Supplement Fig.2
A
Cisplatin 48 h
0 5 10 20 μM
Cleaved CASP 3
CASP 3
Cleaved CASP 6
CASP 6
Cleaved CASP 7
CASP 7
Cleaved CASP 8
CASP 8
Cleaved CASP 9
CASP 9
PARP
Cleaved PARP
Bax
Bcl-XL
GAPDH
17 kDa
35 kDa
15 kDa
35 kDa
20 kDa
35 kDa
43 kDa
57 kDa
35 kDa
47 kDa
89 kDa
20 kDa
30 kDa
37 kDa
10 kDa
15 kDa
Cleaved
CASP 3
20 kDa
25 kDa
35 kDa
70 kDa
Longer time exposure
10 kDa
15 kDa
20 kDa
CASP 3
25 kDa
35 kDa
55kDa
70 kDa
70 kDa
40 kDa
35 kDa
25 kDa
Cleaved
CASP 6
15 kDa
Longer time exposure
70 kDa
40 kDa
35 kDa
CASP 6
25 kDa
15 kDa
15 kDa
20 kDa
35 kDa
Cleaved
CASP 7
CASP 7
PARP
100 kDa
Cleaved
 PARP
70 kDa
70 kDa
40 kDa
35 kDa
25 kDa
15 kDa
 CASP 8
Cleaved
CASP 8
20 kDa
BAX
Longer time exposure
70 kDa
40 kDa
35 kDa
25 kDa
15 kDa
 CASP 8
Cleaved
CASP 8
25 kDa
Bcl-XL
35 kDa
55 kDa
70 kDa
35 kDa
GAPDH
55 kDa
70 kDa
35 kDa
Cleaved
CASP 9
40 kDa
55 kDa
 CASP 9
70 kDa
Longer time exposure
35 kDa
40 kDa
 CASP 9
55 kDa
70 kDa

## Slide 15
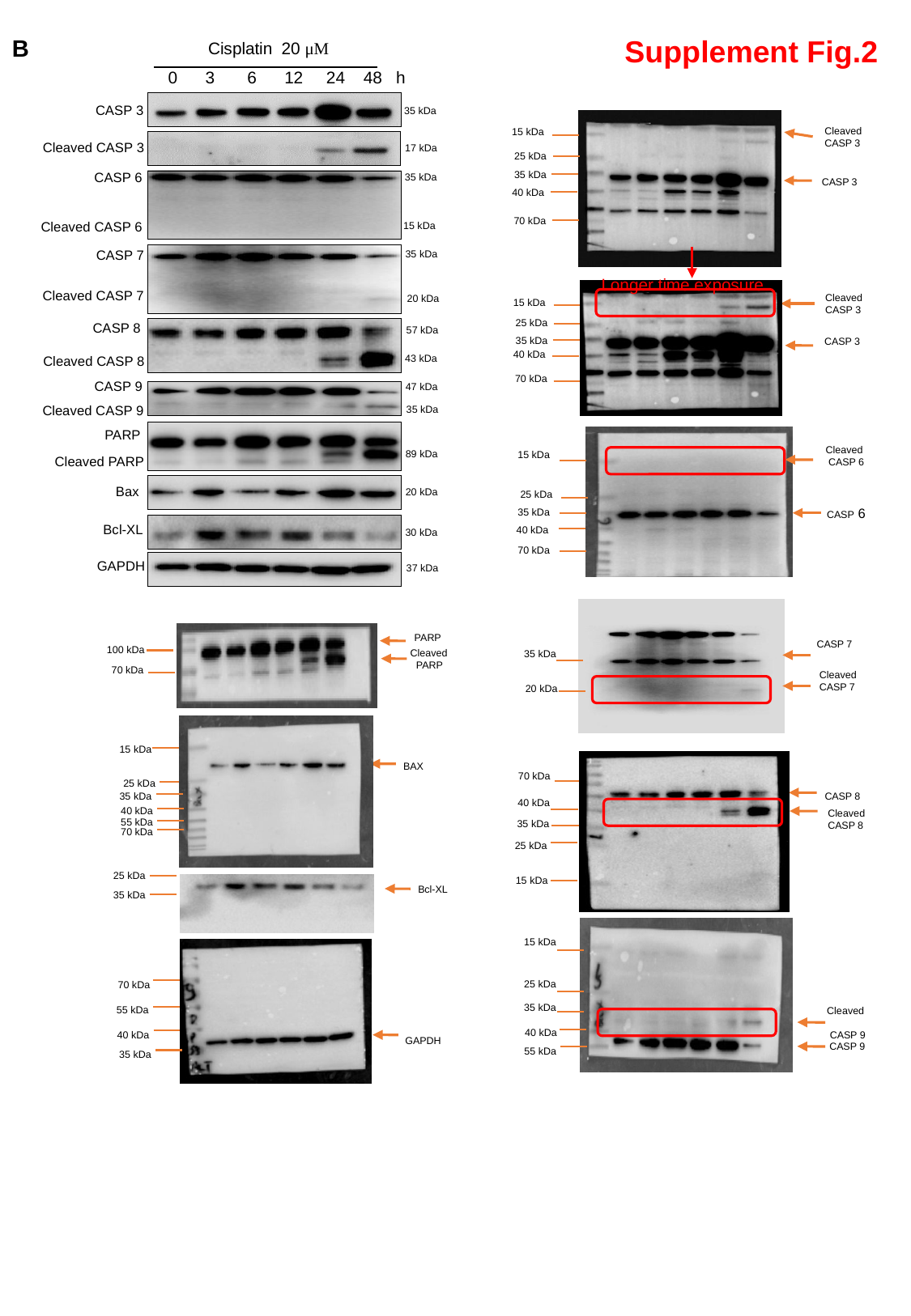

Supplement Fig.2
B
Cisplatin 20 μM
0 3 6 12 24 48 h
CASP 3
Cleaved CASP 3
CASP 6
Cleaved CASP 6
CASP 7
Cleaved CASP 7
CASP 8
Cleaved CASP 8
CASP 9
Cleaved CASP 9
PARP
Cleaved PARP
Bax
Bcl-XL
GAPDH
35 kDa
17 kDa
35 kDa
15 kDa
35 kDa
20 kDa
57 kDa
43 kDa
47 kDa
35 kDa
89 kDa
20 kDa
30 kDa
37 kDa
Cleaved
CASP 3
15 kDa
25 kDa
35 kDa
CASP 3
40 kDa
70 kDa
Longer time exposure
Cleaved
CASP 3
15 kDa
25 kDa
35 kDa
CASP 3
40 kDa
70 kDa
15 kDa
25 kDa
35 kDa
40 kDa
70 kDa
Cleaved
 CASP 6
CASP 6
CASP 7
35 kDa
Cleaved CASP 7
20 kDa
PARP
100 kDa
Cleaved
 PARP
70 kDa
15 kDa
BAX
25 kDa
35 kDa
40 kDa
55 kDa
70 kDa
70 kDa
 CASP 8
40 kDa
Cleaved
CASP 8
35 kDa
25 kDa
15 kDa
25 kDa
Bcl-XL
35 kDa
15 kDa
25 kDa
35 kDa
 Cleaved
 CASP 9
40 kDa
 CASP 9
55 kDa
70 kDa
55 kDa
40 kDa
GAPDH
35 kDa

## Slide 16
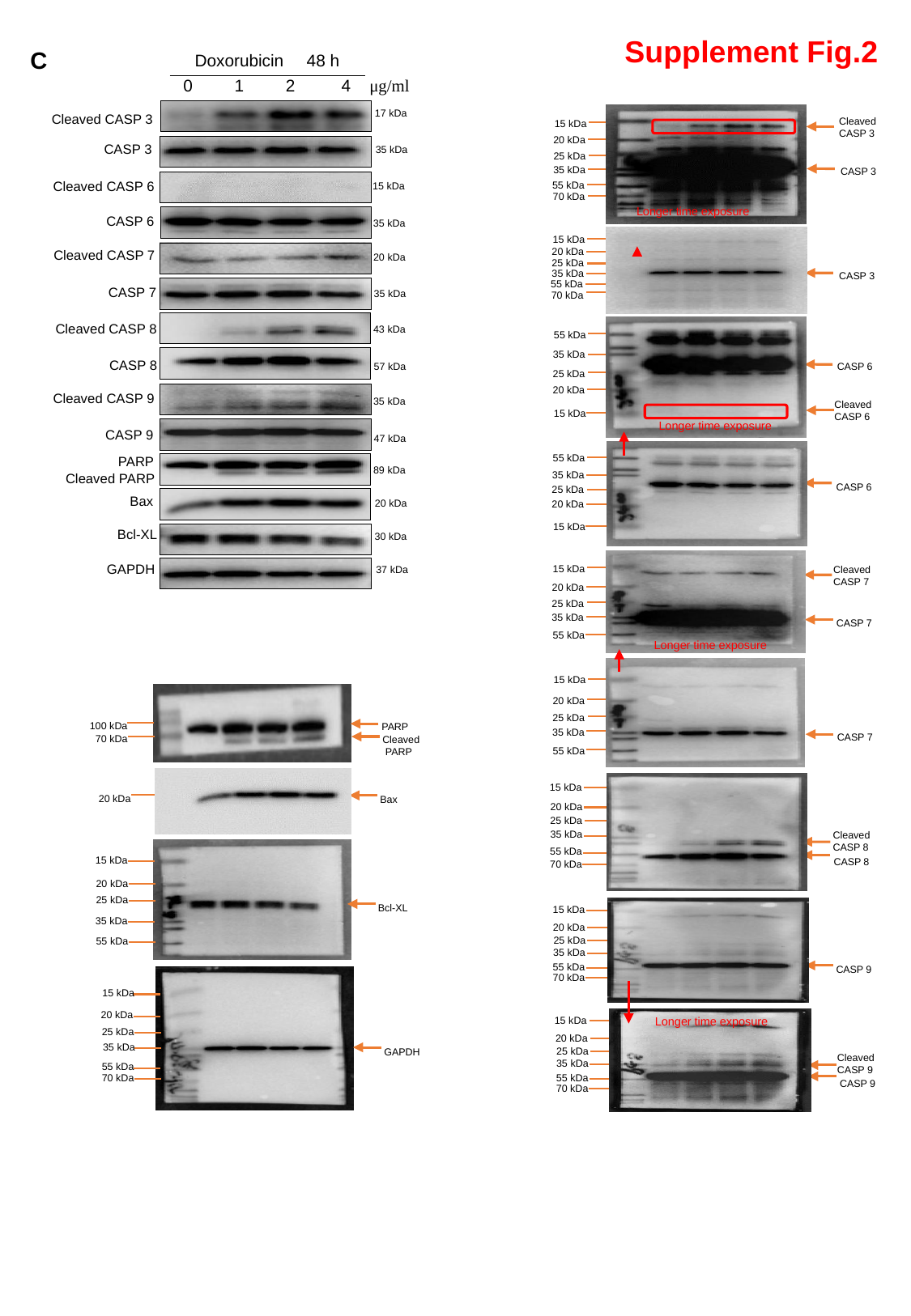

Supplement Fig.2
C
Doxorubicin 48 h
0 1 2 4 μg/ml
17 kDa
Cleaved CASP 3
CASP 3
35 kDa
Cleaved CASP 6
15 kDa
CASP 6
35 kDa
Cleaved CASP 7
20 kDa
CASP 7
35 kDa
Cleaved CASP 8
43 kDa
CASP 8
57 kDa
Cleaved CASP 9
35 kDa
CASP 9
47 kDa
PARP
89 kDa
Cleaved PARP
Bax
20 kDa
Bcl-XL
30 kDa
GAPDH
37 kDa
Cleaved
CASP 3
15 kDa
20 kDa
25 kDa
35 kDa
CASP 3
55 kDa
70 kDa
Longer time exposure
15 kDa
20 kDa
25 kDa
35 kDa
CASP 3
55 kDa
70 kDa
55 kDa
35 kDa
CASP 6
25 kDa
20 kDa
Cleaved
CASP 6
15 kDa
Longer time exposure
55 kDa
35 kDa
CASP 6
25 kDa
20 kDa
15 kDa
15 kDa
Cleaved
CASP 7
20 kDa
25 kDa
35 kDa
CASP 7
55 kDa
Longer time exposure
15 kDa
20 kDa
25 kDa
35 kDa
CASP 7
55 kDa
100 kDa
PARP
70 kDa
Cleaved
 PARP
20 kDa
Bax
15 kDa
20 kDa
25 kDa
35 kDa
Cleaved
CASP 8
55 kDa
CASP 8
70 kDa
15 kDa
20 kDa
25 kDa
Bcl-XL
35 kDa
55 kDa
15 kDa
20 kDa
25 kDa
35 kDa
55 kDa
CASP 9
70 kDa
Longer time exposure
15 kDa
20 kDa
25 kDa
Cleaved
CASP 9
35 kDa
55 kDa
CASP 9
70 kDa
15 kDa
20 kDa
25 kDa
35 kDa
GAPDH
55 kDa
70 kDa

## Slide 17
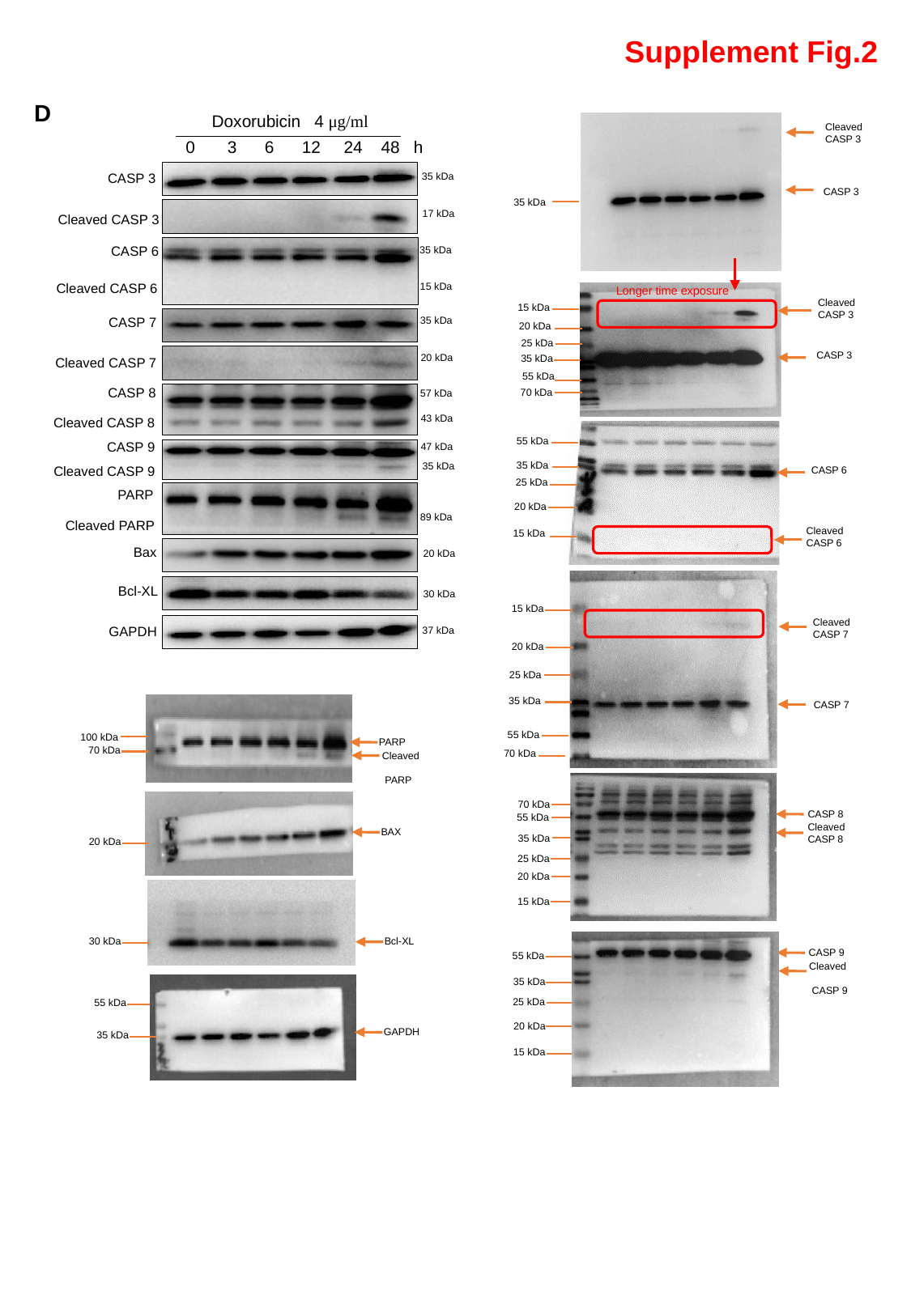

Supplement Fig.2
D
Doxorubicin 4 μg/ml
0 3 6 12 24 48 h
CASP 3
Cleaved CASP 3
CASP 6
Cleaved CASP 6
CASP 7
Cleaved CASP 7
CASP 8
Cleaved CASP 8
CASP 9
Cleaved CASP 9
PARP
Cleaved PARP
Bax
Bcl-XL
GAPDH
35 kDa
17 kDa
35 kDa
15 kDa
35 kDa
20 kDa
57 kDa
43 kDa
47 kDa
35 kDa
89 kDa
20 kDa
30 kDa
37 kDa
Cleaved
CASP 3
35 kDa
CASP 3
Longer time exposure
15 kDa
20 kDa
25 kDa
35 kDa
55 kDa
70 kDa
Cleaved
CASP 3
CASP 3
55 kDa
35 kDa
25 kDa
20 kDa
15 kDa
CASP 6
Cleaved
CASP 6
15 kDa
Cleaved CASP 7
20 kDa
25 kDa
35 kDa
CASP 7
55 kDa
70 kDa
100 kDa
PARP
70 kDa
 Cleaved
 PARP
70 kDa
55 kDa
35 kDa
25 kDa
20 kDa
15 kDa
 CASP 8
Cleaved
CASP 8
BAX
20 kDa
30 kDa
Bcl-XL
 CASP 9
55 kDa
 Cleaved
 CASP 9
35 kDa
25 kDa
20 kDa
15 kDa
55 kDa
GAPDH
35 kDa

## Slide 18
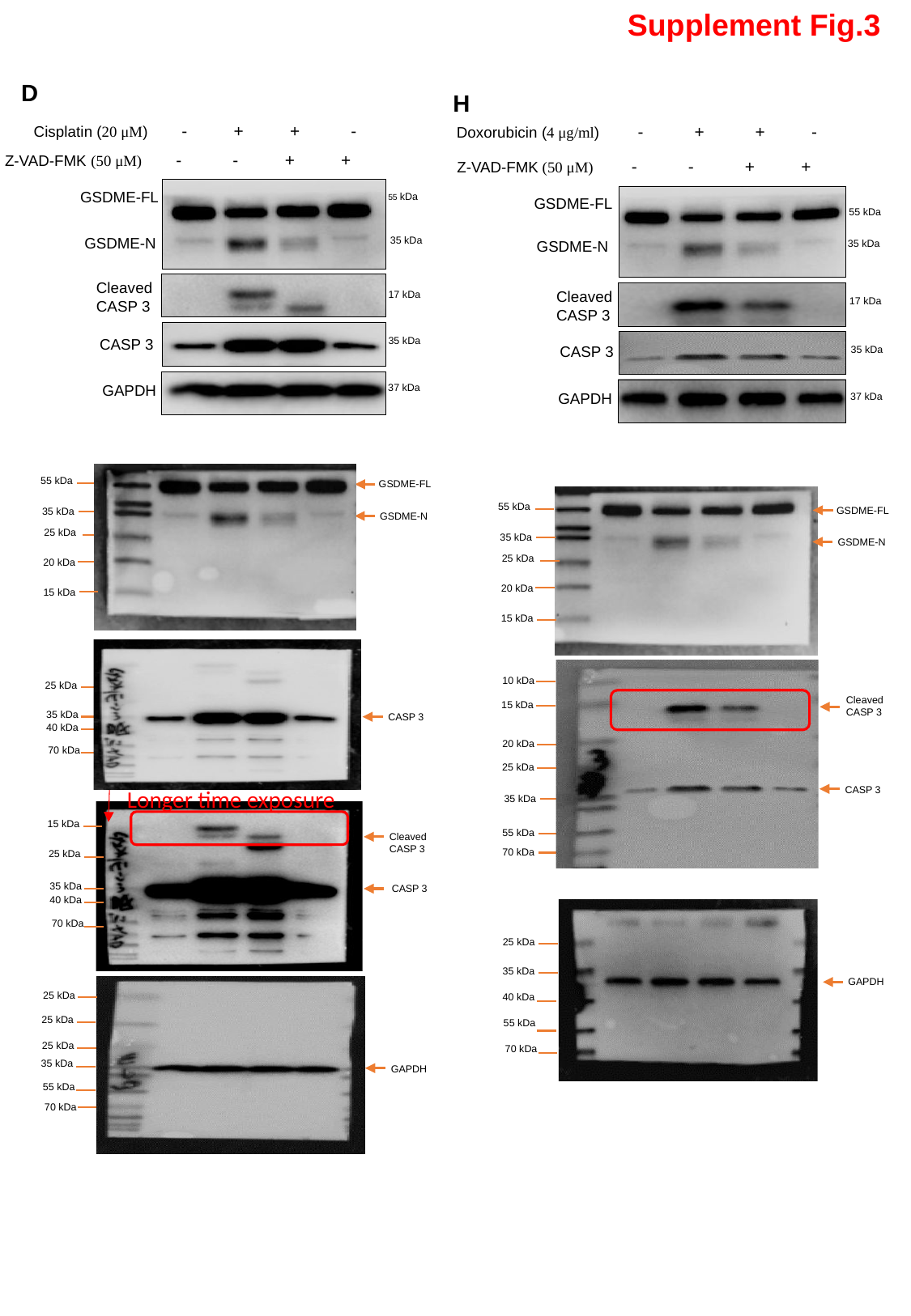

Supplement Fig.3
D
Cisplatin (20 μM) - + + -
Z-VAD-FMK (50 μM) - - + +
GSDME-FL
55 kDa
GSDME-N
35 kDa
Cleaved
CASP 3
17 kDa
35 kDa
CASP 3
37 kDa
GAPDH
H
Doxorubicin (4 μg/ml) - + + -
Z-VAD-FMK (50 μM) - - + +
GSDME-FL
55 kDa
GSDME-N
35 kDa
Cleaved
CASP 3
17 kDa
CASP 3
35 kDa
GAPDH
37 kDa
55 kDa
GSDME-FL
35 kDa
GSDME-N
25 kDa
20 kDa
15 kDa
55 kDa
GSDME-FL
35 kDa
GSDME-N
25 kDa
20 kDa
15 kDa
10 kDa
CleavedCASP 3
15 kDa
20 kDa
25 kDa
CASP 3
35 kDa
55 kDa
70 kDa
25 kDa
35 kDa
CASP 3
40 kDa
70 kDa
Longer time exposure
15 kDa
CleavedCASP 3
25 kDa
35 kDa
CASP 3
40 kDa
70 kDa
25 kDa
35 kDa
GAPDH
40 kDa
55 kDa
70 kDa
25 kDa
25 kDa
25 kDa
35 kDa
GAPDH
55 kDa
70 kDa

## Slide 19
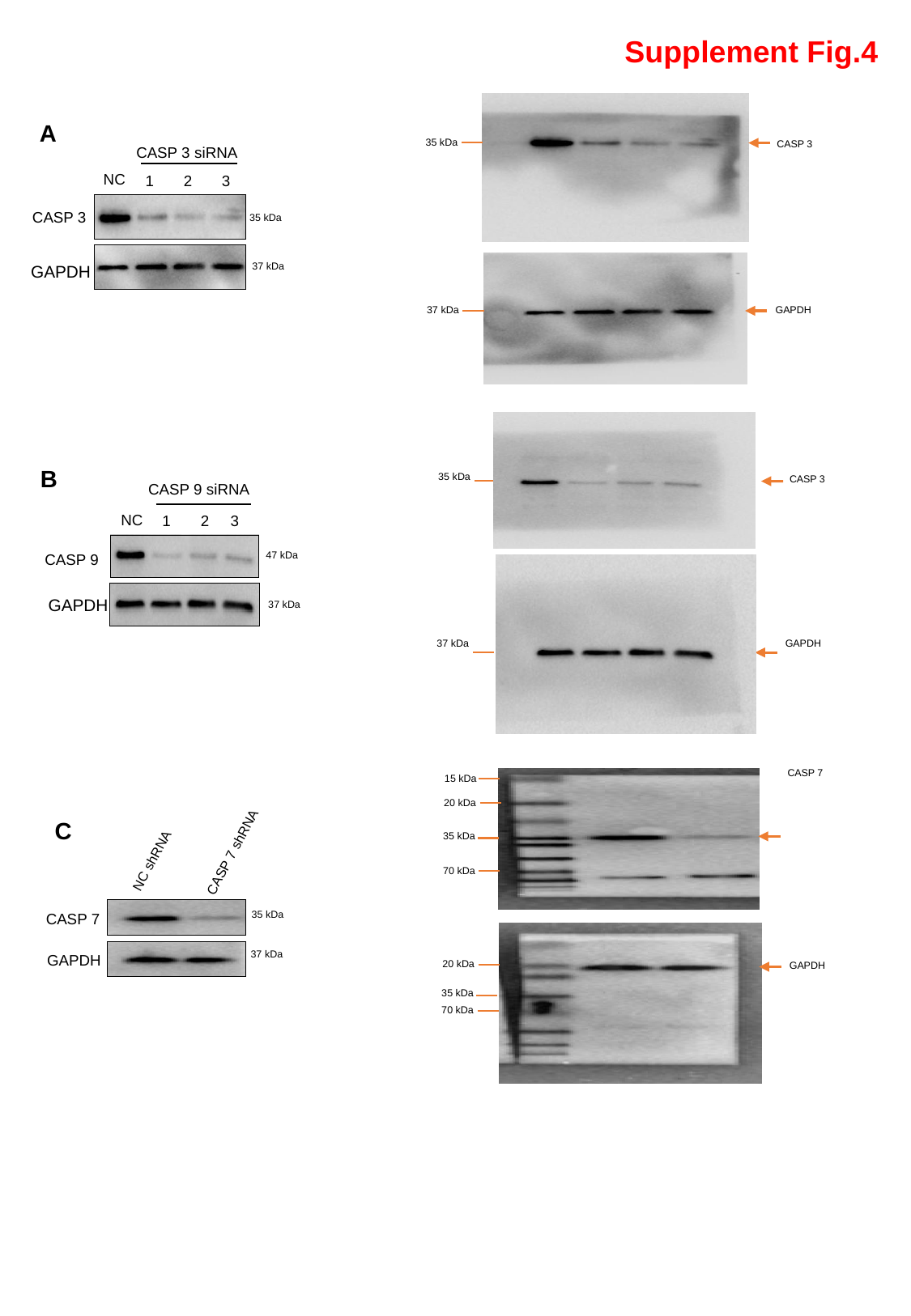

Supplement Fig.4
35 kDa
CASP 3
A
CASP 3 siRNA
NC
 2 3
CASP 3
35 kDa
37 kDa
GAPDH
37 kDa
GAPDH
35 kDa
CASP 3
B
CASP 9 siRNA
NC
 2 3
47 kDa
CASP 9
GAPDH
37 kDa
GAPDH
37 kDa
CASP 7
15 kDa
20 kDa
35 kDa
70 kDa
C
CASP 7 shRNA
NC shRNA
35 kDa
CASP 7
37 kDa
GAPDH
20 kDa
GAPDH
35 kDa
70 kDa

## Slide 20
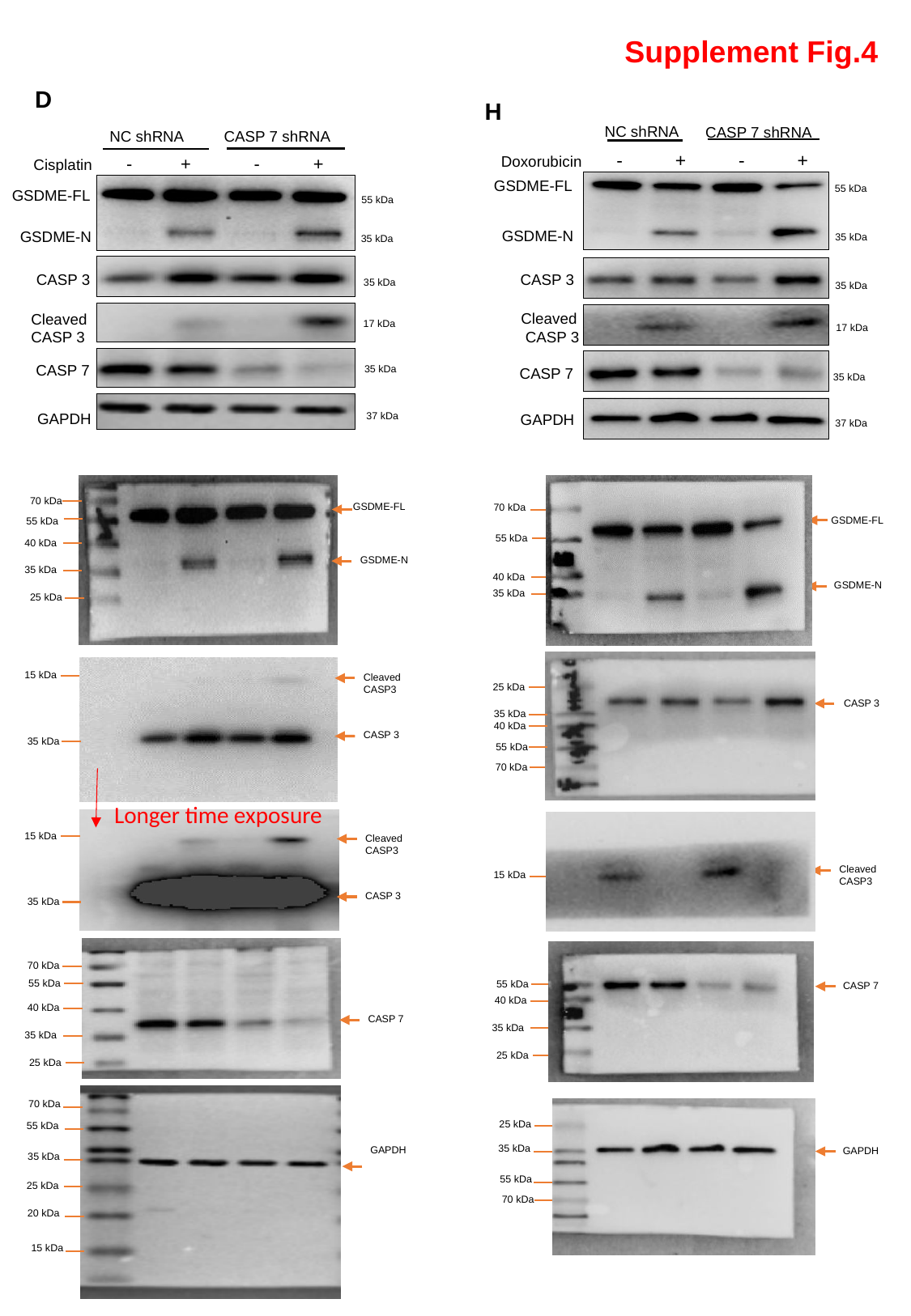

Supplement Fig.4
D
H
NC shRNA
CASP 7 shRNA
Doxorubicin - + - +
GSDME-FL
GSDME-N
CASP 3
Cleaved
 CASP 3
CASP 7
GAPDH
55 kDa
35 kDa
35 kDa
17 kDa
35 kDa
37 kDa
NC shRNA
CASP 7 shRNA
Cisplatin - + - +
GSDME-FL
GSDME-N
CASP 3
Cleaved
CASP 3
CASP 7
GAPDH
55 kDa
35 kDa
35 kDa
17 kDa
35 kDa
37 kDa
70 kDa
GSDME-FL
55 kDa
40 kDa
GSDME-N
35 kDa
70 kDa
GSDME-FL
55 kDa
40 kDa
GSDME-N
35 kDa
25 kDa
25 kDa
CASP 3
35 kDa
40 kDa
55 kDa
70 kDa
15 kDa
Cleaved CASP3
CASP 3
35 kDa
Longer time exposure
15 kDa
Cleaved CASP3
CASP 3
35 kDa
Cleaved CASP3
15 kDa
70 kDa
55 kDa
40 kDa
CASP 7
35 kDa
25 kDa
55 kDa
CASP 7
40 kDa
35 kDa
25 kDa
70 kDa
55 kDa
35 kDa
25 kDa
20 kDa
15 kDa
25 kDa
35 kDa
GAPDH
55 kDa
70 kDa
GAPDH

## Slide 21
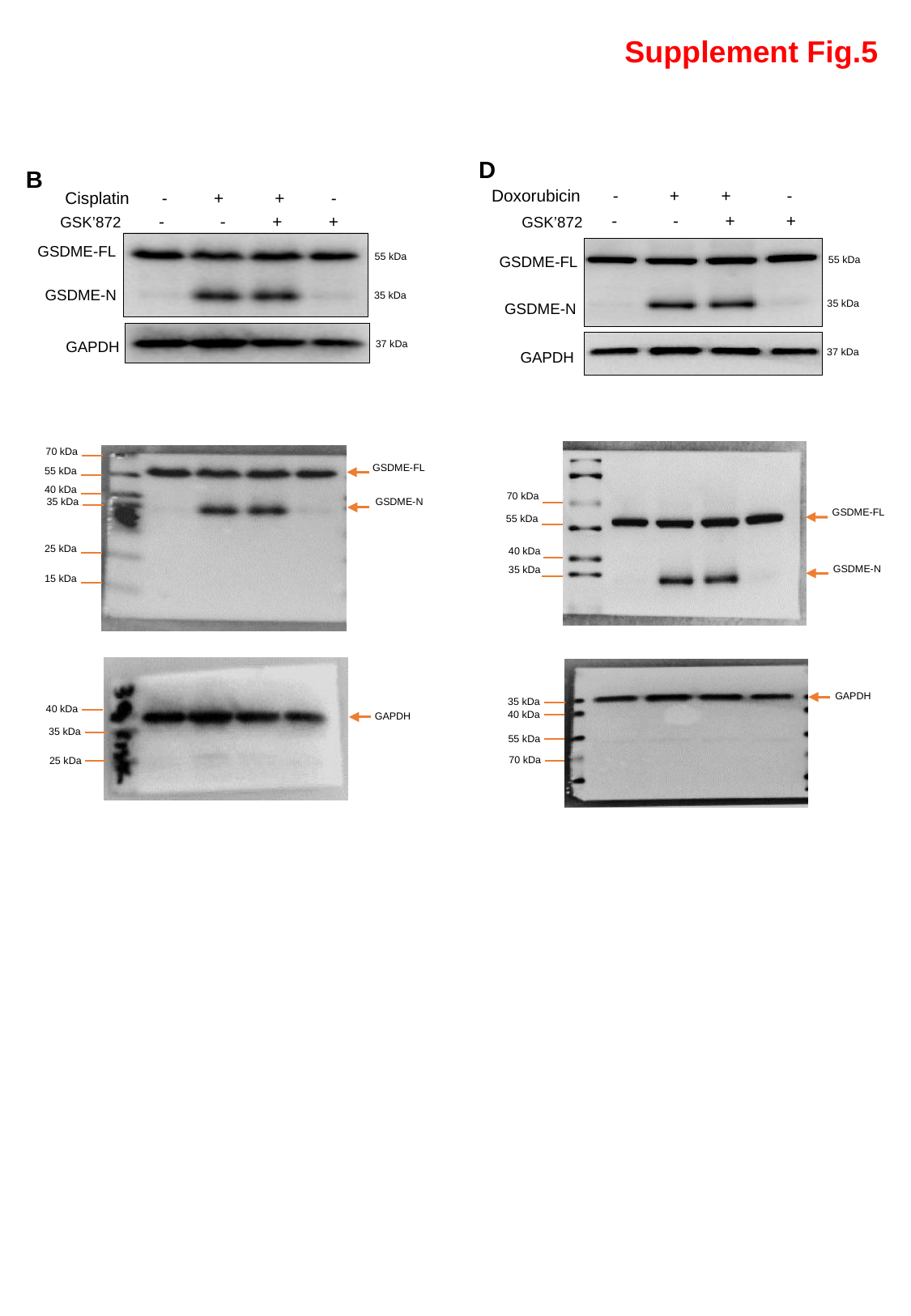

Supplement Fig.5
D
Doxorubicin - + + -
 - - + +
GSDME-FL
GSDME-N
GAPDH
55 kDa
35 kDa
37 kDa
B
Cisplatin - + + -
 - - + +
GSDME-FL
GSDME-N
GAPDH
55 kDa
35 kDa
37 kDa
GSK’872
GSK’872
70 kDa
GSDME-FL
55 kDa
40 kDa
35 kDa
GSDME-N
25 kDa
15 kDa
70 kDa
GSDME-FL
55 kDa
40 kDa
GSDME-N
35 kDa
40 kDa
GAPDH
35 kDa
25 kDa
GAPDH
35 kDa
40 kDa
55 kDa
70 kDa

## Slide 22
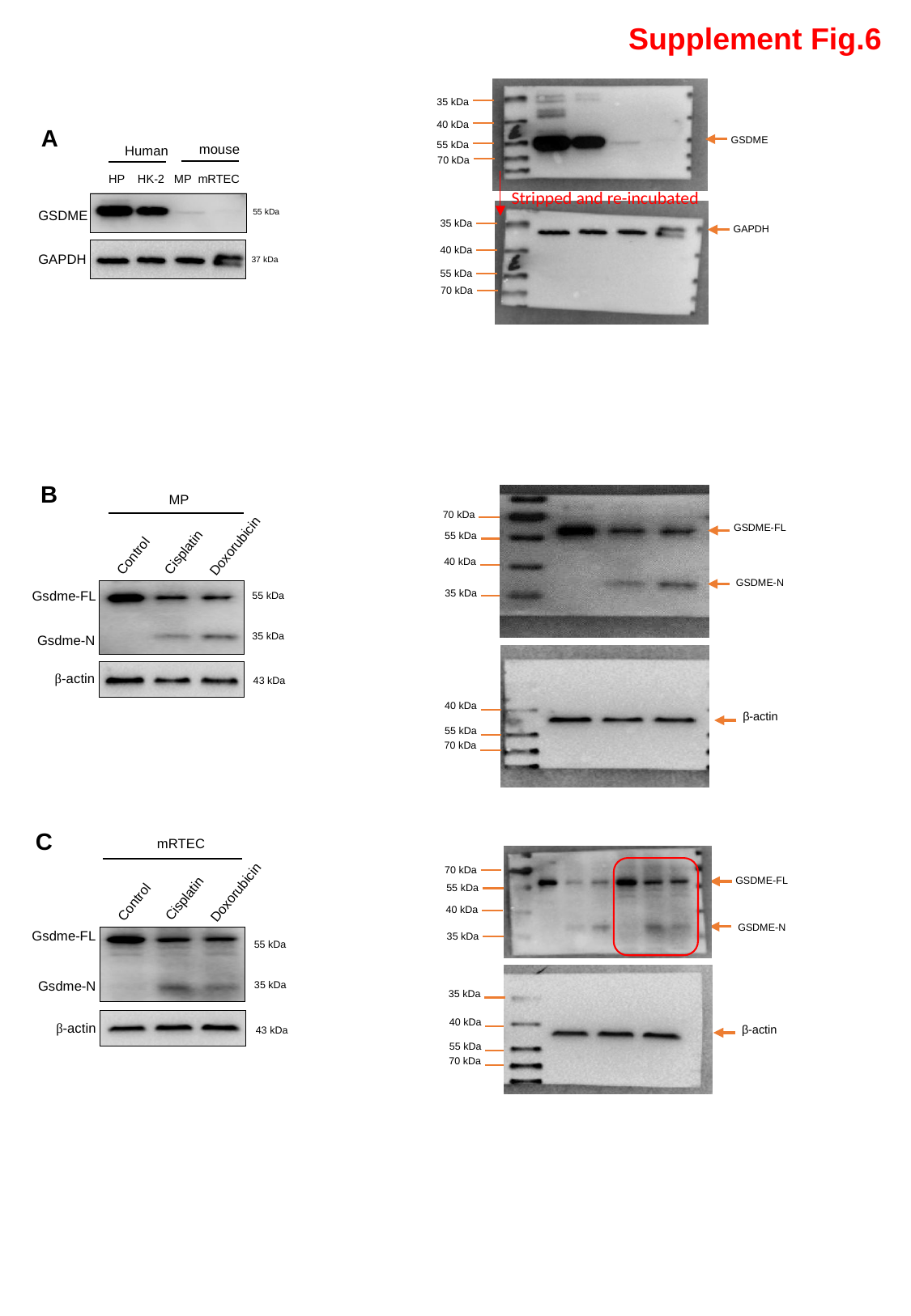

Supplement Fig.6
35 kDa
40 kDa
GSDME
55 kDa
70 kDa
A
mouse
Human
HP HK-2 MP mRTEC
55 kDa
GSDME
GAPDH
37 kDa
Stripped and re-incubated
35 kDa
GAPDH
40 kDa
55 kDa
70 kDa
B
MP
Doxorubicin
Cisplatin
Control
Gsdme-FL
Gsdme-N
β-actin
55 kDa
35 kDa
43 kDa
70 kDa
GSDME-FL
55 kDa
40 kDa
GSDME-N
35 kDa
40 kDa
β-actin
55 kDa
70 kDa
C
mRTEC
Doxorubicin
Cisplatin
Control
Gsdme-FL
55 kDa
Gsdme-N
35 kDa
β-actin
43 kDa
70 kDa
GSDME-FL
55 kDa
40 kDa
GSDME-N
35 kDa
40 kDa
β-actin
55 kDa
70 kDa
35 kDa

## Slide 23
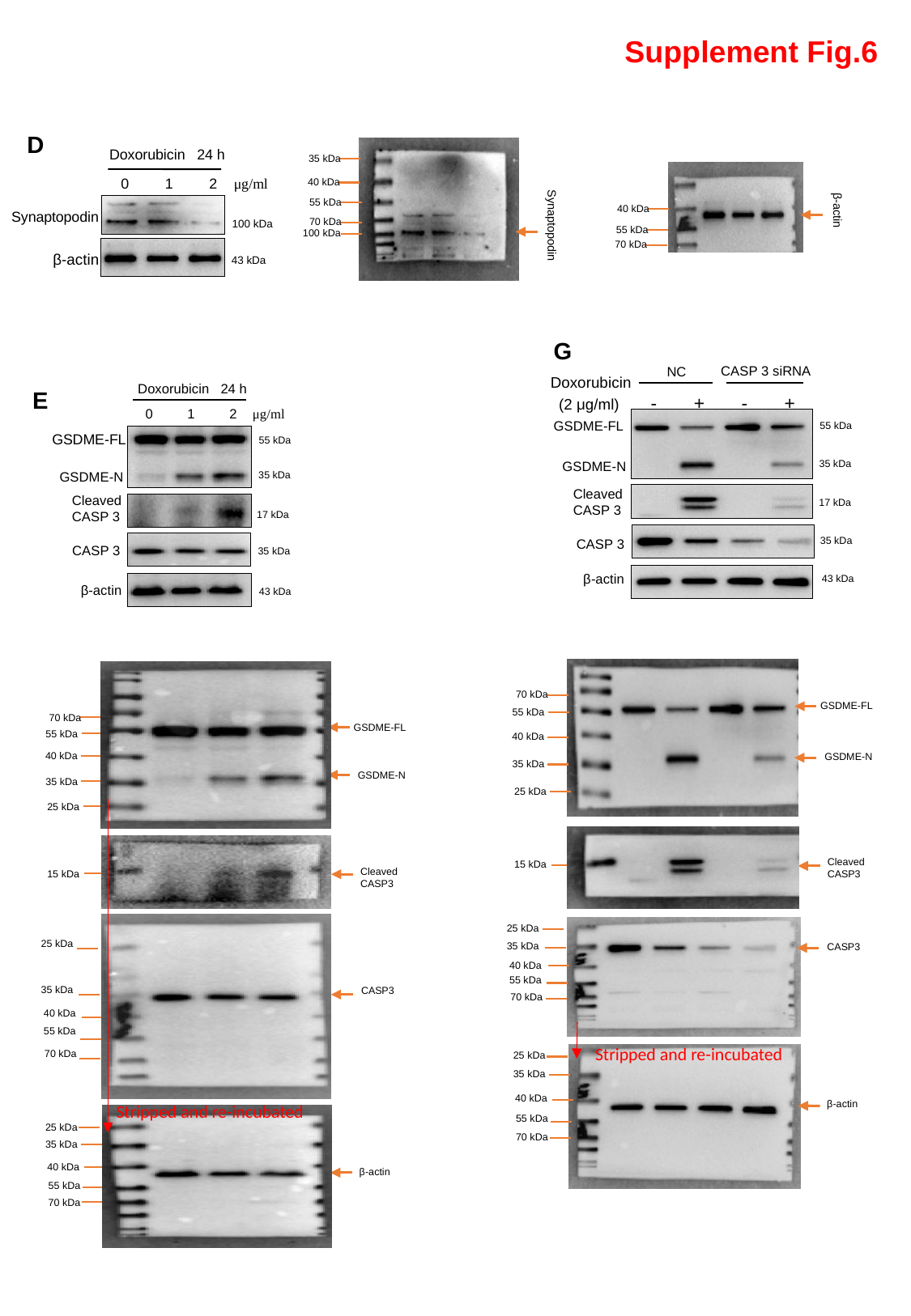

Supplement Fig.6
D
Doxorubicin 24 h
0 1 2 μg/ml
Synaptopodin
100 kDa
β-actin
43 kDa
35 kDa
40 kDa
55 kDa
70 kDa
Synaptopodin
100 kDa
40 kDa
β-actin
55 kDa
70 kDa
G
CASP 3 siRNA
NC
Doxorubicin
 (2 μg/ml) - + - +
GSDME-FL
55 kDa
35 kDa
GSDME-N
Cleaved
CASP 3
17 kDa
35 kDa
CASP 3
β-actin
43 kDa
Doxorubicin 24 h
0 1 2 μg/ml
55 kDa
GSDME-N
35 kDa
Cleaved CASP 3
17 kDa
CASP 3
35 kDa
β-actin
43 kDa
E
GSDME-FL
70 kDa
GSDME-FL
55 kDa
40 kDa
GSDME-N
35 kDa
25 kDa
70 kDa
GSDME-FL
55 kDa
40 kDa
GSDME-N
35 kDa
25 kDa
Cleaved CASP3
15 kDa
Cleaved CASP3
15 kDa
25 kDa
35 kDa
CASP3
40 kDa
55 kDa
70 kDa
25 kDa
35 kDa
CASP3
40 kDa
55 kDa
70 kDa
Stripped and re-incubated
25 kDa
35 kDa
40 kDa
β-actin
55 kDa
70 kDa
Stripped and re-incubated
25 kDa
35 kDa
40 kDa
β-actin
55 kDa
70 kDa

## Slide 24
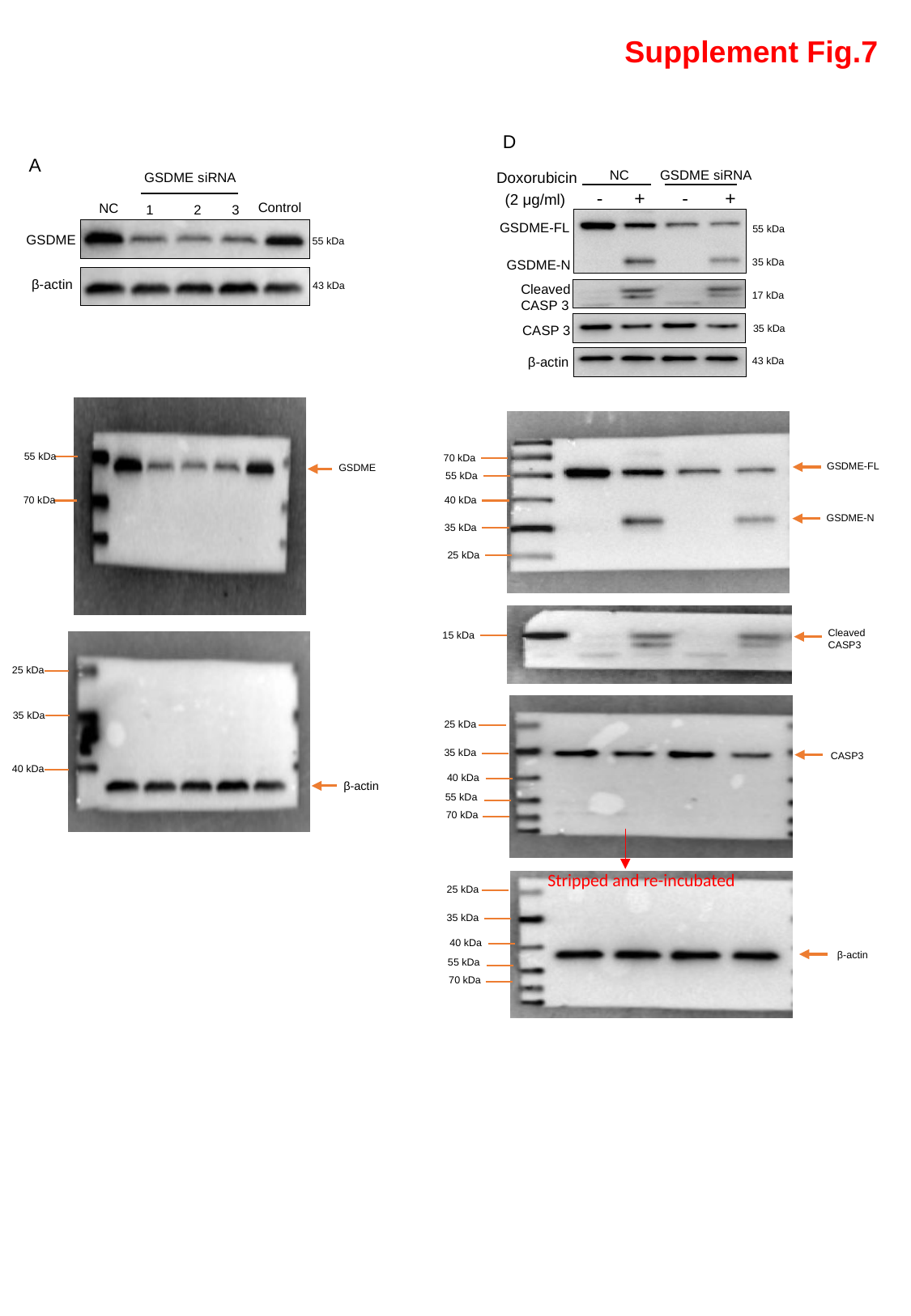

Supplement Fig.7
D
A
NC
GSDME siRNA
Doxorubicin
 (2 μg/ml) - + - +
GSDME siRNA
Control
NC
 2 3
GSDME
55 kDa
β-actin
43 kDa
GSDME-FL
55 kDa
35 kDa
GSDME-N
Cleaved
CASP 3
17 kDa
35 kDa
CASP 3
β-actin
43 kDa
55 kDa
GSDME
70 kDa
70 kDa
55 kDa
40 kDa
35 kDa
25 kDa
GSDME-FL
GSDME-N
Cleaved CASP3
15 kDa
25 kDa
35 kDa
40 kDa
β-actin
25 kDa
35 kDa
CASP3
40 kDa
55 kDa
70 kDa
Stripped and re-incubated
25 kDa
35 kDa
40 kDa
β-actin
55 kDa
70 kDa

## Slide 25
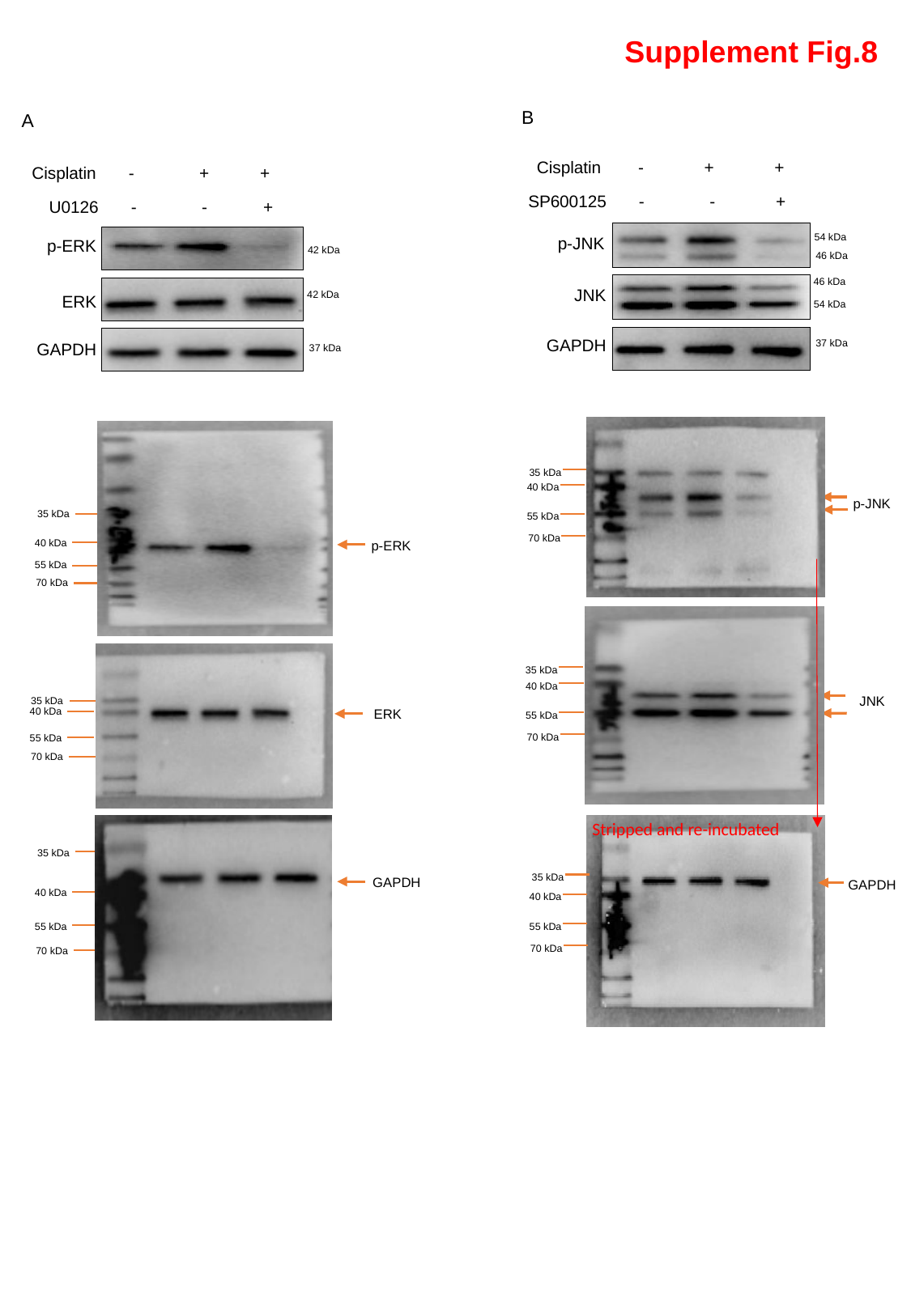

Supplement Fig.8
B
A
Cisplatin - + +
SP600125 - - +
p-JNK
JNK
GAPDH
54 kDa
46 kDa
46 kDa
54 kDa
37 kDa
Cisplatin - + +
U0126 - - +
p-ERK
ERK
GAPDH
42 kDa
42 kDa
37 kDa
35 kDa
40 kDa
p-JNK
55 kDa
70 kDa
35 kDa
40 kDa
p-ERK
55 kDa
70 kDa
35 kDa
40 kDa
JNK
55 kDa
70 kDa
35 kDa
40 kDa
55 kDa
70 kDa
ERK
Stripped and re-incubated
35 kDa
GAPDH
40 kDa
55 kDa
70 kDa
35 kDa
GAPDH
40 kDa
55 kDa
70 kDa

## Slide 26
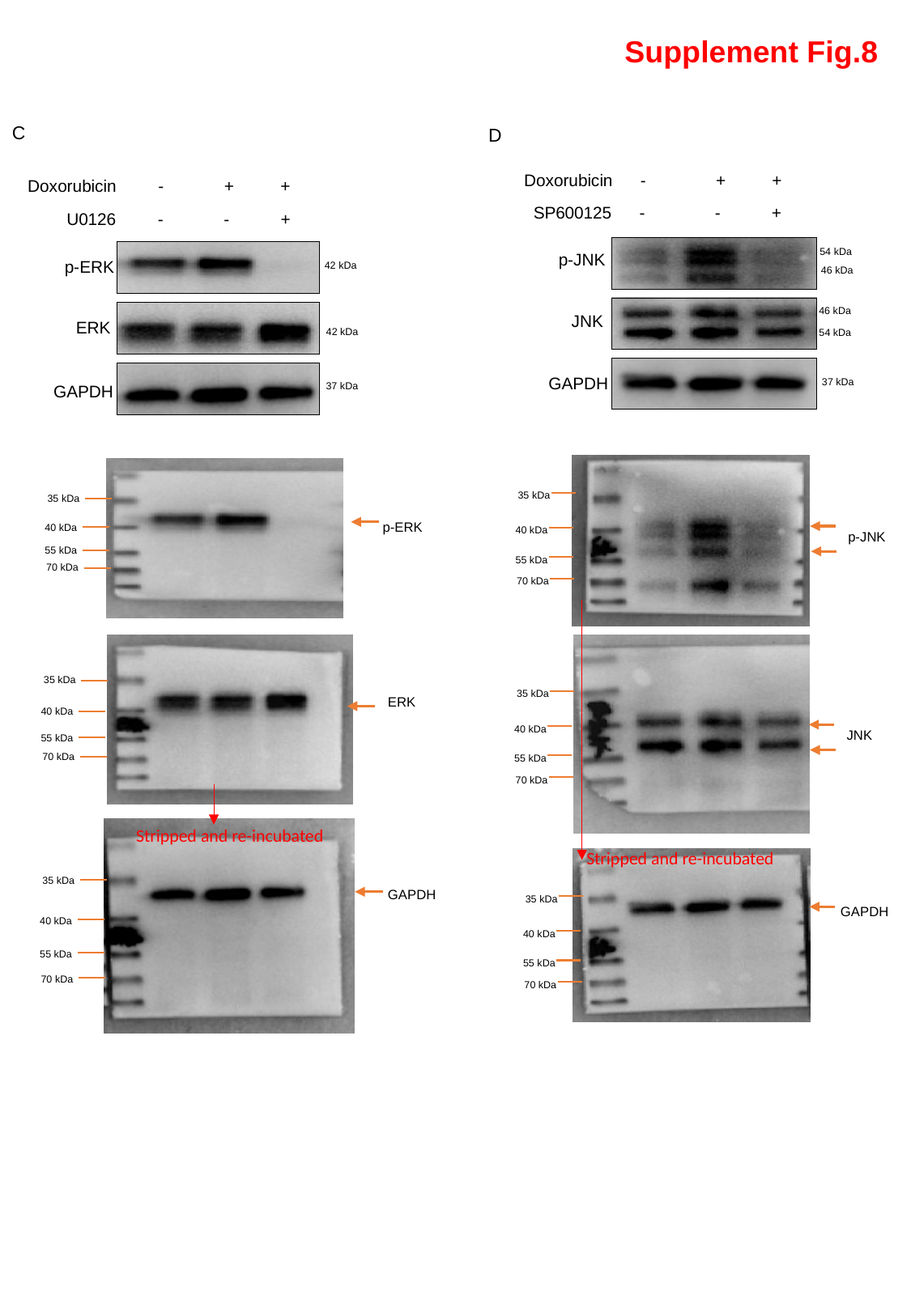

Supplement Fig.8
C
D
Doxorubicin - + +
SP600125 - - +
p-JNK
JNK
GAPDH
54 kDa
46 kDa
46 kDa
54 kDa
37 kDa
Doxorubicin - + +
U0126 - - +
p-ERK
ERK
GAPDH
42 kDa
42 kDa
37 kDa
35 kDa
40 kDa
p-JNK
55 kDa
70 kDa
35 kDa
p-ERK
40 kDa
55 kDa
70 kDa
35 kDa
40 kDa
JNK
55 kDa
70 kDa
35 kDa
40 kDa
55 kDa
70 kDa
ERK
Stripped and re-incubated
35 kDa
GAPDH
40 kDa
55 kDa
70 kDa
Stripped and re-incubated
35 kDa
GAPDH
40 kDa
55 kDa
70 kDa
